# The Arabidopsis SPFH protein HIR2 modulates receptor signaling and plasma membrane organization

**DOI:** 10.1038/s44318-026-00877-y

**Published:** 2026-07-29

**Authors:** Hannah Weber, Alexandra Ehinger, Dagmar Kolb, Vahid Fallahzadeh-Mamaghani, Thierry Halter, Mauricio P Contreras, Mirita Franz, Nora Schäfer, Sven zur Oven-Krockhaus, Julien Gronnier, Claudia Oecking, Cyril Zipfel, Klaus Harter, Birgit Kemmerling

**Affiliations:** 1 Center for Plant Molecular Biology (ZMBP), University of Tübingen, Tübingen, Germany; 2 Proteome Center Tübingen, Institute for Cell Biology, Tübingen, Germany; 3 Cluster of Excellence GreenRobust, University Tübingen, Tübingen, Germany; 4 Institute of Plant and Microbial Biology, Zurich-Basel Plant Science Center, University of Zürich, Zürich, Switzerland; 5 The Sainsbury Laboratory, University of East Anglia, Norwich Research Park, Norwich, UK; 6 Present Address: IRT, Paris, France; 7 Present Address: Plant Cell Biology, School of Life Sciences, Technical University of Munich, Freising, Germany

**Keywords:** Membranes & Trafficking, Plant Biology

## Abstract

Plant plasma membrane proteins are organized into distinct domains; however, the principles that govern this spatial organization are not fully understood. HYPERSENSITIVE-INDUCED REACTION (HIR) proteins are plant-specific members of the stomatin/prohibitin/ flotillin/HflK/C (SPFH) family implicated in membrane organization. Arabidopsis thaliana HIR2 interacts with multiple plasma membrane proteins, including receptor kinases such as BRASSINOSTEROID INSENSITIVE 1-ASSOCIATED KINASE (BAK1)-INTERACTING RECEPTORS 2 and 3 (BIR2 and 3) and BAK1. These interactions connect HIR2 to BAK1-mediated signaling pathways, as evidenced by impaired growth and immunity phenotypes in hir2 mutants. HIR2 is anchored to the inner leaflet of the plasma membrane through a hydrophobic domain and S-acylation. Single-particle-tracking photoactivated localization microscopy (sptPALM) revealed that HIR2 affects BAK1 mobility and clustering, exemplifying a role in regulating plasma membrane dynamics. Structural modeling with AlphaFold 3 predicts a multimeric cup-like assembly for HIR2, consistent with high molecular weight complexes identified through blue-native PAGE. These findings indicate that HIR2 contributes to the formation of membrane sub-compartments, providing a potential structural framework for spatial membrane organization that influences the dynamics and function of membrane-resident receptors.

## Synopsis

SPFH proteins are conserved organizers of membrane architecture, but how they influence signaling at the plant plasma membrane remains poorly understood. This article shows that the Arabidopsis SPFH protein HIR2 forms high-order assemblies at the plasma membrane that define membrane organization and receptor dynamics, influencing plant growth and immunity.

- HIR2 associates with plasma membrane signaling proteins, including BAK1, BIRs, RBOHD, and BIK1.
- *hir2* loss-of-function mutants display defects in both growth and immunity.
- HIR2 is targeted to the plasma membrane by its N-terminal hydrophobic region.
- HIR2 influences plasma membrane liquid order, BAK1 nanoscale mobility and its clustering in nanodomains.
- HIR2 forms high-molecular-weight multimeric assemblies consistent with a membrane-organizing role.

## Abstract (web summary)

HIR2 determines membrane order, BAK1 nanoscale behaviour and function.

## Graphical abstract

**Figure:**
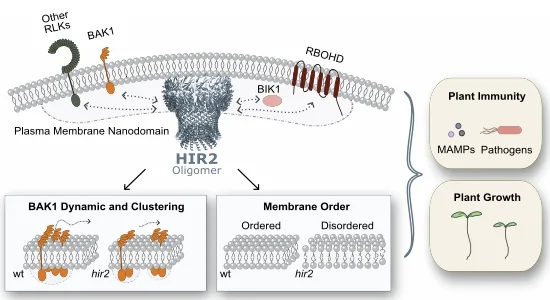

**Figure:**
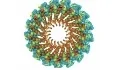

## Introduction

Membranes are dynamic fluid bilayers organized in polar membrane domains and nanodomains (Jaillais et al, 2024). The SPFH protein superfamily is represented by the name-giving stomatins, prohibitins, flotillins, HflK/C, erlins, and hypersensitive induced reaction (HIR) proteins (Di et al, 2010). In animals, SPFH-domain-containing proteins have been shown to regulate various aspects of membrane topology, regulating membrane bending and scaffolding protein complexes to modulate endocytosis and signaling (Danek et al, 2016; Rivera-Milla et al, 2006). They are enriched in detergent-resistant domains, tend to oligomerize, and are seen as nanodomain-organized proteins proposed to regulate membrane structure and function in eukaryotic systems, including plants alike (Borner et al, 2005; Martinière and Zelazny, 2021; Snyers et al, 1998). The analysis of the proposed plant plasma membrane-organizing factors flotillin and remorins indicates that they co-exist in distinct nanodomains and can contribute to membrane organization (Danek et al, 2016; Gronnier et al, 2018; Ma et al, 2021). The evolutionary conservation across various species, including bacteria, archaea, and eukaryotes, indicates the existence of SPFH domain proteins as early components of membrane-associated protein complexes (Rivera-Milla et al, 2006).

In *Arabidopsis thaliana* (hereafter Arabidopsis), the SPFH protein superfamily comprises 17 members. HIRs are plant-specific members of the SPFH protein family with four HIR isoforms encoded in the Arabidopsis ecotype Col-0 genome (Di et al, 2010; Gehl and Sweetlove, 2014; Qi et al, 2011). *HIR2* is expressed mainly in leaves, whereas *HIR1, 3*, and *4* are predominantly expressed in shoots. However, the expression is not mutually exclusive, indicating overlapping and distinct patterns and, thus, potentially distinct functions of the family members (Danek et al, 2016).

HIRs are found in detergent-resistant membrane domains and can interact with plant immune receptors such as the pattern recognition receptor FLAGELLIN SENSING 2 (FLS2)—a leucine-rich repeat receptor kinase (LRR-RK)—and with intracellular immune receptors of the nucleotide-binding-site-LRR receptors (NLRs) type, such as RESISTANCE TO *PSEUDOMONAS SYRINGAE* 2 (RPS2) (Qi et al, 2011). Multiple studies link HIRs to plant immunity and cell death (Choi et al, 2011; Jung and Hwang, 2007; Jung et al, 2008; Li et al, 2019; Liu et al, 2024; Lv et al, 2017; Qi and Katagiri, 2012; Qi et al, 2011; Qi and Katagiri, 2009; Zhou et al, 2010; Zhou et al, 2009), but a mechanistic model is still missing.

The LRR-RK BRASSINOSTEROID INSENSITIVE 1 (BRI1)-ASSOCIATED KINASE 1 (BAK1) is a common co-receptor of ligand-binding LRR-RKs (Chinchilla et al, 2009). It is involved in developmental and immune processes, e.g., by forming ligand-induced complexes with FLS2 and the brassinosteroid hormone receptor BRI1 to initiate immune and growth signaling, respectively (Chinchilla et al, 2007; Li et al, 2002; Nam and Li, 2002). BAK1-INTERACTING RECEPTORS (BIRs) are LRR-RKs with small ectodomains similar in structure to BAK1 and negative regulators of BAK1 complex formation and signaling. BIRs constitutively interact with and stabilize BAK1 and are released from BAK1 upon ligand perception (Halter et al, 2014; Hohmann et al, 2018; Imkampe et al, 2017). Cell death induced in the absence of BAK1 and BIR3 is mediated by the NLR CONSTITUTIVE SHADE AVOIDANCE 1 (CSA1) (Kemmerling et al, 2007; Schulze et al, 2022; Yang et al, 2024; Yang et al, 2022).

We identified the plant-specific protein HIR2 in the interactome of BIRs. In this study, we further demonstrate that it can interact with FLS2 complex components and other plasma membrane proteins and is genetically required for normal growth and the quantitative establishment of the immune response in Arabidopsis. Additionally, it influences the dynamics of BAK1 within the plasma membrane and membrane fluidity. This, in turn, may influence multiple signaling pathways. HIR2 plasma membrane localization depends on a non-canonical N-terminal hydrophobic stretch and S-acylation sites. The large ring structures of HIR2 predicted by AlphaFold 3, in combination with the high molecular weight complexes formed *in planta* and its plasma membrane localization, suggest that HIR2 may act as a structural platform for plasma membrane proteins. This provides a novel perspective on the mechanisms of membrane organization and compartmentalization in plants.

## Results

### HIR2 can interact with BIRs

**Figure 1:**
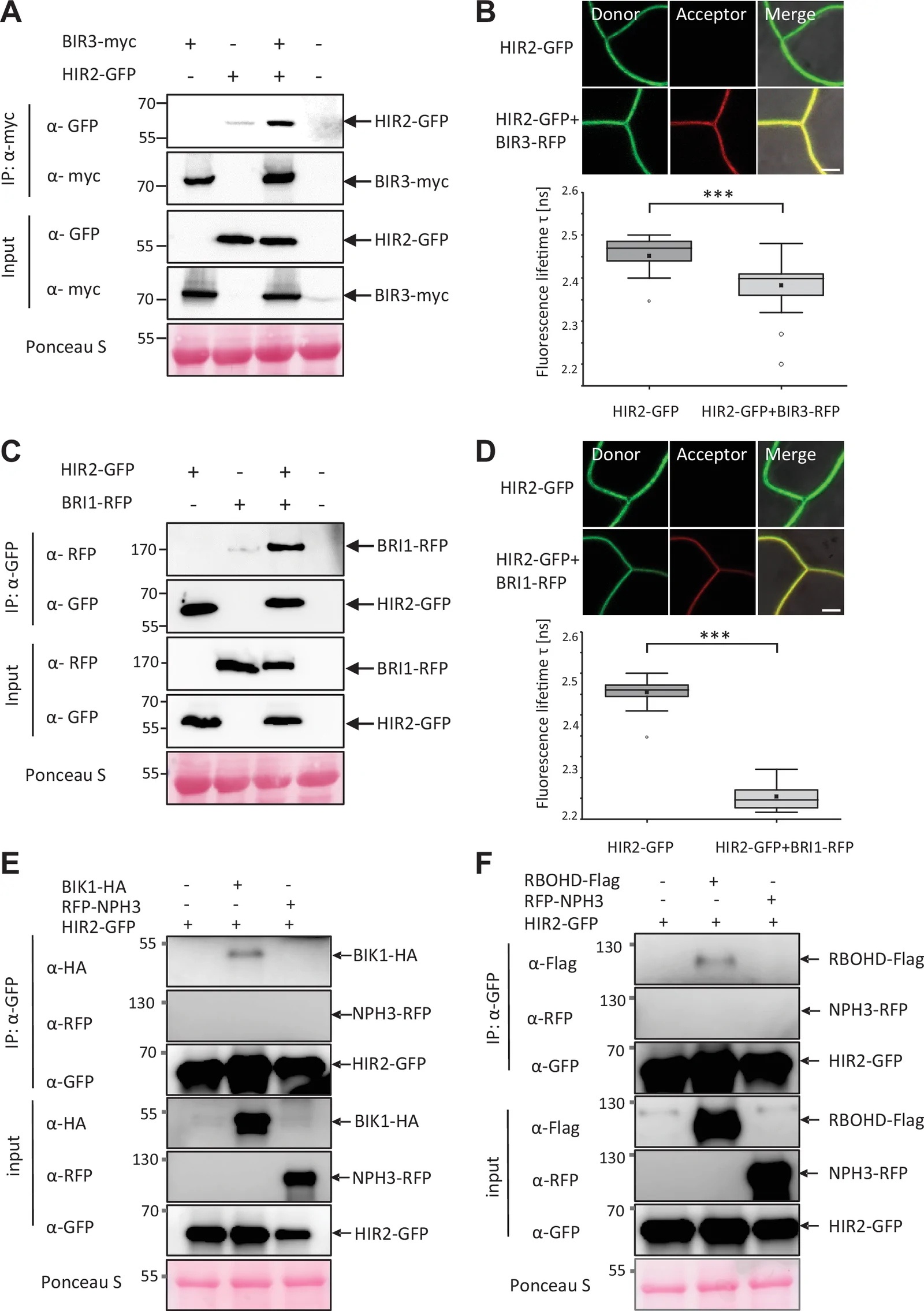
HIR2 interacts with RLKs BIR3 and BRI1, and BIK1 and RBOHD. (**A**) Co-IP of transiently expressed HIR2 in *Nicotiana benthamiana*, and BIR3 using GFP-traps were detected with α-GFP (HIR2) and α-myc (BIR3), Input shows expression of the individual proteins detected with antibodies against the respective tags. Infiltration of p19 served as a negative control. Ponceau S staining shows protein loading. (**B**) Confocal images of *Nicotiana benthamiana* leaves expressing HIR2-GFP and BIR3-RFP. HIR2-GFP acted as a donor, and the fluorescence lifetime of GFP was measured. For analysis of its lifetime, FRET-FLIM and a bi-exponential curve fitting in a defined region of interest covering the plasma membrane were performed. Donor HIR2-GFP: *n* = 36; Acceptor BIR3-RFP: *n* = 45, *P* = 1.095e-11. (**C**) Co-IP as described in (**A**) but with BRI1-RFP detected with α-RFP. (**D**) FRET-FLIM experiments as described in (**B**) but with BRI1-RFP. Donor HIR2-GFP: *n* = 16; Acceptor BRI1-RFP: *n* = 19, *P* = 1.025e-6. (**D**). (**E**, **F**) Co-IP of transiently expressed HIR2 in *Nicotiana benthamiana*, and BIK1-HA (**E**) and RBOHD (**F**) with NHP3-RFP using GFP-traps were detected with α-GFP (HIR2), α-HA (BIK1), α-RFP (NPH3), and α-Flag (RBOHD). Input shows expression of the individual proteins detected with antibodies against the respective tags. The scale bars represent 5 µm. The center line of each box indicates the median; boxes indicate the interquartile range (IQR), and whiskers represent 1.5 × IQR; outliers are shown as unfilled dots. Significant differences were determined by the Kruskal–Wallis test followed by a Steel–Dwass post-hoc correction (**P ≤ 0.01; ***P ≤ 0.001). Source data are available online for this figure.

In the interactome of BIR2 and BIR3 determined by Co-immunoprecipitation (Co-IP) and Liquid chromatography–electrospray ionization–tandem mass spectrometry (LC/ESI-MS/MS) analysis, we found tryptic peptides corresponding to the HIR2 protein (Appendix Table S1). In the BIR2 mass spectrometry experiment, we used wild-type (wt) and *BIR2* knockdown/knockout plants as controls to study the endogenous immunoprecipitated RK. In control plants lacking the BIR2 protein, the number and intensity of identified HIR2 peptides were drastically lower, indicating that the observed interaction in wt plants is specific. This shows that Arabidopsis BIR2 and HIR2 exist in complexes. We confirmed the association between HIR2 and BIR3 or BIR2 by Co-IP of transiently expressed proteins in *Nicotiana benthamiana (N.b.)* (Fig. 1A; Appendix Fig. S1A). In addition, we performed Förster resonance energy transfer-fluorescence lifetime imaging microscopy (FRET-FLIM), demonstrating the close association of HIR2 proteins with BIR3 and BIR2 (Fig. 1B; Appendix Fig. S1B).

### HIR2 can interact with multiple membrane proteins

We tested whether HIR2 could also interact with other plasma membrane proteins in further Co-IP and FRET-FLIM experiments. We analyzed BRI1 (Fig. 1C,D), BAK1 (Appendix Fig. S1C,D), and the pattern-triggered immunity (PTI) downstream components BOTRYTIS-INDUCED KINASE1 (BIK1) (Fig. 1E) and RESPIRATORY BURST OXIDASE HOMOLOG D (RBOHD) (Fig. 1F) (Kadota et al, 2014; Li et al, 2014) and demonstrated their close vicinity to HIR2 while a membrane-resident version of NON-PHOTOTROPIC HYPOCOTYL 3 (NPH3) (Reuter et al, 2021) serves as a negative control (Fig. 1E,F; Appendix Fig. S1E). For BAK1 and BIR3, we also confirmed the interaction with HIR2 in Arabidopsis protoplasts via Co-IP (Appendix Fig. S1F). Finding the interaction of HIR2 with BIR3 and BAK1 in Arabidopsis shows that interactions occur in this homologous system.

In yeast split-ubiquitin assays, we could independently show that HIR2 can directly interact with RKs such as BIRs, BAK1, and CHITIN ELICITOR RECEPTOR KINASE 1 (CERK1) (Appendix Fig. S1G). The interaction with BRI1 could not be confirmed, but split-ubiquitin assays are prone to false negative results due to steric hindrance of the tags.

We also tested other intrinsic membrane proteins, including FLS2, CERK1, CLAVATA1 (CLV1), and LOW TEMPERATURE-INDUCED 6B (Lti6b) (Appendix Fig. S1H), as well as HIR2 self-interaction and interaction with a membrane-resident NPH3^S744A^ variant (Appendix Fig. S1E). HIR2 interacted with FLS2, CERK1, CLV1, and itself, but not with NPH3. This shows that HIR2 is associated with multiple, but not all, plasma membrane proteins, indicating that HIR2 is a plasma membrane protein that can be found in proximity to various plasma membrane proteins that are not restricted to immune-related RKs. Specificity does not appear to be determined by interaction interfaces alone. Additional specificity may arise from post-translational modifications, ligand-induced complex dynamics, or the formation of hetero-oligomeric complexes.

### HIR2 loss-of-function mutants show growth and immune defects

**Figure 2:**
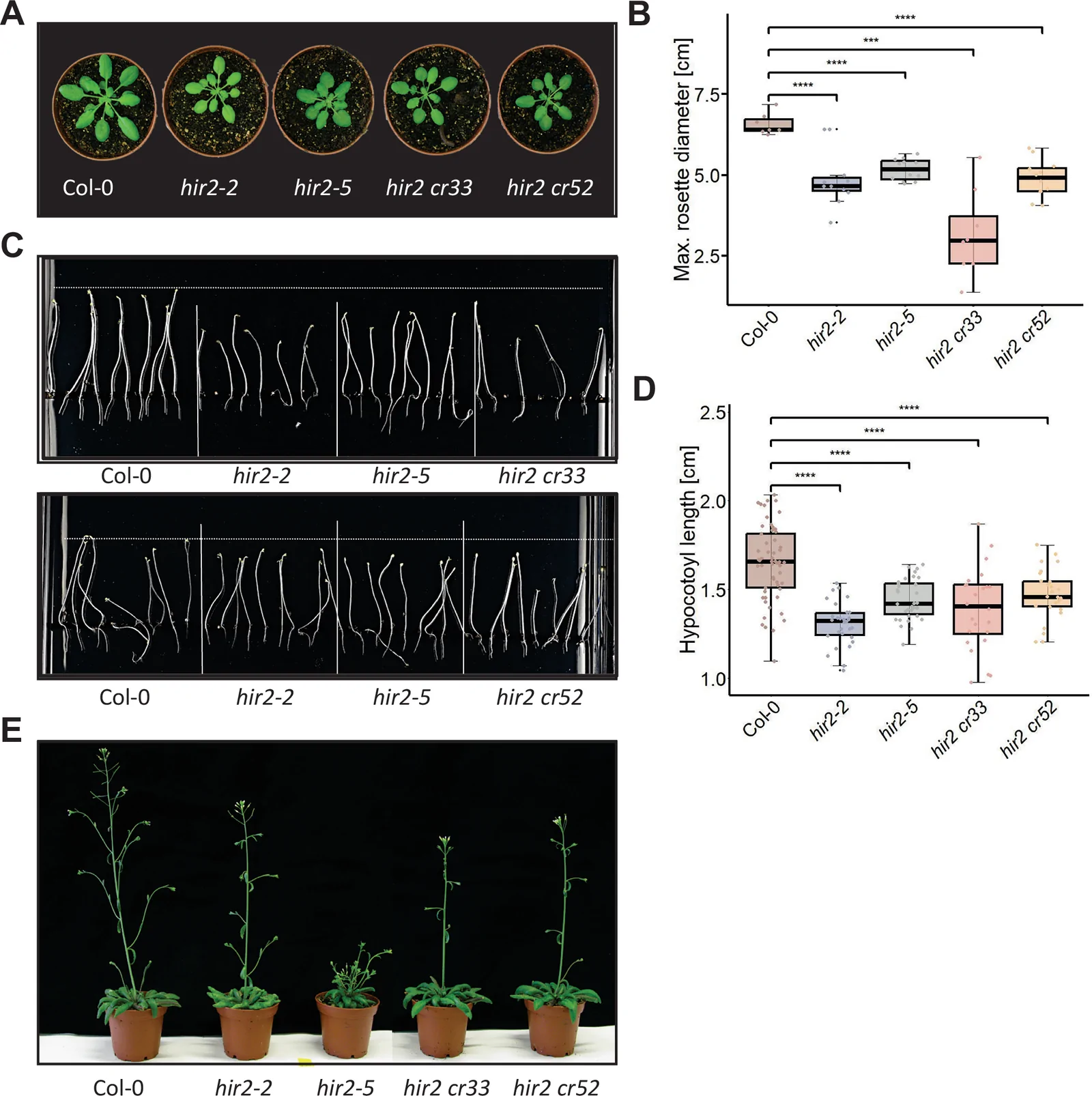
Loss of HIR2 leads to impaired Arabidopsis growth. (**A**) Representative morphology of 5-week-old Col-0, *hir2-2, hir2-5, hir2 cr33*, and* hir2 cr52* lines grown on soil. (**B**) Measurement of the mean maximum rosette diameter of 5-week-old plants of the indicated genotypes (*n* >= 8), *hir2-2* (*P* = 0.7e-4), *hir2-5* (2.5e-6), *hir2cr33* (*P* = 1.2e-4), *hir2 cr52* (*P* = 1.2e-6) (**C**, **D**) Col-0, *hir2-2* (*P* = 1.6e-14)*, hir2-5* (*P* = 1.2e-7)*, hir2 cr33* (*P* = 3.9e-5), and *hir2 cr52* (*P* = 3.9e-5) lines were grown vertically on ½ MS plates in the dark for seven days (**C**) and hypocotyl length was measured after 7 days (**D**). In the boxplot, each dot represents one seedling, boxes represent the IQR, and error bars represent the SD (*n* ≥ 25). (**E**) Representative pictures of 4-week-old plants grown in long-day conditions. All experiments were repeated at least twice with similar results. Significant differences compared to Col-0 were determined by Student’s *t* test (***P ≤ 0.001, ****P ≤ 0.0001). Source data are available online for this figure.

We identified and analyzed four T-DNA insertion *HIR2* mutants for functional experiments and observed differences in residual *HIR2* transcript levels (Appendix Fig. S2A–C). For example, the *hir2-1* and *hir2-3* mutants contained abundant residual transcripts, potentially encoding truncated but functional proteins, whereas *hir2-2* and *hir2-5* had significantly reduced transcript levels. Hence, we retained *hir2-2* and *hir2-5*. In addition, we generated CRISPR/Cas9 deletion mutants (*hir2 cr)* lacking a 773 bp segment of the *HIR2* coding sequence (Appendix Fig. S2A–C). The mutant *hir2-2, hir2-5*, and *hir2 cr* lines showed reduced hypocotyl, rosette, and inflorescence sizes (Fig. 2A–E; Appendix Fig. S2D), suggesting that HIR2 regulates plant growth.

**Figure 3:**
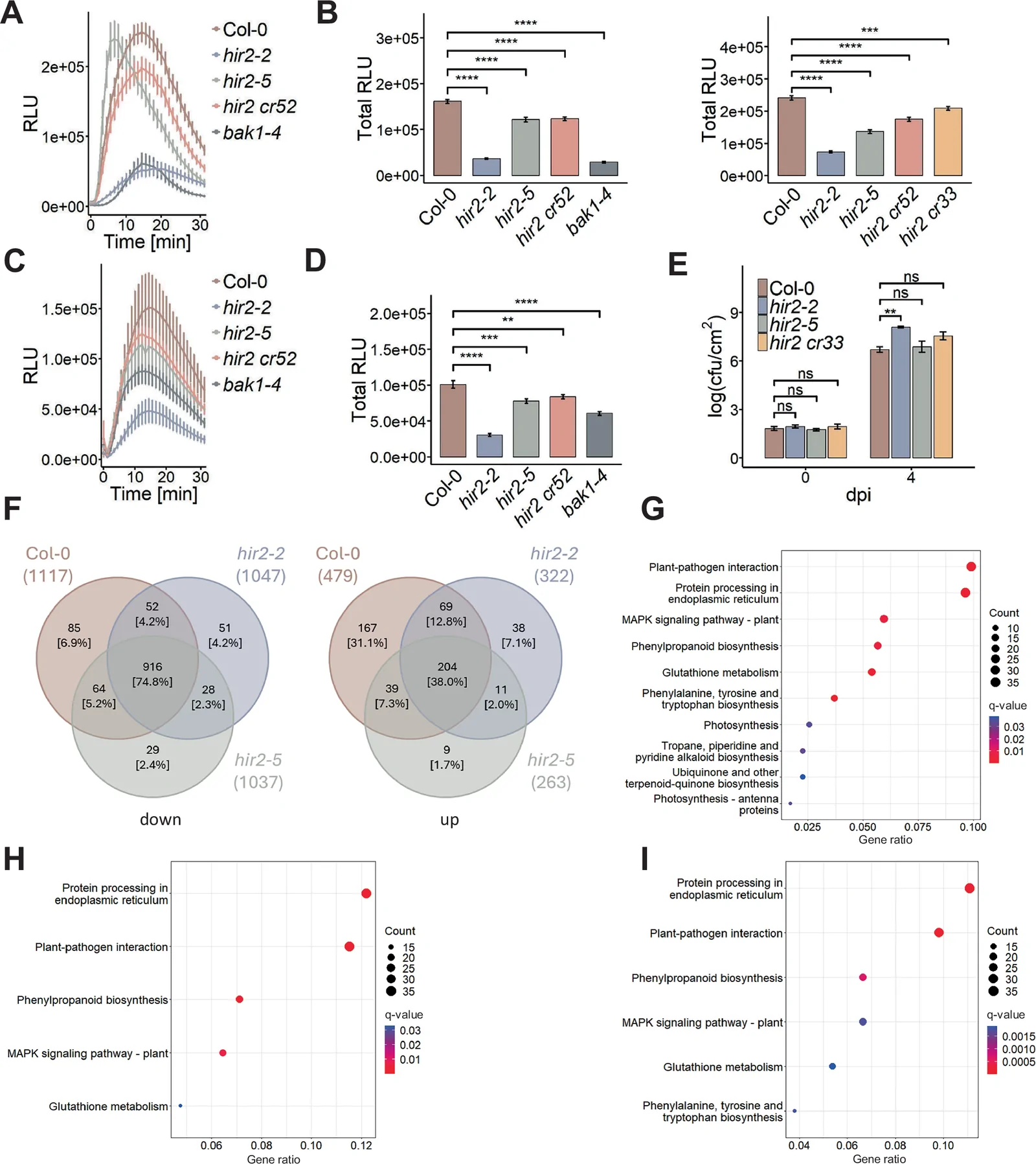
Loss of HIR2 leads to impaired MAMP and immune responses. (**A**–**D**) Reactive oxygen (ROS) production was measured as relative light units (RLU) in 5-week-old Col-0, *bak1-4, hir2-5, hir2-2*, and *hir2 cr33* and *hir2 cr52* plants induced with 1 µM flg22 (**A**, **B** left: *hir2-2* (*P* = 9.6e-93)*, hir2-5* (*P* = 2.0e-9)*, hir2 cr52* (*P* = 3.7e-10) and *bak1-4* (*P* = 6.3e-99); right: *hir2-2* (*P* = 4.5e-72)*, hir2-5* (*P* = 3.5e-26)*, hir2 cr52* (*P* = 5.9e-13) and *hir2 cr33* (*P* = 5.2e-4)) or 1 µM chitin (**C**, **D** *hir2-2* (*P* = 1.1e-27)*, hir2-5* (*P* = 3.1e-4)*, hir2 cr52* (*P* = 4.0e-3) *and bak1-4* (*P* = 6.2e-11)). In (**A**, **C**), the mean ROS production over time is shown, in (**B**, **D**) the total ROS production. The values represent means +/− SE, *n* = 12. (**E**) *Pto* DC3000 (1 × 10^4^ cfu/ml) were infiltrated in leaves of Col-0, *hir2-2* (*P* = 0.007)*, hir2-5* (*P* = 0.63)*, and hir2 cr33* (*P* = 0.31) plants. Bacterial numbers were determined at 0 and 4 days post-infection (dpi). The values represent the mean +/− SE; *n* = 12. Experiments were repeated at least twice with similar results. Significant differences compared to Col-0 were determined by Student’s *t* test ^ns^*P* > 0.05, *P ≤ 0.05, ***P ≤ 0.001, ****P ≤ 0.0001). (**F**–**I**) Transcriptomics data of 100 nM flg22 treatment of *hir2-2* and *hir2-5* mutants compared to Col-0 wild-type plants. (**F**) Venn diagram of the overlap of differentially expressed genes after flg22 treatment in the indicated genotypes made with. (**G**–**I**) GO annotation analysis of the differentially expressed genes of Col-0 (**G**), *hir2-2* (**H**), and *hir2-5* (**I**) plants. Source data are available online for this figure.

As BIRs, BAK1, and FLS2 can interact with HIR2 and are involved in PTI, we tested PTI responses in *hir2* mutant plants. The flg22-induced reactive oxygen species (ROS) burst was significantly reduced in all lines (Fig. 3A,B), indicating a role of HIR2 in PTI-induced ROS production. However, this phenotype was weaker in *hir2-5* and *hir2 cr* lines compared to *hir2-2*. Whole-genome sequencing revealed no additional T-DNA insertions in the *hir2-2* line, which would explain the stronger phenotypes. This finding suggests that low residual transcripts or other unknown factors may exert a dominant-negative effect on homologs within the HIR protein family. HIR2-overexpressing plants (Appendix Fig. S2E) showed opposite phenotypes to *hir2* mutants with enhanced ROS burst upon flg22 treatment (Appendix Fig. S3C,D). The *hir2-2* mutant, in particular, exhibits enhanced bacterial and fungal growth (Liu et al, 2024; Qi et al, 2011), suggesting a role as a positive regulator of plant immunity. The phenotypes of *hir4* mutants were enhanced when combined with *hir2* CRISPR/Cas9 alleles, supporting that weak *hir2* null mutant phenotypes are underestimated by functional redundancy with other family members (Liu et al, 2024). The CRISPR/Cas9 lines demonstrate the true null-mutant phenotypes and uncover the aberrant phenotypes of the T-DNA insertion lines.

In addition, BAK1-independent chitin-induced ROS production was also reduced in all *hir2* mutants, with *hir2-2* showing the most severe reduction (Fig. 3C,D). The impaired ROS burst of *hir2-2* lines after treatment with various microbe-associated molecular patterns (MAMPs), such as nlp20, elf18, and pep1 (Appendix Fig. S3E), underlines a general influence of HIR2 on elicitor-induced ROS production. Other MAMP-induced reactions, such as MAP-kinase (MAPK) activation or ethylene production, were not significantly altered, indicating that only the early ROS-specific branch of PTI is significantly affected by loss of HIR2 (Appendix Fig. S4A–D). The mutant phenotypes resemble those of *rbohD* mutants (Torres et al, 2002), and like *hir2* mutants, *rbohD* mutants did not show altered ethylene responses after flg22 treatment (Appendix Fig. S4E). However, the accumulation of RBOHD, the major apoplastic ROS-producing enzyme in Arabidopsis, was not affected in *hir2-2* or *hir2-5* mutants (Appendix Fig. S5), showing that the effect observed in *hir2* mutants is not due to reduced RBOHD levels.

To analyze the effects of HIR2 on disease resistance, we infected *hir2-2, hir2-5*, and *hir2 cr* mutants with *Pseudomonas syringae* pathovar tomato DC3000 (*Pto*DC3000). The average bacterial growth in *hir2-2* was up to 20-fold higher than in Col-0 (Fig. 3E). Bacterial growth was also increased in *hir2-5* and *hir2 cr33*, but not always statistically significant. Considering the partial redundancy within the HIR family, these data indicate that HIR2 contributes quantitatively to the establishment of plant immune responses (Liu et al, 2024).

Transcriptomics analysis of flg22-treated seedlings revealed that the number of significantly up- or down-regulated genes decreased (Fig. 3F) and that defense or biotic stress-related genes were reduced (Fig. 3G–I) in *hir2-2* and *hir2-5* mutants compared to Col-0. Gene ontology (GO) annotation of differentially expressed genes (DEG) showed that the number and ranking of plant-pathogen-associated genes are lower in *hir2* mutants compared to Col-0 (Fig. 3G–I).

### HIRs are relatively immobile and form nanoclusters at the plasma membrane

**Figure 4:**
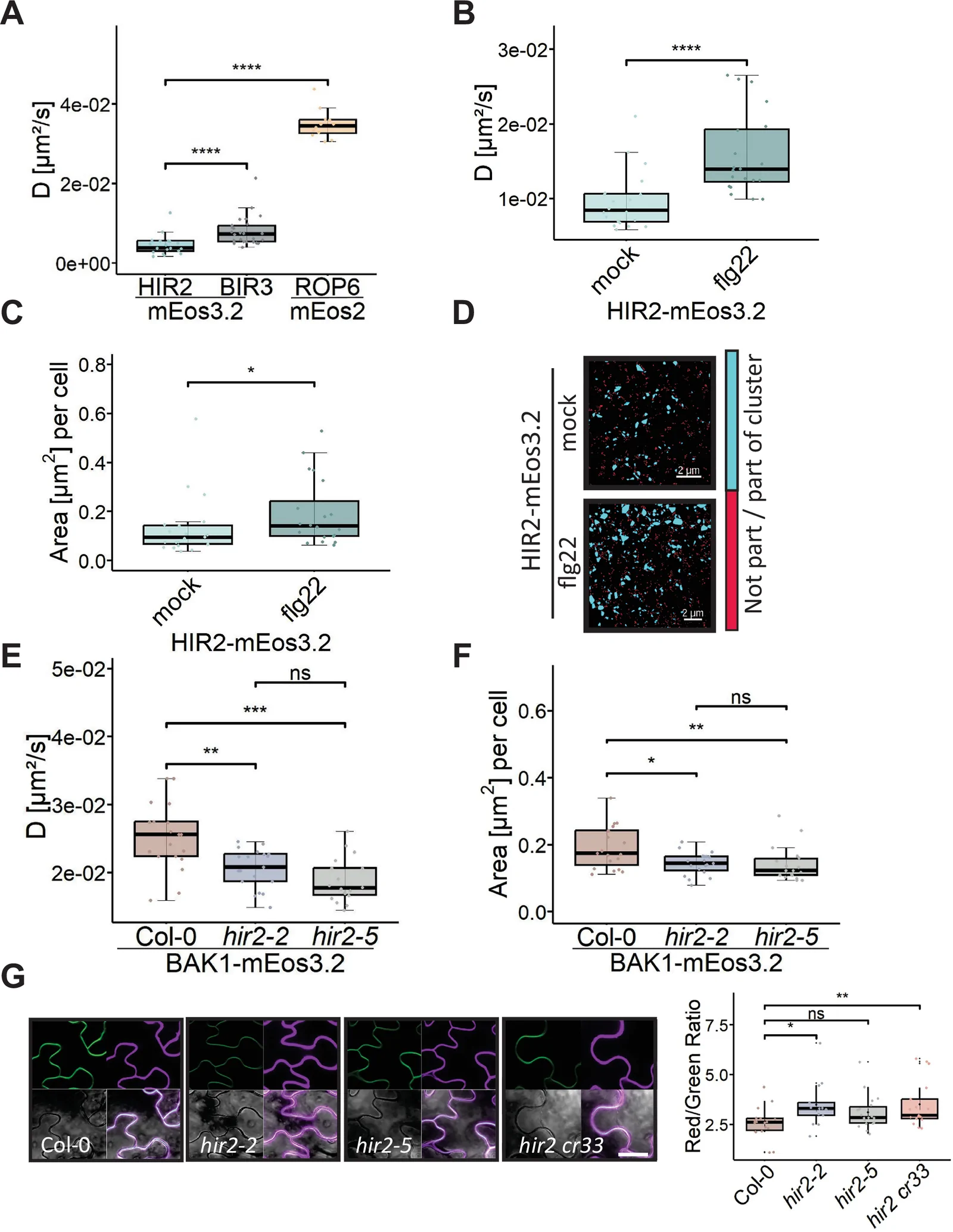
Mobility, clustering, and receptor kinase and membrane-organizing effects of HIR2. (**A**) sptPALM analysis of BIR3-mEos3.2 (*n* = 26), HIR2-mEos3.2 (*n* = 19), and ROP6-mEos2 (*n* = 12) transiently expressed in *Nicotiana benthamiana*. The mobility of the indicated proteins was determined by tracking individual mEos2 or mEos3.2-tagged molecules and calculating the peak of the individual diffusion coefficient (**D**) distribution for each sample using OneFlowTrax (p_BIR3 vs HIR2_ = 4.5e-08; p_ROP6 vs HIR2_ = 3.2e-6e-08). (**B**) sptPALM analysis of the diffusion coefficient D of HIR2-mEos3.2 with or without 100 nM flg22 (*n* = 20). Cluster analysis was performed using the nanoscale spatiotemporal indexing clustering (NASTIC) algorithm and is shown as (**C**) cluster area per cell (*n* = 20) (*P* = 0.035) and (**D**) the proportion of clustered and non-clustered HIR2 molecules. (**E**) sptPALM analysis of stable Arabidopsis Col-0, *hir2-2*, and *hir2-5* lines expressing BAK1-mEos3.2. The mobility of BAK1-mEos3.2 was determined as the peak of the diffusion coefficient for each sample (*n* = 19) (p_*hir2-2* vs Col-0_ = 0.001; p_*hir2-5* vs Col-0_ = 1.1e-4, p_*hir2-2* vs *hir2-5*_ = 0.075). (**F**) Cluster analysis of BAK1-mEos3.2 in the indicated genotypes was performed using the NASTIC algorithm and is presented as cluster area per cell. Pearson correlation analysis showed that cluster area was independent of the number of tracks per recording in datasets shown in (**C**, **F**) (*n* = 19) (p_*hir2-2* vs Col-0_ = 0.014; p_*hir2-5* vs Col-0_ = 0.004, p_*hir2-2* vs *hir2-5*_ = 0.327). (**G**, left) CLSM images of leaves of Col-0, *hir2-2, hir2-5*, and *hir2 cr33* plants infiltrated with 5 µM di-4-ANEPPDHQ dye. Fluorescence was simultaneously detected at 550–590 nm (green) and 640–670 nm (red, displayed as violet for accessibility). Lower panels show brightfield and merged images of all channels. Scale bar represents 20 µm. (**G**, right) The mean gray values of the green and red channels were measured, and their ratio (red/green) was calculated for each image (*n* ≥ 10) (p_*hir2-2* vs Col-0_ = 0.024; p_*hir2-5* vs Col-0_ = 0.052, p_Col-0 vs *hir2 cr33*_ = 0.0096). The center line of each box indicates the median; boxes indicate the interquartile range (IQR), and whiskers represent the standard error (SE). Biological replicates are shown as individual data points. The significance of differences was determined by the Wilcoxon rank-sum test (^ns^*P* > 0.05, *P ≤ 0.05, **P ≤ 0.01, ***P ≤ 0.001, ****P ≤ 0.0001). Experiments were repeated at least twice with similar results (*n* = 3). Source data are available online for this figure.

Previous studies showed that plasma membrane proteins are not uniformly distributed in the membrane but form nanoscale clusters (Gronnier et al, 2018; Jaillais and Ott, 2020). Super-resolution microscopy techniques detect and follow single fluorescently labeled molecules to determine their mobility parameters (Bayle et al, 2021; Rohr et al, 2024). These techniques revealed that membrane proteins exhibit specific dynamics (Danek et al, 2020; Gronnier et al, 2017; Gronnier et al, 2022; Lv et al, 2017). We tested the mobility and distribution of HIR2 labeled with the photoconvertible fluorophore mEos3.2 using single-particle tracking photoactivated localization microscopy (sptPALM). The diffusion coefficient was calculated using mean square displacement (MSD) analysis (Gronnier et al, 2022; Rohr et al, 2024). The mobility of HIR2 was slightly lower than that determined for BIR3 and drastically lower than for the control protein RHO OF PLANTS 6 (ROP6) (Smokvarska et al, 2020) (Fig. 4A). Our custom-built software package, OneFlowTraX, was used to analyze sptPALM data further, including analysis of the organization of HIR2 within the plasma membrane. To determine whether HIR2 displays stimulus-dependent changes in nanoscale behavior, we analyzed the effects of flg22 treatment on HIR2 dynamics and clustering. Clustering was quantified using the nanoscale spatiotemporal indexing clustering (NASTIC) algorithm in OneFlowTraX, enabling precise identification and quantification of dynamic protein clusters (Rohr et al, 2024; Wallis et al, 2023). The treatment resulted in increased mobility (Fig. 4B) as well as an expansion of HIR2 cluster size (Fig. 4C,D). These observations indicate that HIR2 undergoes dynamic nanoscale reorganization in response to immune stimulation. The increased mobility and cluster sizes may facilitate transient interactions with signaling components or promote the assembly and remodeling of membrane protein complexes during immune signaling.

### Mobility of the BAK1 co-receptor is altered in hir2 mutants

As SPFH domain proteins are proposed to be involved in membrane organization (Rivera-Milla et al, 2006), we examined whether HIR2 influences receptor organization and dynamics in the plasma membrane. We expressed BAK1-mEos3.2 fusion proteins in *hir2-2* and *hir2-5* mutant lines and compared them to lines in the Col-0 background (two independent lines each; Appendix Fig. S6). The BAK1 diffusion coefficients determined from sptPALM tracks were significantly lower in both *hir2* mutants (Fig. 4E). Two independent lines of each genotype have been analyzed with similar results. Expression levels of BAK1-mEos3.2 in the different backgrounds were comparable, and endogenous expression of BAK1 in *hir2-2* and *hir2-5* background was indistinguishable from wt (Appendix Fig. S6). Flg22 treatment reduced the mobility of BAK1-mEos constructs in Col-0. In *hir2* mutants, this flg22-induced response was still detectable; however, as basal mobility was already reduced, the additional effect of flg22 was less pronounced and therefore difficult to resolve. The overall flg22 effect on BAK1 mobility in Col-0 is relatively modest, and the impact of the *hir2* mutation on flg22-induced responses is likewise subtle. Consequently, no reliable effects could be detected, suggesting that the sensitivity of this assay is insufficient to resolve the contribution of HIR2 to flg22-induced changes in BAK1 receptor kinase mobility. Size determination of BAK1 clusters by nanoscale spatiotemporal indexing clustering (NASTIC) (Wallis et al, 2023) revealed that their size is reduced in *hir2* mutant lines (Fig. 4F). Our data indicate that loss of *HIR2* affects the membrane dynamic and organization of this RK and could thereby affect signaling and downstream responses.

### Plasma membrane order level is altered in hir2 mutants

To investigate the role of HIR2 in plasma membrane properties, we used the lipid environment–sensitive dye di-4-ANEPPDHQ, which undergoes a spectral shift depending on membrane fluidity and lipid order (Zhao et al, 2015). The dye incorporates into the plasma membrane and displays distinct emission maxima in ordered versus disordered lipid environments, emitting at ~588 nm in ordered membranes (“green”) and ~628 nm in more fluid, disordered membranes (“red”) (Jin et al, 2006). Compared to Col-0, all *hir2* mutant lines exhibited an increased red/green emission ratio (Fig. 4G), indicating an increase in membrane disorder. This increase was statistically significant in *hir2-2* and *hir2 cr33* mutants, whereas *hir2-5* displayed a weaker phenotype. Together, these results indicate that HIR2 contributes to the establishment or stabilization of ordered plasma membrane domains.

### HIR2 localizes to the plasma membrane

**Figure 5:**
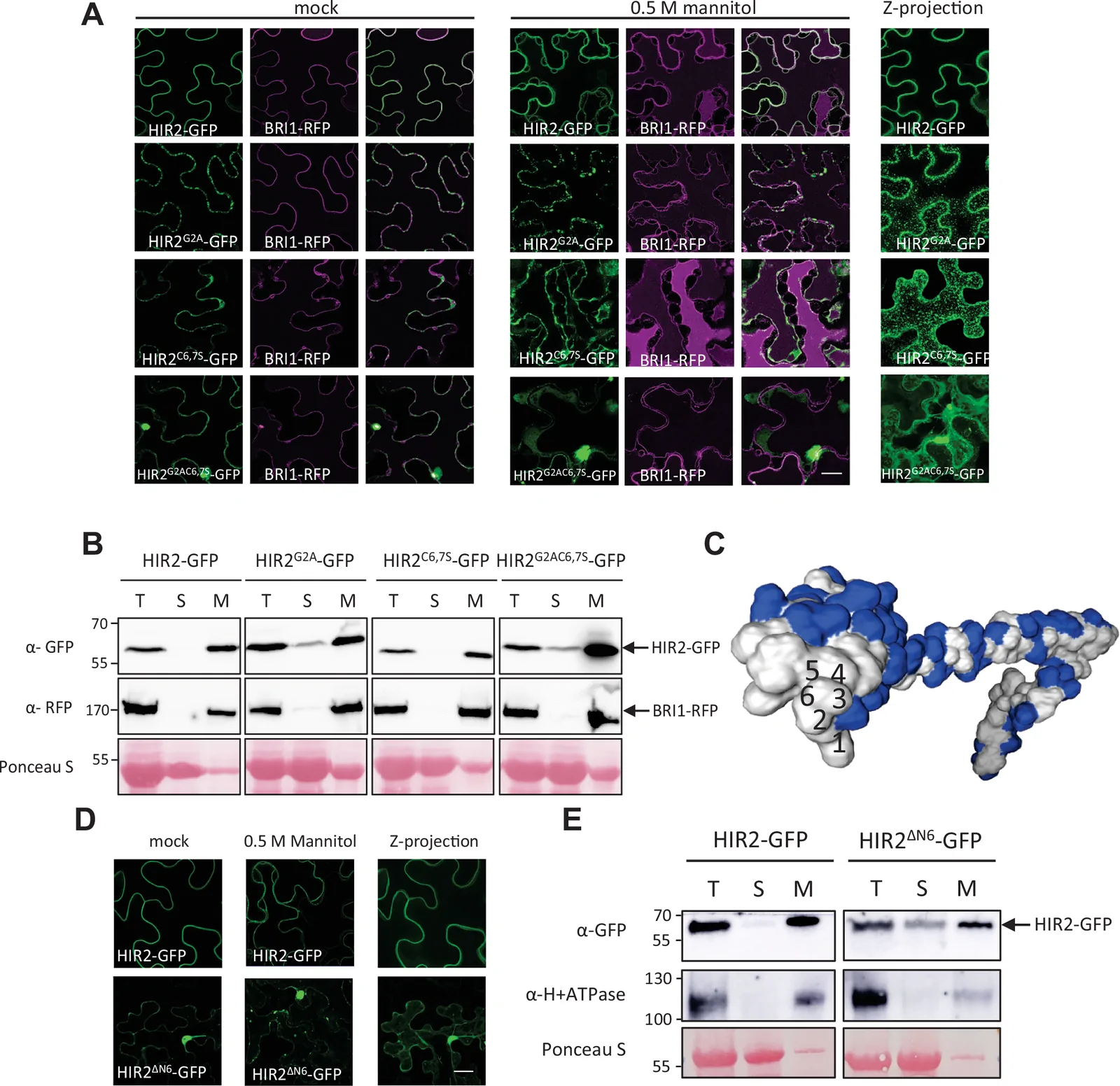
The plasma membrane localization of HIR2 is dependent on myristoylation and its hydrophobic N-terminal region. (**A**) Confocal imaging of *Nicotiana benthamiana* leaves expressing HIR2-GFP, HIR2^G2A^-GFP, HIR2^C6,7S^-GFP, or HIR2^G2AC6,7S^-GFP 48 h after Agrobacterium infiltration. As a plasma membrane localization control, BRI1-RFP was co-expressed. The leaves were treated with water (mock, left) or 0.5 M mannitol (right) for 10 min. The Z-projection presents the maximum intensity projection of Z-stacks. (**B**) Microsomal fractioning of *Nicotiana benthamiana* leaves expressing the modified HIR2 protein. Extracted proteins were separated in total (T), soluble (S), and microsomal (M) fractions and detected with α-GFP and α-RFP antibodies. Ponceau S staining shows protein loading. (**C**) Prediction of hydrophobic sites of the HIR2 protein. The modeling was performed using ColabFold (ColabFold v1.5.5: AlphaFold 2 using MMseqs2). The hydrophobic sites were predicted using FirstGlance. (**D**) Confocal imaging of *Nicotiana benthamiana* leaves expressing HIR2-GFP or HIR2^∆N6^-GFP 48 h after Agrobacterium infiltration. The leaves were treated with water (mock, left) or 0.5 M mannitol (right) for 10 min. The Z-projection presents the maximum intensity projection of Z-stacks. (**E**) Microsomal fractioning of *Nicotiana benthamiana* leaves expressing the modified HIR2 protein. Extracted proteins were separated into total (T), soluble (S), and microsomal (M) fractions and detected with α-GFP antibody. To detect a plasma membrane marker, an α-H^+^ATPase antibody was used. Ponceau S staining shows protein loading. The scale bars represent 20 µm. Experiments were repeated at least twice with similar results. Source data are available online for this figure.

HIR2 contains an SPFH domain, also known as the band7 domain, that groups it with flotillins, stomatins, prohibitins, and HflK/C, all of which are membrane-localized proteins, often facilitated by a transmembrane helix at the N-terminus. HIR proteins lack this transmembrane domain. However, transient expression of HIR2 in *N.b*. revealed a plasma membrane localization (Fig. 5A,B). We found a myristoylation site at glycine 2 and predicted palmitoylation sites at cysteine 6 and cysteine 7, which may account for this localization (Hemsley et al, 2013; Majeran et al, 2018; Morrow et al, 2002). To test their relevance, we mutated these residues to alanine or serine, respectively, and expressed them in *N.b*.. The G2A mutant still localized to the plasma membrane but displayed a spottier pattern (Fig. 5A), suggesting that myristoylation is necessary but not solely responsible for the proper membrane localization of HIR2. The C6S/C7S double mutant also showed a spotty pattern, whereas the triple G2A/C6S/C7S mutant appeared less localized to the plasma membrane (Fig. 5A). In membrane partitioning experiments, wt HIR2 and HIR2 mutants were predominantly found in the membrane fraction, indicating that disruption of these lipid modifications alone is insufficient to fully detach HIR2 from the membrane (Fig. 5B). AlphaFold 2 modeling (Jumper et al, 2021) of HIR2 combined with surface polarity analysis using FirstGlance (http://FirstGlance.jmol.org) revealed an N-terminal surface-exposed platform of HIR2 that consists of nonpolar residues, which could interact with hydrophobic parts of the (plasma) membrane (Fig. 5C). We deleted a significant part of this area up to C7 (HIR2^ΔN6^) and expressed this mutant in *N.b*.. Accumulation of HIR2^ΔN6^ was detached from the plasma membrane and visible in the cytoplasm and nucleus (Fig. 5D). Protein fractionation into soluble and membrane-localized fractions revealed that this deletion mutant is less membrane-bound and can be partially found in the soluble fraction (Fig. 5E). Some protein remained detectable in the membrane fraction, suggesting that although it is detached from the plasma membrane, it remains associated with endomembrane compartments. This indicates that the nonpolar stretch at the N-terminus of HIR2, together with S-acylation, contributes to the proper localization of HIR2 to the plasma membrane.

### Structure prediction of HIR2

**Figure 6:**
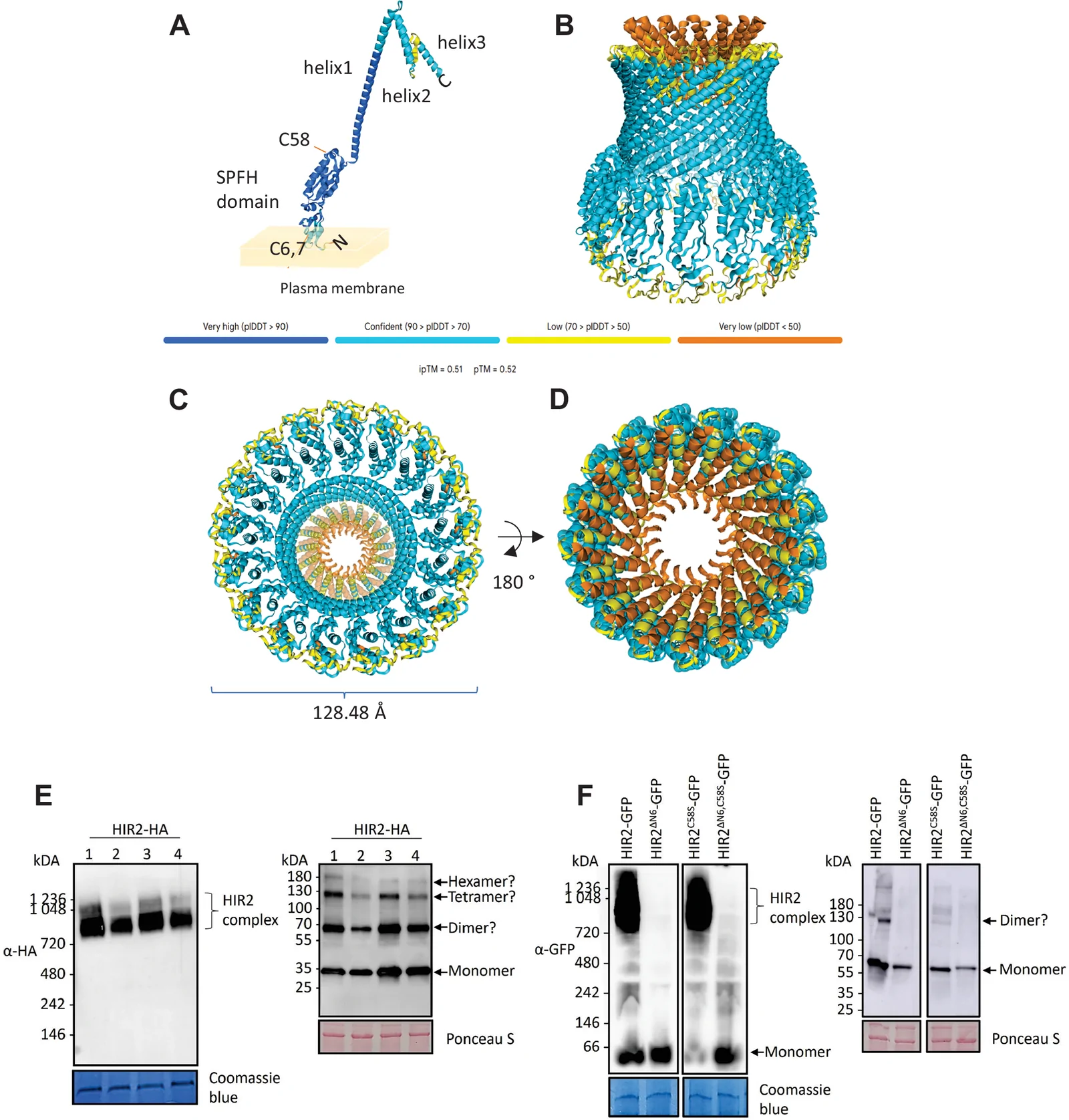
AlphaFold 3 modeling predicts high molecular weight multimeric complexes for HIR2, confirmed by blue native PAGE. (**A**) AlphaFold 3 model of a HIR2 monomer, with the orientation to the plasma membrane. (**B**) AlphaFold 3 model of a 17-mer of HIR2 from the side, (**C**) the same complex viewed from the bottom with a diameter of 128.48 Å and viewed from the top (**D**) with the bottom facing the membrane. PTM and iPTM scores indicate the accuracy of the mono- and multimer prediction, and pIDDT values the accuracy of the structural prediction. (**E**, **F**) BN-PAGE analysis of the stated HIR2 constructs expressed in *Nicotiana benthamiana* leaves. (**E**) BN-PAGE with HIR2-HA detected with anti-HA antibody, Coomassie staining shows protein loading. SDS gel (right) shows denatured protein resolution control, and Ponceau S staining shows protein loading. (**F**) BN-PAGE with HIR2-GFP, HIR2^ΔN6^-GFP, HIR2^C58S^GFP, and HIR2^ΔN6C58S^-GFP detected with anti-GFP antibody. Coomassie staining shows protein loading. SDS gel (right) shows denatured protein resolution control, and Ponceau S staining shows protein loading. Source data are available online for this figure.

AlphaFold 3 modeling of a HIR2 monomer revealed an SPFH-domain with a hydrophobic stretch at the N-terminus, a long α-helix, and two additional shorter α-helices at the C-terminus that are linked via not well-predicted and potentially flexible linkers (Fig. 6A; Appendix Fig. S7A) (Abramson et al, 2024). Modeling of a multimer (17-mer) shows a right-handed barrel-like structure, in which the individual HIR2 monomers interact at the SPFH domain and the long α−helix. The two short α−helices at the C-terminus close this barrel to a cup-like structure (Fig. 6B–D). This model is similar to recent cryo-electron microscopy (cryo-EM) structures of bacterial HflK/C or mammalian flotillin complexes (Fu and MacKinnon, 2024; Ma et al, 2022; Qiao et al, 2022; Tan et al, 2024). Confidence assessment of the AlphaFold 3 predictions showed high predicted local distance difference test (pLDDT) values between 70 and 90 for the long α-helix and parts of the SPFH domain. The two shorter α-helices were predicted with lower confidence (first helix: pLDDT 70-50; second helix: pLDDT <50), but their overall arrangement was still very similar to the confirmed structures (Fu and MacKinnon, 2024; Ma et al, 2022; Qiao et al, 2022; Tan et al, 2024). The predicted template modeling (PTM) score exceeded 0.5, suggesting the overall predicted fold is likely correct. However, the interface pTM (ipTM) score was moderate ( ~ 0.5), and therefore not in a range for a confident structure prediction. We modeled HIR2 oligomers with different stoichiometries and found that 14-mers show the highest confidence scores, including ipTM ( ~ 0.6) and pTM (0.6) (Appendix Fig. S7B). We also modeled HIR protein combinations in multimeric complexes. The modeling of hetero-multimers did not affect the ipTM values of the predicted complexes in comparison to homo-multimers. These results suggest that HIRs can likely assemble into hetero-oligomeric complexes, but the stoichiometry and composition of these complexes cannot yet be predicted and need additional evidence for an accurate evaluation.

### HIR2 forms high molecular weight complexes that assemble at the plasma membrane

To test for the stoichiometry of HIR2 complexes, we extracted total protein from *N.b* transiently expressing HA-tagged HIR2 proteins and performed blue native polyacrylamide gel electrophoresis (BN-PAGE). HIR2-HA exhibited bands at the approximate size of 800 to 1100 kDa (Fig. 6E). We confirmed this in stable Arabidopsis lines that showed higher-order complexes of the same size (Appendix Fig. S8A,B). Assuming these complexes are composed of HIR2-HA monomers, they would reflect the association of 24 to 34 HIR2 subunits, which is within the range reported for HflK/C and flotillin oligomers (Fu and MacKinnon, 2024; Ma et al, 2022; Qiao et al, 2022; Tan et al, 2024). GFP-tagged HIR2 showed bands of around 1000 kDa (Fig. 6F). Considering the larger size of the GFP tag (Appendix Fig. S8C), this would correspond to an estimated oligomer size consisting of ~17 HIR2 subunits. The lower number of estimated subunits may be due to the steric hindrance of the large tag added to the C-terminus of HIR2 or limitations in BN-PAGE resolution (Appendix Fig. S8C,D). To test whether membrane association is required for oligomerization, we analyzed the complex formation of HIR2^ΔN6^-GFP, which lacks a hydrophobic stretch required for membrane localization. This mutant failed to form high-molecular-weight complexes and was detected as HIR2-GFP monomers only (Fig. 6F). Given that the predicted oligomerization interfaces involve the SPFH domain and the long α-helix, it is unlikely that deletion of the first six N-terminal residues directly disrupts subunit interactions. Supporting this, AlphaFold 3 still models the HIR2^ΔN6^ oligomer as a ring, and Co-IP experiments with wt HIR2 still show interaction (Appendix Figs. S8E,F and S9). Therefore, the lack of HIR2^ΔN6^ oligomers is potentially due to the abolished plasma membrane localization, indicating that the formation of HIR2 oligomers occurs at the plasma membrane. In addition, we switched a cysteine at position 58 to a serine (HIR2^C58S^) to disable the formation of disulfide bonds. However, this affected neither the complex formation in BN-PAGE nor the oligomer modeling by AlphaFold 3 (Fig. 6F; Appendix Figs. S8E,G and S7).

These data show that HIR assembles into high-molecular-weight complexes in vivo, with plasma membrane association appearing to be required for complex formation. Future cryo-EM analyses will reveal the structure and composition of HIR2 complexes. The question of what cargo they hold is another topic that will be addressed in future research.

## Discussion

The findings presented here demonstrate that HIR2 is involved in plant immunity, growth, and development. HIR2 forms dynamic clusters in the plasma membrane and modifies the membrane environment while interacting with proteins at the plasma membrane. HIR2 affects receptor dynamics and nanoscale organization, correlating with membrane dynamics and signaling. The high molecular weight complexes of HIR2 are hypothesized to form a ring structure, which would represent a new type of membrane sub-compartment in plants that offers a novel perspective on the organization of membrane domains and the formation of signaling platforms in plant membranes.

Based on its specific identification in the in vivo interactomes of BIR2 and BIR3, HIR2 was selected to analyze its potential role in receptor-mediated signaling. Co-IP experiments and FRET-FLIM analyses further demonstrate the association of HIR2 with plasma membrane-localized RKs, including BAK1, BRI1, and FLS2. In addition, HIR2 interacts with key immune signaling components such as RBOHD and BIK1. Additional interactors include membrane-associated proteins, such as phospholipase D (Ho et al, 2009) and SYNTAXIN OF PLANTS 121 (SYP121) /PENETRATION 1 (PEN1) (Fujiwara et al, 2014), both implicated in defense against fungi (Collins et al, 2003). These findings imply that HIR2 is part of receptor-associated networks involved in (pattern-triggered) immunity and broader RK-mediated signaling. The capacity of HIR2 to associate with a diverse set of membrane proteins further supports its role as a scaffolding protein that contributes to the nanoscale organization of the plasma membrane. In this context, HIR2 may facilitate receptor functionality by spatially organizing signaling components.

Our data and previous studies demonstrate that HIR2 is important for efficient PTI responses (Liu et al, 2024; Qi and Katagiri, 2012). Reduced elicitor-induced ROS bursts in *hir2-2*, *hir2-5*, and *hir2 cr* mutants indicate a role for HIR2 in early immune signaling. In contrast, the absence of significant effects on other PTI markers, such as MAPK activation and ethylene production, suggests that HIR2’s role is confined to the ROS production pathway. However, redundancy within the HIR family likely masks broader functions, as MAPK signaling defects become apparent in *hir2 hir4* double mutants (Liu et al (2024)).

The reduced ROS production correlates with increased susceptibility to *Pseudomonas syringae*. Together with previous reports linking HIR proteins to resistance against bacterial, fungal, and viral pathogens, HIR2 is placed within the plant immune machinery (Choi et al, 2011; Danek et al, 2026; Jung and Hwang, 2007; Jung et al, 2008; Li et al, 2019; Liu et al, 2024; Lv et al, 2017; Mei et al, 2020; Qi and Katagiri, 2012; Qi et al, 2011; Qi and Katagiri, 2009; Zhou et al, 2010; Zhou et al, 2009). The stronger phenotypes observed in double *hir2 hir4* mutants in bacterial infection assays further support functional redundancy within the HIR family (Liu et al, 2024). Characterization of the T-DNA insertion mutants revealed that *hir2-2* reflects the multiple mutant phenotypes, indicating a dominant-negative effect in this mutant compared to weak phenotypes in *hir2* null mutants.

The reduced growth of all *hir2* mutant lines and the interaction of HIR2 with BRI1 and BAK1 suggest that HIR2 is not limited to immune signaling but also participates in more general plasma membrane-associated signaling pathways. This supports a model in which HIR2 integrates developmental and immune receptor signaling, with only partial redundancy among HIR family members.

Despite lacking a transmembrane domain, HIR2 localizes to the plasma membrane. Targeted mutational analyses indicate that myristoylation at position G2 and palmitoylation at position C6/C7 contribute to membrane association. The in vivo myristoylation of G2 and S-acylation of C6/C7 were confirmed by mass spectrometry and shift assays (Danek et al, 2026; Hemsley et al, 2013; Majeran et al, 2018; Morrow et al, 2002). However, abolishing lipid modifications alone was insufficient to impair HIR2’s association with the plasma membrane quantitatively. Instead, deletion of the first six N-terminal amino acids results in loss of plasma membrane localization, highlighting the importance of the N-terminal hydrophobic domain for plasma membrane targeting. Even though it lost its plasma membrane localization, a significant fraction of HIR2^ΔN6^ remained membrane-associated, likely reflecting accumulation in the endomembrane system. Comparable attachment mechanisms have been reported for other peripheral membrane proteins (Whited and Johs, 2015). The remorin1.3 C-terminal anchor attaches the protein to the plasma membrane by electrostatic and hydrophobic interactions (Gronnier et al, 2017; Perraki et al, 2012). The flotillin SPFH domain is sufficient to target GFP to the plasma membrane, showing that the N-terminal hydrophobic domain can mediate membrane localization without acylation or a transmembrane domain (Morrow et al, 2002). Our findings support a non-canonical membrane targeting mechanism for HIR2 based on the combined contribution of myristoylation, S-acylation, and hydrophobic interactions.

Using sptPALM, we determined the precise mobility of single HIR2 proteins. We observed that HIR2 is relatively immobile and slightly less mobile than BIR3. Previous works examining the dynamics of HIRs using fluorescence recovery after photobleaching (FRAP) and kymograms showed that HIRs are more mobile than flotillin (Danek et al, 2020; Lv et al, 2017). Moreover, we showed that flg22 treatment increases both HIR2 mobility and cluster size, resembling the behavior reported for flotillin-1 during immune activation (Yu et al, 2017). Increased mobility upon PTI activation may enhance transient encounters with immune signaling components such as FLS2, BAK1, BIK1, or RBOHD, whereas dynamic clustering could facilitate the local assembly of signaling nanodomains. The reduced mobility and cluster size of BAK1 in *hir2* mutants substantiates the idea that HIR2 influences receptor dynamics and spatial organization in the plasma membrane, correlating with quantitative effects on signaling. In parallel, membrane order measurements revealed an increase in plasma membrane disorder *hir2* mutants. This indicates that HIR2 not only influences the mobility and spatial behavior of membrane-associated proteins but is also linked to the physicochemical state of the plasma membrane. These observations further suggest that HIR2 contributes to the stabilization of ordered membrane domains rather than merely localizing within them. This interpretation is consistent with observations for REMORIN proteins such as potato REM1.3, which preferentially co-localize to ordered membrane environments and have been implicated in membrane organization (Gronnier et al, 2017; Legrand et al, 2023; Ma et al, 2021). Plasma membrane sterols and very long-chain fatty acids were recently shown to influence HIR2 organization (Danek et al, 2026). Together, these findings support a model in which HIR2 interacts with specific membrane lipids to promote the formation or stabilization of ordered plasma membrane domains. Such ordered domains may influence receptor lateral mobility, promote the assembly of signaling complexes, and spatially organize signaling processes at the plasma membrane.

Scaffold proteins have been previously shown to contribute to the stabilization of such domains. For example, in human cells, the scaffold protein MEMBRANE PALMITOYLATED PROTEIN 1 (MPP1), a member of the membrane-associated guanylate kinase (MAGUK) family, binds flotillin and promotes the formation of larger, ordered domains (Biernatowska et al, 2021; Chytla et al, 2020). The disruption of this interaction increases the membrane fluidity and impairs membrane organization, resembling the phenotypes seen in *hir2* mutants. We propose a model in which HIR2 functions as a membrane-organizing factor that contributes to the maintenance of ordered plasma membrane environments, thereby shaping the dynamic behavior of signaling proteins embedded within these domains.

Structural modeling of HIR2 by AlphaFold 3 revealed a cup-like oligomeric assembly, consistent with recent cryo-EM structures of bacterial and animal SPFH domain-containing proteins (Fu and MacKinnon, 2024; Ma et al, 2022; Qiao et al, 2022; Tan et al, 2024). BN-PAGE analysis of plant extracts showed that HIR2 forms high-molecular-weight complexes in vivo, potentially as homo-multimers or hetero-multimers with other HIR protein family members. Previous studies suggested differing oligomeric states for HIR proteins ranging from dimers to pentamers (Liu et al, 2024; Lv et al, 2017; Qi et al, 2011). Our SDS-PAGE results show bands corresponding to dimers, tetramers, and hexamers even under denaturing conditions, suggesting an assembly process seeded by highly stable dimers. Exclusively high molecular weight complexes corresponding to approximate HIR protomer numbers of 17 to 24 are detected in the BN-PAGE analyses in line with similar protomer numbers shown for other SPFH proteins (Fu and MacKinnon, 2024; Qiao et al, 2022; Tan et al, 2024). AlphaFold 3 modeling of HIR2 oligomers with increasing stoichiometry revealed a confidence peak for the 14-mer assembly. However, oligomers with higher stoichiometries displayed progressive artefactual superimposition of protomers, resulting in lower ipTM values. This, combined with our BN-PAGE data, suggests that AlphaFold 3 underestimates the stoichiometry of HIR2 oligomers. This is consistent with previous work showing that AlphaFold 3 can struggle with the modeling and stoichiometry assignment of large oligomeric assemblies (Toghani et al, 2026). The in vivo HIR2 multimer is not formed without the N-terminal six amino acid stretch, indicating that membrane association is required to form large ring structures. Co-IP analyses demonstrated that HIR2 can interact with all three other HIRs, providing many different hetero-multimer options that might confer specificity for the cargo proteins (Qi et al, 2011). Partial redundancy within the HIR family is in agreement with this model (Liu et al, 2024). Fungal and viral effectors affect the oligomeric state of HIR proteins and enhance virulence, indicating that disruption of HIR oligomeric structures benefits the pathogen (Liu et al, 2024; Mei et al, 2020). This supports the immune-modulatory role of HIR proteins and emphasizes the functional role of oligomerization for function. The predicted ring-shaped complexes may act as scaffolds for, e.g., receptor assembly, enabling efficient signal transduction.

A similar function was shown for flotillin 4 (FLOT4), an essential component of a nanodomain to which the symbiosis-related co-receptor LYSINE MOTIF KINASE 3 (LYK3) is recruited. FLOT4 functions with SymRem1 as a scaffold protein to stabilize LYK3 in the nanodomain, which is required for RK signaling (Liang et al, 2018). The ability of HIR2 to scaffold RKs, affect their spatiotemporal membrane behavior, and modulate membrane order, and likely to form separated membrane compartments, suggests that HIRs act similarly to FLOT4 as a crucial hub for efficient signal transduction from the plasma membrane.

### Conclusion

Here, we show that HIR2 interacts with various RKs, is involved in immune and growth signaling, and forms large complexes at the plasma membrane. It affects RK nanoscale behavior and membrane fluidity. Nevertheless, several questions remain to be answered. The role of the partial redundancy within the HIR family, the assembly of the ring-like structures, the precise cargo hosted by HIR2-containing complexes, and the functional significance of their nanoscale organization require further investigation.

Integrating all results, we propose that HIR2 contributes to the regulation of plasma membrane organization, thereby influencing receptor mobility and signaling output. HIR2 is incorporated into the plasma membrane and dynamically modulates its nanoscale environment. It assembles into higher-order oligomeric complexes that may contribute to the formation of distinct membrane nanodomains, which in turn could provide a structural context supporting the assembly and function of signaling platforms at the cell surface. Thus, HIR2 represents a crucial link between structural organization at the membrane and functional outcomes in plant growth and immunity, offering a unique perspective on the interplay between membrane architecture and cellular signaling.

## Methods

**Table:** Reagents and tools table

Reagent/resource	Reference or source	Identifier or catalog number
**Experimental models**		
*Nicotiana benthamiana*	Lab Stock	
*bak1-4*	NASC; (Kemmerling et al, 2007)	SALK_116202
*hir2-1*	NASC	SALK_092306
*hir2-2*	NASC	SALK_124393
*hir2-3*	NASC	SALK_095926
*hir2-5*	NASC	SAIL_1274_A05
*hir2 cr*	This work	CRISPR *hir2* line
*rbohd*	Torres et al, 2002	
*Agrobacterium tumefaciens* GV3101	Lab Stock	
*Escherichia coli* DH5a	Invitrogen	Cat: 12297-016
*Escherichia coli* Top10	Thermo Fisher Scientific	Cat: C404010
*Saccharomyces cerevisiae* THY.AP4	Lab Stock	
*Saccharomyces cerevisiae* THY.AP5	Lab Stock	
*Pseudomonas syringae* pv *tomato* DC3000	Lab stock	
**Recombinant DNA**		
pHIR2-HIR2^ΔN6^-GFP BB10	This work	
pHIR2-HIR2-GFP BB10	This work	
pHIR2-HIR2^ΔN6, C58S^-GFP BB10	This work	
35S-HIR2^C58S^-GFP pK7FWG2	This work	
Ubi1-HIR2-GFP BB10	This work	
35S-HIR2^C6,7S^-GFP pK7FWG2	Ehinger, 2022	
35S-HIR2^G2A^-GFP pK7FWG2	Ehinger, 2022	
35S-HIR2^C6,7S, G2A^-GFP pK7FWG2	Ehinger, 2022	
HIR2-pDGE347	Ehinger, 2022	
pBAK1-BAK1-mEos3.2 BB10	This work	
pHIR2-HIR2-HA BB10	This work	
pHIR2-HIR2-mEos3.2 BB10	This work	
35S-HIR2-GFP pK7FWG2	Ehinger, 2022	
35S-BAK1-RFP BB10	Ladwig et al, 2015	
35S-BIR2-RFP BB10	Schlöffel et al, 2020	
35S-BIR3-RFP BB10	Ehinger, 2022	
35S-FLS2-RFP BB10	Ehinger, 2022	
35S-BRI1-RFP BB10	Ladwig et al, 2015	
35S-CERK1-mCherry BB10	Ehinger, 2022	
35S-CLV1-mCherry BB10	Ehinger, 2022	
35S-BAK1-4xmyc BB10	Halter et al, 2014	
35S-FLS2-GFP BB10	Ehinger, 2022	
35S-BIR3-4xmyc pGWB17	Schulze et al, 2022	
35S-CERK1-HA-NLuc pCambia	This work	
HIR2-Cub-PLV	Ehinger, 2022	
BIR2-Cub-PLV	Halter et al, 2014	
BIR3-Cub-PLV	Imkampe et al, 2017	
HIR2-Nub-3xHA	Ehinger, 2022	
BAK1-Nub-3xHA	C. Grefen	
BRI1-Nub-3xHA	C. Grefen	
pNubWT	Grefen et al, 2007	
pXNubA22-Dest	Grefen et al, 2007	
**Antibodies**		
α-GFP	SICGEN	AB0020
α-BAK1	Agrisera	AS12 1858
α-RbohD	Agrisera	AS15 2962
α-RFP	Chromotek	6G6
α-cMyc	Sigma-Aldrich	C3956
α-HA	Sigma-Aldrich	H3663
α-goat IgG	Sigma-Aldrich	A5420
α-rabbit IgG	Agrisera	AS09 602
α-mouse IgG	Santa Cruz	Sc-2005
α-ATPase	Agrisera	AS07 260
α-pERK1/2	Cell Signaling	9101S
α-GFP beads	Chromotek	Gtak
**Oligonucleotides and other sequence-based reagents**		
flg22 peptide QRLSTGSRINSAKDDAAGLQIA	GenScript	RP19986
Chitin (COs DP7)	Judith Fliegmann (Nars et al, 2013)	
nlp20 peptide ATKVKAKQRGKE KVSSGRPGQHN	GenScript	Peptide Synthesis
elf18 ac-SKEKFERTKPHVNVGTIG	GenScript	Peptide Synthesis
pep1 AIMYSWYFPKDSPVTGLGHR	GenScript	Peptide Synthesis
primer	This work	Appendix Table S2
**Chemicals, enzymes, and other reagents**		
DNAse I	Thermo Fisher Scientific	EN0525
Reverse transcriptase Revert Aid	Thermo Fisher Scientific	EP0442
Phusion hot start II polymerase	Thermo Fisher Scientific	549S
dNTPs	Thermo Fisher Scientific	R0181
T4 DNA ligase	New England Biolabs	M0202S
cOmplete™-ULTRA-Mini-Tabletten, Protease-Inhibitor-Cocktail in EASYpacks	Roche	05892970001
Clarity Western ECL Substrate	Bio-Rad	1705061
L-012 Luminol	Sigma	SML2236
HRP	Sigma	P6782
DNA sequencing	Eurofins genomics	
INVIEW Transcriptome Discover + Analysis	Eurofins genomics	
RNeasy Plant Mini Kit	QIAGEN	74904
GeneJET Plasmid-Miniprep-Kit	Thermo Fisher Scientific	K0502
Trans-Blot Turbo Transfer System	Bio-Rad	1704150
**Software**		
AlphaFold 2 (ColabFold v1.5.5)	Jumper et al, 2021; Mirdita et al, 2022	https://colab.research.google.com/github/sokrypton/ColabFold/blob/main/AlphaFold2.ipynb
AlphaFold 3 (AlphaFold Server Beta)	Google DeepMind (Abramson et al, 2024)	https://alphafoldserver.com
OneFlowTraX	Rohr et al, 2024	https://github.com/svenzok/OneFlowTraX
Zenblack	Carl Zeiss Microscopy	https://www.zeiss.com/microscopy/en/products/software/zeiss-zen.html
ImageJ	Image Processing and Analysis in Java	https://imagej.net/ij/
CCTop	Stemmer et al, 2015	https://cctop.cos.uni-heidelberg.de/
ChopChop	Labun et al, 2019	https://chopchop.cbu.uib.no/
CRISPR-P 2.0	Liu et al, 2017	http://crispr.hzau.edu.cn/CRISPR2/
R	R Core Team, 2022	https://www.r-project.org/
FirstGlance		http://FirstGlance.Jmol.Org
readxl 1.4.3	Wickham, 2023a	https://CRAN.R-project.org/package=readxl
ggpubr 0.6.0	Kassambara, 2026	https://rpkgs.datanovia.com/ggpubr/
ggplot2 3.5.1	Wickham, 2016	https://ggplot2.tidyverse.org/
tidyr 1.3.1	Wickham and Girlich, 2024	https://tidyr.tidyverse.org/
dplyr 1.1.4	Wickham et al, 2023b	https://dplyr.tidyverse.org/
ghibli 0.3.4	Henderson, 2022	https://cran.r-project.org/web/packages/ghibli/index.html
biomaRt	Durinck et al, 2005; Durinck et al, 2009	https://bioconductor.org/packages/release/bioc/html/biomaRt.html
clusterProfiler 4.10.0	Wu et al, 2023	https://bioconductor.org/packages/release/bioc/html/clusterProfiler.html
enrichplot 1.22.0	Yu, 2026	https://bioconductor.org/packages/release/bioc/html/enrichplot.html
org.At.tair.db 3.18.0	Carlson, 2022	https://bioconductor.org/packages/release/data/annotation/html/org.At.tair.db.html
agriGO v2.0	Tian et al, 2017	http://systemsbiology.cau.edu.cn/agriGOv2/
KEGG	Kanehisa et al, 2024	https://www.genome.jp/kegg/

### Plant material and growth conditions

*Arabidopsis thaliana* plants were grown for 5–6 weeks on soil in growth chambers (8 h light, 16 h dark, 22 °C, 100 mEm^−2^ s^−1^) or on ½ MS medium.

The T-DNA insertion mutants used were *hir2-2* (SALK_124393), *hir2-5 (SAIL_1274_A05), bak1-4*, and *rbohd*. Stable transgenic lines were obtained by floral dipping (Clough and Bent, 1998). Successfully transformed seeds were selected by fluorescence stereomicroscope (Zeiss, SteREO Discovery.V8) if they expressed a FAST marker (Shimada et al, 2010), or by BASTA selection.

### Plasmid construction

Primers used in this study are shown in Appendix Table S2.

### GoldenGate cloning

Two different cloning strategies were used to express the gene of interest in this work. Expression clones with BB10 as the final vector were constructed using the GoldenGate technique (Binder et al, 2014). Depending on the clone, native, 35S, or Ubi-1 promoters were used as A-B modules. The coding sequences of the GOIs were used as B-D modules. Fluorophores were designed as D-E modules, resulting in a C-terminal fusion. All constructs were tested by Sanger sequencing, and the protein expression was determined by immunoblotting and/or confocal laser scanning microscopy.

### Gateway cloning

Expression clones with pK7FWG2 as the final vector were constructed using the Gateway cloning technology (Thermo Scientific) according to the manufacturer’s protocol. Entry clones were generated using the pENTR/D-TOPO or the pCR8/GW/TOPO cloning kit. To attach A-overhangs, 7.9 µl of PCR amplicons, 1 µl 10 mM dATP, 1 µl 10x Taq-buffer, and 0.1 µl Taq polymerase were incubated for 10 min at 72 °C. The TOPO reaction was transformed into *E. coli* cells. To transfer the gene of interest into an expression vector, LR reaction was performed using the Gateway LR Clonase II Enzyme Mix (Thermo Scientific).

### Cloning of CRISPR/Cas9 constructs

CRISPR/Cas9 constructs were cloned according to (Stuttmann et al, 2021). Target sites were chosen using three online tools: CCTop (Stemmer et al, 2015), ChopChop (Labun et al, 2019), and CRISPR-P 2.0 (Liu et al, 2017). Two single guide RNAs (sgRNA) located within the coding sequence of HIR2 were used (sgRNA1 in exon 1, position 264, and sgRNA2 in exon 4, position 1286).

### Hypocotyl measurements

For the hypocotyl measurements, plants were grown on ½ MS plates for 6 h in the light and 7 days in the dark. Pictures were taken, and the length of the hypocotyl was measured with ImageJ and its measuring tools (Schneider et al, 2012).

### ROS burst measurements

Leaf discs were punched out with a 5-mm biopsy stencil (KAI Biopsy Punch) and incubated overnight in water. The next day, they were transferred to a white 96-well plate containing 80 µl water. Next, 10 µl of luminol solution (20 µM luminol L-012, 10 µg/ml horseradish peroxidase) was added, and the baseline of the ROS burst was determined, followed by treatment with the respective elicitor. The samples were measured with a multi-plate reader (Centro LB 900, Berthold Technologies).

### MAPK phosphorylation assay

The phosphorylation of MAPKs was analyzed according to (Willmann et al, 2014). In brief, 100 mg plant tissue was homogenized, and 100 µl protein extraction buffer (50 mM Tris–HCl, pH 7.5, 5 mM EDTA, 5 mM EGTA, 2 mM DTT, 100 mM ß-glycerophosphate, 10 mM sodium orthovanadate (Na_3_VO_4_), 10 mM sodium fluoride (NaF), cOmplete™-ULTRA-Mini-Tablet (Roche), and PhosSTOP™ (Roche)) was added. Samples were centrifuged at 15,000× *g* for 20 min at 4 °C. The supernatant was transferred to a fresh tube, and the protein concentration was determined using Bradford solution (Bio-Rad). An equal amount of protein from each sample was then used for SDS-PAGE and immunoblotting assays.

### Pseudomonas infection

*Pseudomonas syringae* pv*. tomato* DC3000 at 10^4^ cfu/ml concentration was infiltrated into the leaves of 5- to 6-week-old plants. Leaf discs were harvested at the indicated time, homogenized in 10 mM MgCl_2_, diluted, and plated on LB plates with the appropriate antibiotics. Colonies were counted and converted to CFU/cm² leaf area.

### Ethylene production

Four-leaf discs were punched out with a 5 mm biopsy stencil (KAI Biopsy Punch) and incubated in water overnight. The next day, they were transferred to a glass vial containing 400 µl water (Milli-Q®). For induction, 5 µl water (control) or elicitor was added, and the vials were sealed with rubber caps and gently shaken for 4 h. The ethylene produced was measured by gas chromatography as described by (Felix et al, 1999).

### Transcriptomics

For each sample, seven seedlings grown for 10 days on ½ MS were treated with either water (mock) or 1 µM flg22 for 10 min and then frozen in liquid N_2_. The RNA was extracted using the RNeasy Plant Kit (Qiagen) and diluted to a concentration of 150 ng. The RNA quality was checked with a NanoDrop (Thermo Fisher) and its integrity with Bioanalyzer (Agilent RNA 6000 Pico). RNA sequencing was performed by Eurofins (NovaSeq 6000 S4 PE150 XP), and bioinformatic analysis was performed. Further analyses, such as overrepresentation analysis, were performed using AgriGO v2.0 (Tian et al, 2017), KEGG (Kanehisa et al, 2024), and R with the packages listed in the Resources and Tools table.

### Transient expression in *Nicotiana benthamiana*

*Agrobacterium tumefaciens* GV3101 containing the respective constructs was grown for 24 h at 28 °C in LB medium supplemented with appropriate antibiotics. The next day, fresh LB ( + antibiotics) was inoculated with the overnight culture and incubated for 24 h at 28 °C. The cultures were pelleted, resuspended in 10 mM MgCl_2_ to an OD_600_ of 1, supplemented with 100 µM acetosyringone, and incubated in the dark for 3 h. Agrobacteria with different constructs were, if required, mixed 1:1 and infiltrated into 3-week-old *N.b*. leaves. The leaves were harvested 2 to 3 days after infiltration.

### SDS-PAGE and immunoblotting

Proteins were separated using either 7.5% or 4–15% Mini-PROTEAN® TGX™ gels (Bio-Rad) and blotted on nitrocellulose membranes with the Trans-Blot Turbo Transfer System 7-min protocol (Bio-Rad). The membranes were blocked in 5% milk in TBS-T for 60 min and incubated overnight with the first antibody at 4 °C on an orbital shaker. The next day, the membranes were washed 3× with TBS-T (5–10 min each), and the second antibody was added for 1 h at RT on an orbital shaker. The membranes were washed 3× with TBS-T, and the proteins were detected with ECL substrate (Bio-Rad). The used antibodies are listed in the Resources and Tools table.

### Protoplast extraction and transfection

Transient expression in leaf mesophyll protoplasts of Col-0 plants was performed using a polyethylene glycol transformation method as described previously (Wu et al, 2009). For individual transfections, aliquots with 80,000 protoplasts were co-transfected with 100 µg of plasmid DNA encoding BAK1-GFP, BIR3-GFP, and HIR2-HA. Free GFP was used as a negative control. The protoplasts were resuspended in 4 mL of W5 solution (2 mM MES, 154 mM NaCl, 125 mM CaCl_2_, 5 mM KCl; pH 5.7) and aliquoted into a 24-well plate. After 16 h of incubation at RT in darkness, total protein was extracted and used for Co-IP assays.

### Co-immunoprecipitations (Co-IP)

Two hundred milligrams of leaf material or 4 × 10^5^ protoplast cells were flash frozen in liquid N_2_, homogenized with stainless steel beads in a benchtop homogenizer (TissueLyser LT, QIAGEN), and 800 µl extraction buffer (10% glycerol, 150 mM Tris-HCL pH 7.5, 1 mM EDTA,150 mM NaCl, 0.01 M DTT, 1% Nonidet, cOmplete™-ULTRA-Mini-Tablet (Roche), 0.02 g/ml PVPP) was added. Samples were incubated for 1 h at 4 °C on an overhead shaker and purified by 2× centrifugation at 14,000 rpm at 4 °C for 5 min. For each sample, 30 µl GFP-TRAP® Agarose beads (ChromoTek) were washed three times with GTEN Mix buffer (10% glycerol, 150 mM Tris-HCL pH 7.5, 1 mM EDTA, 150 mM NaCl, 0.01 M DTT, 1% Nonidet) and stored in 70 µl GTEN Mix buffer. Sixty microliters of the freshly washed beads were transferred to the protein extract and incubated at 4 °C for 1 h with inversion. The protein-bead mixture was centrifuged two times at 2500× *g* for 2 min at 4 °C and washed two times with buffer A (50 mM Tris, pH 7.5, 150 mM NaCl) and two times with buffer B (50 mM Tris, pH 7.5, 50 mM NaCl). The proteins were removed from the beads with 5× SDS sample buffer and boiled at 95 °C for 5 min.

### Mating-based split-ubiquitin system (mbSUS)

For the analysis of direct protein-protein interactions, the mbSUS was performed as described by Grefen et al (2007, 2009).

### Blue native polyacrylamide gel electrophoresis (BN-PAGE)

Each step was performed on ice in a cold room (4 °C) to avoid protein degradation. An aliquot of 100 mg fresh leaf material was frozen in liquid N_2_ and homogenized with stainless steel beads in a benchtop homogenizer. Next, 600 µl of buffer (150 µl 4x Native PAGE^TM^ Sample buffer (ThermoFisher), 60 µl 10% DDM, 390 µl H_2_O) was added, and the samples were incubated for 1 h at 4 °C on an overhead shaker. After 30 min of centrifugation at 14,000 rpm, the supernatant was split into two fresh reaction tubes. For the SDS samples, 5x SDS loading buffer was added and boiled for 5 min at 95 °C. For the native samples, 5% NativePAGE^TM^ G-250 Sample Additive (ThermoFisher) was added. The denatured samples were run according to the described SDS-PAGE method. Native samples were run on NativePAGE™ 3–12% Bis-Tris Gels (ThermoFisher) in light running buffer (50 ml 20x NativePAGE™ Running Buffer (ThermoFisher), 5 ml NativePAGE™ Cathode buffer additive (Thermo Fisher), 954 ml Water) for 30 min at 150 V, followed by 90 min at 250 V. After the run, the proteins were blotted onto a PVDF membrane for 30 min at a constant 20 V using the Trans-Blot Turbo Transfer System (Bio-Rad). The membrane was incubated in 8% acetic acid for 15 min, rinsed with water twice, and dried on filter paper. Ethanol was added to remove the dye, and all visible marker bands were labeled. The ethanol was removed, and the membrane was washed three times in TBS-T for 2 min each time. The immunoblotting took place as described previously.

### Microsomal fractioning

Two hundred milligrams of leaf material were frozen in liquid N_2_ and homogenized with stainless beads in a benchtop homogenizer. Next, 210 µl microliters of lysis buffer (0.33 M sucrose, 20 mM Tris/HCl pH 7.5, 1 mM EDTA, 10 mM DTT, cOmplete™-ULTRA-Mini-Tablet (Roche)) was added, homogenized by brief vortexing, and kept on ice for 15 min. After spinning at 5000× *g* for 10 min, the supernatant was transferred to a fresh reaction tube. This procedure was repeated once more. An aliquot was taken for the total protein fraction. The remainder was centrifuged at 20,000×* g* for 1 h at 4 °C. An aliquot of the supernatant was taken for the soluble protein fraction, and the remaining supernatant was removed. Finally, the pellet was dissolved in 60 µl lysis buffer for the microsomal protein fraction.

### Confocal laser scanning microscopy

Confocal laser scanning microscopy was performed with the LSM880 (Zeiss). Images were acquired with a 63×/1.2 NA or 40×/1.2 NA water-immersion objective. GFP was excited at 488 nm and detected in the spectral range of 500–550 nm; RFP was excited at 561 nm and detected between 600 and 650 nm.

For Förster Resonance Energy Transfer-Fluorescence Lifetime Imaging Microscopy (FRET-FLIM) analysis, donor and acceptor proteins were transiently expressed in *N. benthamiana* and imaged with the SP8 (Leica) in combination with the SymPhoTime 64 software (PicoQuant) as described previously (Pruitt et al, 2021). The average GFP fluorescence lifetime τ [ns] was obtained by bi-exponential curve fitting in a defined region of interest covering the plasma membrane. As cell death is shown to correlate with high amplitude A [2] values, data were only included when the A [1] [kCnts] to A [2] [kCnts] ratio was above 1.5.

For the localization analysis, leaf pieces were placed in 0.5 M mannitol solution or H_2_O (mock), and the images were processed with the ZEN Black (Zeiss) software.

### sptPALM acquisition and analysis

A custom-built microscope setup and software were used for the acquisition of sptPALM data and their analysis (Rohr et al, 2024). Either samples of 6-week-old *Nicotiana benthamiana* leaves grown on soil or 7- to 10-day old *Arabidopsis thaliana* seedlings grown on ½ MS plates were imaged. To avoid damage to the cells due to prolonged laser irradiation, a sample was irradiated for a maximum of 15 min. Approximately 7–8 cells were analyzed per sample. The image stacks were analyzed using the OneFlowTraX software (Rohr et al, 2024), with cluster analyses based on NASTIC algorithms. Identical localization parameters (conversion factor (e/ADU) = 0.48, offset (ADU) = 100, pixel size (nm) = 100, filter size (px) = 1.2, cut-off (photons) = 3, PSF ROI size (px) = 9), tracking parameters (max. linking distance (nm) = 150, max. gap closing (frames) = 4) and cluster parameters (average cluster size per cell, at least 400 tracks per cell, 5 tracks per cluster, radius factor = 1 for NASTIC) were used for each measurement.

### Staining with di‑4‑ANEPPDHQ

Leaves from adult *Arabidopsis* plants were syringe-infiltrated with freshly prepared 5 µM di-4-ANEPPDHQ in water. After 5 min, leaf discs were collected and imaged using a ZEISS LSM880 confocal microscope with a ×40 water-immersion objective. Fluorescence excitation was performed at 488 nm, and emission was detected simultaneously in two separate channels: 550–590 nm (green) and 640–670 nm (red). For quantitative assessment of membrane fluorescence intensity, binary masks were generated using the Iterative Self-Organizing Data Analysis Technique algorithm (ISODATA) (Ball and Hall, 1965). The ratio of mean fluorescence intensities between the red and green channels (red/green) was calculated for each masked region. Data analysis was performed using Python packages.

### LC-MS/MS

Two grams of fresh leaf material were used, and immunoprecipitation was performed as described above, but with higher volumes of extraction buffer (5 ml) and GFP-agarose beads (60 µl). Proteins were purified and fractionated by SDS-PAGE, followed by in-gel digestion with trypsin. LC-MS/MS analysis was performed using an Easy nanoHPLC system (Proxeon Biosystems) coupled to either an LTQ Orbitrap XL or a QExactive HF (both Thermo Fisher Scientific) as described previously (Bakula et al, 2017) using 57 min segmented gradients. MS raw data were processed using the MaxQuant software, either version 1.0.14.3 or version 1.6.7.0 (latter with an integrated Andromeda search engine) as described elsewhere (Franz-Wachtel et al, 2012). The spectra were matched with a reference proteome of *Arabidopsis thaliana* (UP000006548). N-terminal acetylation and oxidation of methionine were set as variable modifications, whereby carbamidomethylation on cysteine was defined as a fixed modification. Data were processed with a false discovery rate (FDR) setting of 1%. Some of the data were previously published and are available in (Halter, 2017) and (Schulze et al, 2022) (PRIDE: PXD036942).

### AlphaFold modeling

The structure predictions were generated using AlphaFold 2 (https://colab.research.google.com/github/sokrypton/ColabFold/blob/main/AlphaFold2.ipynb; (Jumper et al, 2021; Mirdita et al, 2022)) and AlphaFold 3 (https://alphafoldserver.com/ (Abramson et al, 2024)). FASTA sequences of HIR2 were retrieved from Aramemnon and introduced into the Web-based AlphaFold server. Homo and hetero-oligomeric complexes with HIR1, 3, and 4 were modeled. Seed variations were tested to determine the reliability of the modeling. The best of five models was used for further analysis of plDDT confidence scores, and pTM and ipTM values were used for reliability evaluation of the intra- and intermolecular interactions, respectively. The predicted aligned error (PAE) was checked for the mono and oligomeric predictions. Screening across different oligomeric states based on ipTM scores identified the highest-confidence models for assemblies comprising 14 subunits. Predictions corresponding to larger oligomeric assemblies frequently displayed steric clashes, which are most likely attributable to limitations and artifacts of the modeling approach at high stoichiometries rather than excluding the existence of large assemblies in vivo (Appendix Fig. S7B).

### Statistical analysis

Statistical analyses were performed using R. Statistical significance between two normally distributed samples was assessed using Student’s *t* test with Holm correction if multiple samples were tested in parallel, and, between non-normally distributed samples using the Wilcoxon rank-sum test. Between normally distributed groups, statistical significance was analyzed using a one-way ANOVA combined with Tukey’s Honest Significant Difference (HSD) test, and for non-normally distributed groups, the Kruskal–Wallis test. Statistically significant differences were indicated by asterisks. For FRET-FLIM analyses, a Kruskal–Wallis test followed by a Steel–Dwass post-hoc correction was used.

## Supplementary information


Appendix
Peer Review File
Source data Fig. 1
Source data Fig. 2
Source data Fig. 3
Source data Fig. 4
Source data Fig. 5
Source data Fig. 6


## Data Availability

A subset of the mass spectrometry data has been reported previously and is available through Halter (2017) and Schulze et al (2022), and at Protein interaction IP-MS data: PRIDE PXD036942. Microscopy images: BioImage Archive BIA https://doi.org/10.6019/S-BIAD3572 (https://www.ebi.ac.uk/biostudies/bioimages/studies/S-BIAD3572). RNA-seq data: Gene Expression Omnibus GEO GSE334721. The source data of this paper are collected in the following database record: biostudies:S-SCDT-10_1038-S44318-026-00877-y.
