# Supplementary material for: The Arabidopsis SPFH protein HIR2 modulates receptor signaling and plasma membrane organization
Source: EMBO J. 2026 Jul 29;45(17):6176–96. doi: 10.1038/s44318-026-00877-y (PMC13534476; doi:10.1038/s44318-026-00877-y)
Supplement: Supplementary file 1 [file 44318_2026_877_MOESM1_ESM.pdf]

Appendix for
“The Arabidopsis SPFH protein HIR2 modulates receptor signaling and plasma membrane organization”

Appendix Table S1: Mass spectrometry detects HIRs interacting with BIR2 and BIR3……………p1

Appendix Figure S1: HIR2 interacts with several plasma membrane receptors/proteins. (related to Figure1)……p2

Appendix Figure S2: *hir2* mutant characterization. (related to Figure 2 and 3)…………………..……p4

Appendix Figure S3: Mutant and overexpressor phenotypes after various MAMP treatments. (related to Figure 3)……p5

Appendix Figure S4: Loss of *HIR2* does not impact MAP kinase phosphorylation and ethylene production. (related to Figure 3)……………………………………………………………………………………….p6

Appendix Figure S5: Expression of RbohD is not impaired in the *hir2* mutants. (related to Figure 3)………p7

Appendix Figure S6: Expression control of BAK1-mEos3.2 in Col-0, *hir2-2*, and *hir2-5* background. (related to Figure 4)………………………………………………………………………………………….p8

Appendix Figure S7 : SPFH domain prediction in AlphaFold modelled HIR2 and protomer screening of HIR2 oligomers……………………………………………………………………………………………..p9

Appendix Figure S8: AlphaFold modeling of tagged and mutated HIR2 versions. (related to Figure 6)………..p10

Appendix Figure S9 : Self-interaction of HIR2 and HIR2 mutants ……………………………………..p11

Appendix Table S2: Primers used in this study (related to Methods)……………………………….p12

Appendix Table S1: Mass spectrometry detects HIRs interacting with BIR2 and BIR3.

Co-IP of endogenous BIR2 and BIR3-GFP protein with α-BIR2-specific antibody and GFP traps, respectively.
Identification of the interacting HIR proteins by mass spectrometry.

exp 349 R1-R4	BIR2 IP		Col-0	Ws	*amiBir2*	*bir2-2*					
Protein IDs	Protein Descriptions	Gene names TAIR/AGI	Peptides (seq) R01	Peptides (seq) R02	Peptides (seq) R03	Peptides (seq) R04	Intensity	Intensity R01	Intensity R02	Intensity R03	Intensity R04
Q9CAR7	>Q9CAR7 sp|HIR2_ARATH Hypersensitive-induced response protein 2 OS=Arabidopsis thaliana GN=HIR2 PE=1 SV=1	HIR1	5	4	3	0	31265000	11839000	16714000	2712200	0

exp 552	BIR3 IP I										
Protein IDs	Majority protein IDs	Gene names TAIR	Peptide counts (all)	Peptides R1Col0	Peptides R2BIR3YFP		Intensity	Intensity R1Col0	Intensity R2BIR3YFP		
sp|Q9SRH6|HIR3_ARATH	sp|Q9SRH6|HIR3_ARATH	HIR2	5	3	4		181210000	69031000	112180000		

exp 552	BIR3 IP II		BIR3-GFP	BIR3-GFP	BIR3-GFP						
Protein IDs	Protein names	Gene names TAIR	Peptides R1	Peptides R2	Peptides R3		Intensity	Intensity R1	Intensity R2	Intensity R3	
Q9CAR7	Hypersensitive-induced response protein 2	HIR1	8	5	5		1293300000	414700000	254960000	623670000	
Q9SRH6	Hypersensitive-induced response protein 3	HIR2	7	7	5		4053400000	1127300000	718500000	2207600000	
Q9FM19	Hypersensitive-induced response protein 1	HIR4	5	4	3		290260000	56115000	59248000	174900000	

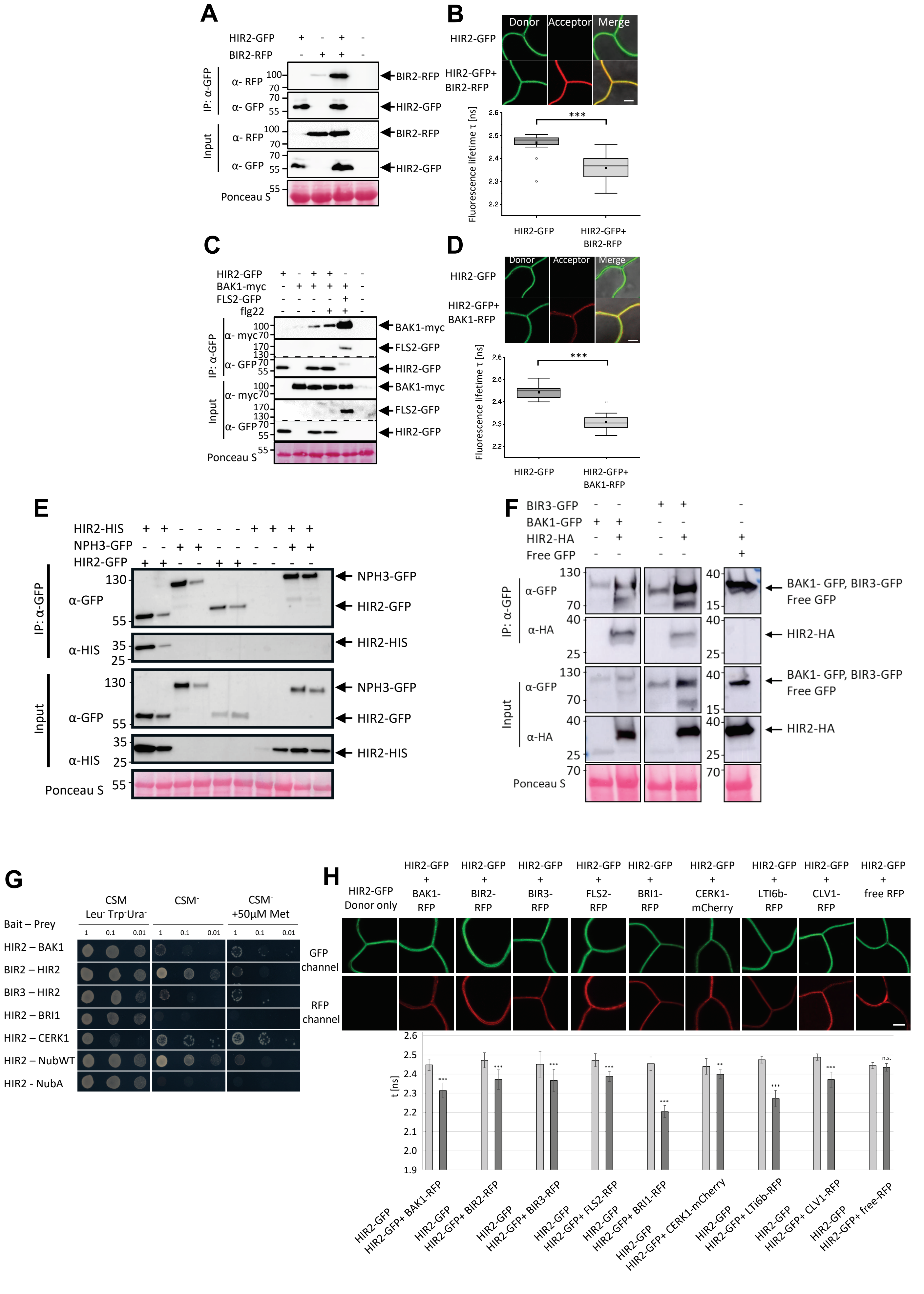

A
HIR2-GFP
BIR2-RFP
IP: α-GFP
α- RFP
α- GFP
Input
Ponceau S
BIR2-RFP
HIR2-GFP
B
Donor
Acceptor
Merge
HIR2-GFP
HIR2-GFP+
BIR2-RFP
Fluorescence lifetime τ [ns]
***
C
HIR2-GFP
BAK1-myc
FLS2-GFP
flg22
α- myc
BAK1-myc
FLS2-GFP
HIR2-GFP
D
HIR2-GFP+
BAK1-RFP
E
HIR2-HIS
NPH3-GFP
α-HIS
F
BIR3-GFP
BAK1-GFP
HIR2-HA
Free GFP
α-HA
BAK1- GFP, BIR3-GFP
G
Bait – Prey
CSM
Leu- Trp-Ura-
CSM-
+50µM Met
HIR2 – BAK1
BIR2 – HIR2
BIR3 – HIR2
HIR2 – BRI1
HIR2 – CERK1
HIR2 – NubWT
HIR2 - NubA
H
HIR2-GFP
Donor only
BIR3-
RFP
FLS2-
BRI1-
CERK1-
mCherry
LTI6b-
CLV1-
free RFP
GFP
channel
RFP
channel
t [ns]
n.s.
HIR2-GFP+ BAK1-RFP
HIR2-GFP+ BIR2-RFP
HIR2-GFP+ BIR3-RFP
HIR2-GFP+ FLS2-RFP
HIR2-GFP+ BRI1-RFP
HIR2-GFP+ CERK1-mCherry
HIR2-GFP+ LTI6b-RFP
HIR2-GFP+ CLV1-RFP
HIR2-GFP+ free-RFP

Appendix Figure S1: HIR2 interacts with several plasma membrane receptors/proteins. (related to Figure 1)

(A, C) Co-IP of HIR2 and BIR2 **(a)** or BAK1-myc **(c)** transiently expressed in *Nicotiana benthamiana* using GFP-traps were detected with α-GFP, α-RFP (BIR2), and α-myc (BAK1). Input shows expression of the individual proteins detected with antibodies against the respective tags. Infiltration of p19 served as negative control. Ponceau S staining shows protein loading. FLS2 BAK1 interaction served as a positive control for flg22-induced interaction. **(B, D)** Confocal imaging of *Nicotiana benthamiana* leaves expressing HIR2-GFP, and BIR2-RFP **(B)** or BAK1-RFP **(D)**. HIR2-GFP acted as donor for FRET-FLIM measurements with bi-exponential curve fitting in a defined region of interest covering the plasma membrane. Fluorescence lifetime was quantified and mean t values presented +/- SD. **(E)** HIR2-HA, BIR3-GFP and BAK1-GFP were expressed in Arabidopsis protoplasts*.* Co-IPs of BIR3-GFP and BAK1-GFP using GFP-traps were detected with α-GFP, and α-HA antibodies. Input shows expression of the individual proteins detected with antibodies against the respective tags. **(F)** Co-IP of HIR2-GFP and HIR2-HIS and GFP-NPH3 transiently expressed in *Nicotiana benthamiana* using GFP-traps were detected with α-GFP or α-HIS (HIR2),. Input shows expression of the individual proteins detected with antibodies against the respective tags. **(G)** Split-ubiquitin growth assay of mated yeast, carrying the plasmids for expression of the indicated proteins either as "bait" or "prey". The yeast was dropped in three consecutive 1:10 dilutions on selective media for vector transformation (CSM- Leu- , Trp-, Ura-). For selection of positive interactions, yeast was dropped on nutrient-deficient CSM$^-$ medium (Leu- , Trp- , Ura- , Ade-, His-) and CSM- with addition of 50 µM methionine. The expression of NubWT protein served as positive control and the NubA protein was used as negative control. Growth was documented after one day for vector selection and after two days for interaction selection. **(H)** HIR2-GFP and BAK1-RFP, BIR2-RFP, BIR3-RFP, FLS2-RFP, BRI1-RFP, CERK1-mCherry, LTI6b-RFP, CLV1-RFP and free RFP were transiently expressed in *Nicotiana benthamiana.* Confocal images of *Nicotiana benthamiana* leaves expressing HIR2-GFP and the indicated receptor-fluorophore fusions were taken 2 days after infiltration. FRET-FLIM analyses were performed with HIR2-GFP acting as the donor, and the GFP fluorescence lifetime was measured as described in **(B)**. For comparability BRI1 from Fig 1D is shown here again. Scale bars represent 5 µm. (*** $p \leq 0.001$, ** $p \leq 0.01$, ns $p > 0.5$). The assays were repeated at least twice with similar results.

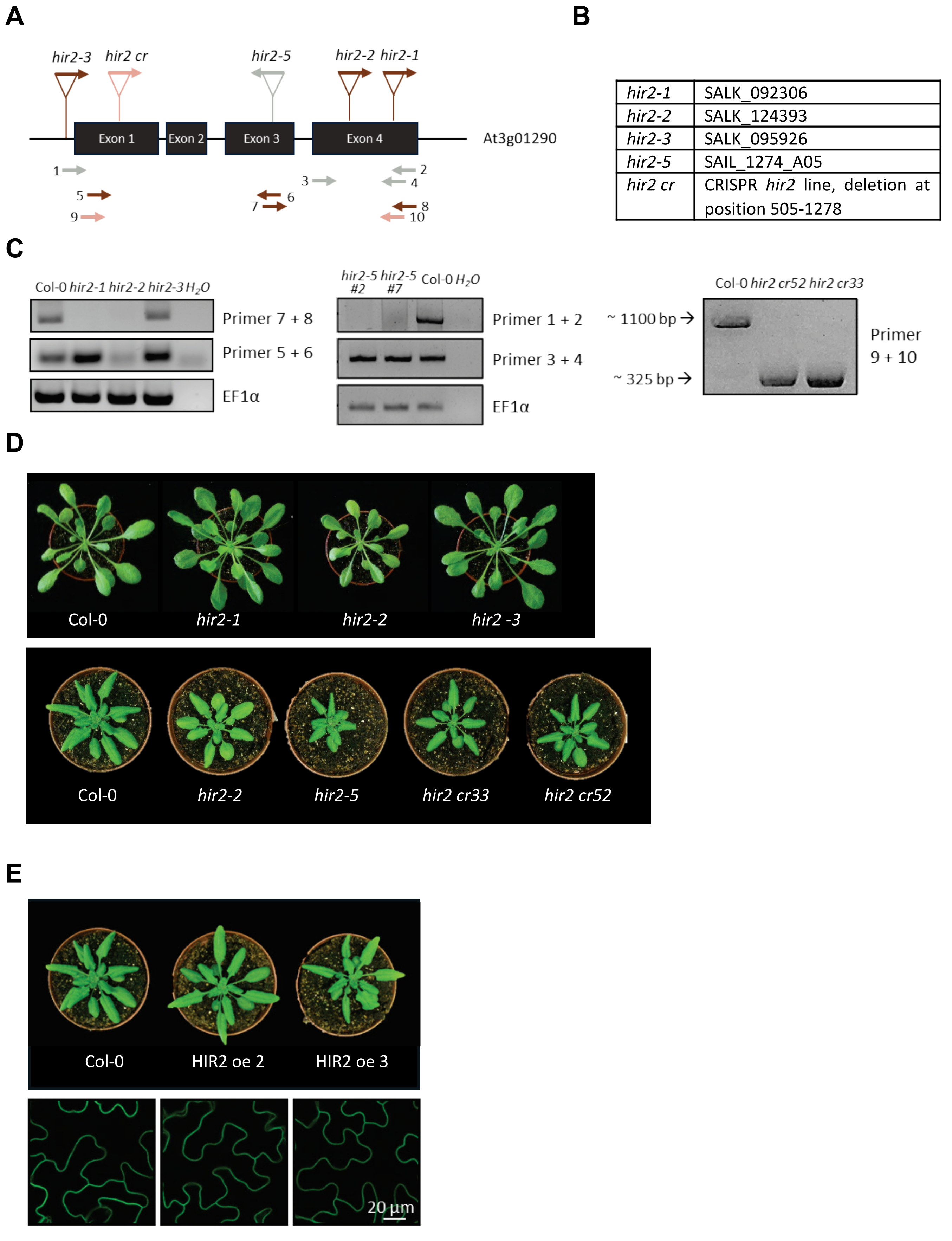

Appendix Figure S2: *hir2* mutant characterization. (related to Figure 2 and 3)
(A) Gene model of HIR2 with exon-intron structure and the positions of the T-DNA insertions and the deletion induced by CRISPR/Cas9. **(B)** Names and stock numbers of the indicated *hir2* mutants. **(C)** Semiquantitative PCR with the indicated primers and mutant background and EF1α as housekeeping gene control. **(D)** Representative pictures of 5-week-old plants of the indicated genotypes. **(E)** Representative picture of 5-week-old plants expressing Ubi-1 promotor driven HIR2-GFP and confocal images showing membrane localized expression of HIR2-GFP.

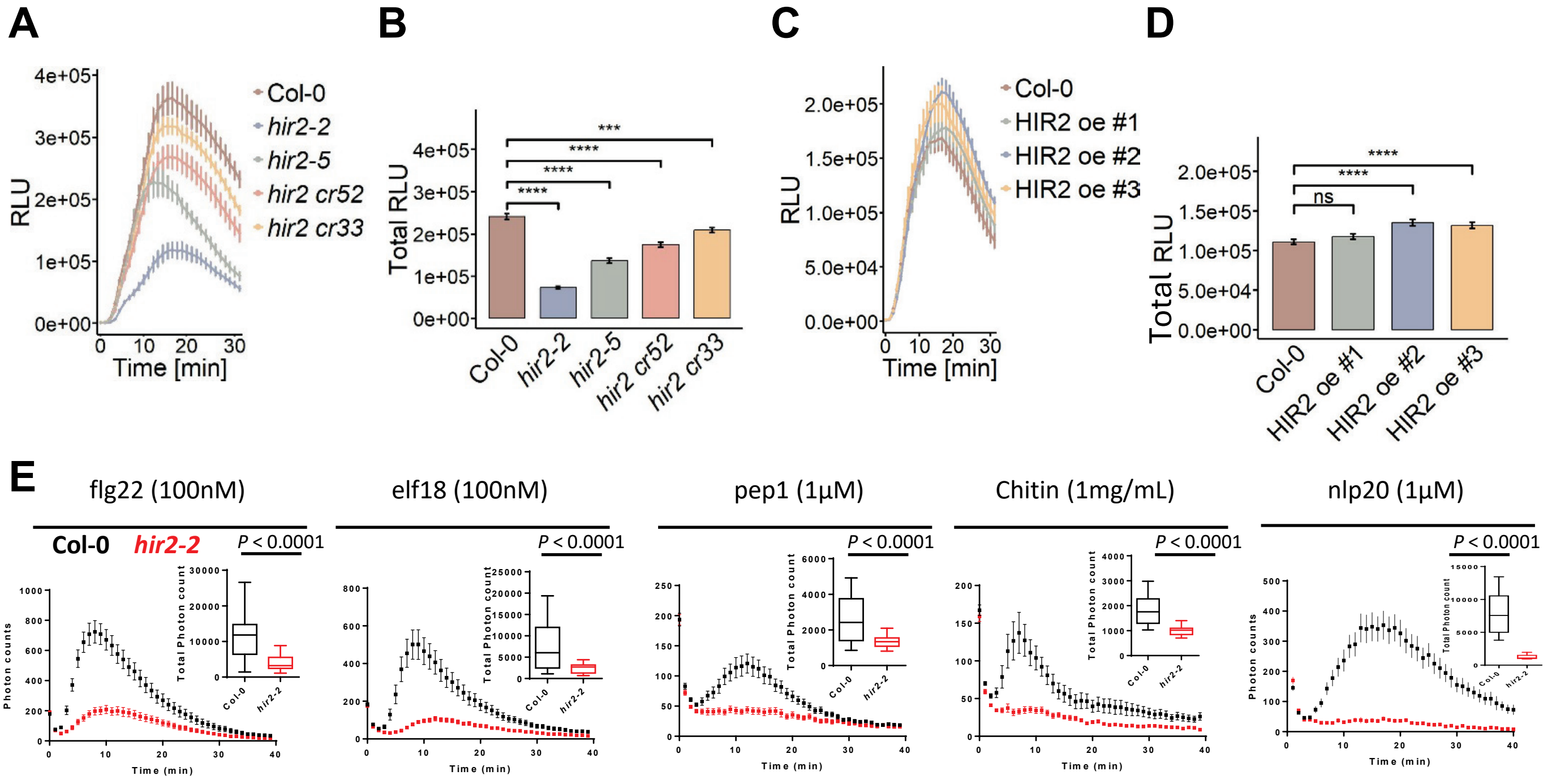

Appendix Figure S3: Mutant and overexpressor phenotypes after various MAMP treatments. (related to Figure 3)
Over-time **(A, C)** and total **(B, D)** reactive oxygen species (ROS) production of 5-week-old plants of the indicated genotypes. ROS production was measured as relative light units (RLU) after the addition of 1 µM flg22**. (A)** shows the over-time ROS production for the total ROS shown in (B) and in the right panel of Figure 3b **(E)** Over-time and total ROS production after triggering with the indicated MAMPs. The values represent means +/- SE , n=12. Experiments were repeated at least twice with similar results. Significant differences compared to Col-0 are determined by Student's t-test (**** p ≤ 0.0001; *** p ≤ 0.001; ns p > 0.05).

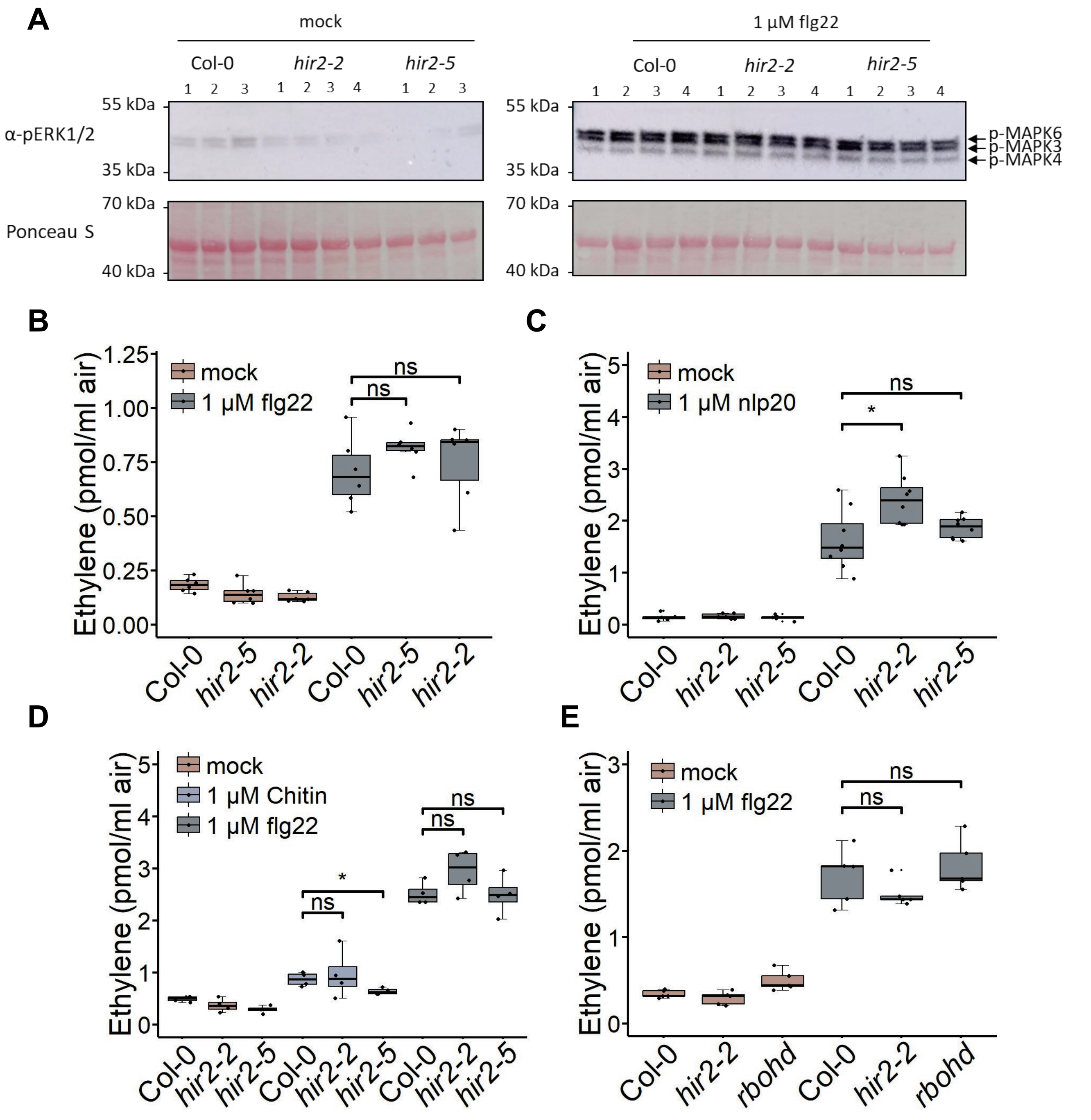

Appendix Figure S4: Loss of HIR2 does not impact MAP kinase phosphorylation and ethylene production. (related to Figure 3)

(A) Detection of MAP kinase phosphorylation in 7-day-old Col-0, *bak1-4, hir2-2,* and *hir2-5* seedlings after water (mock) or 1 µM flg22 treatment. The proteins were extracted 10 minutes after the treatments and separated by SDS-PAGE. Phosphorylation of MAP kinases was detected with α-phospho p44/p42 antibody. Ponceau S staining shows protein loading. **(B-E)** Ethylene production of 5-week-old Col-0, *hir2-2,* and *hir2-5* plants measured by gas chromatography 4h after adding 1 µM flg22 **(B)**, 1 µM nlp20 **(C)**, 1 µM chitin or 1 µM flg22 **(D)** and 1 µM flg22 to leave discs of *hir2-2* and *rbohD* mutant plants **(E)**. Experiments were repeated at least twice with similar results. Significant differences compared to Col-0 were determined by Student's t-test (ns $p > 0.05$; * $p \leq 0.05$).

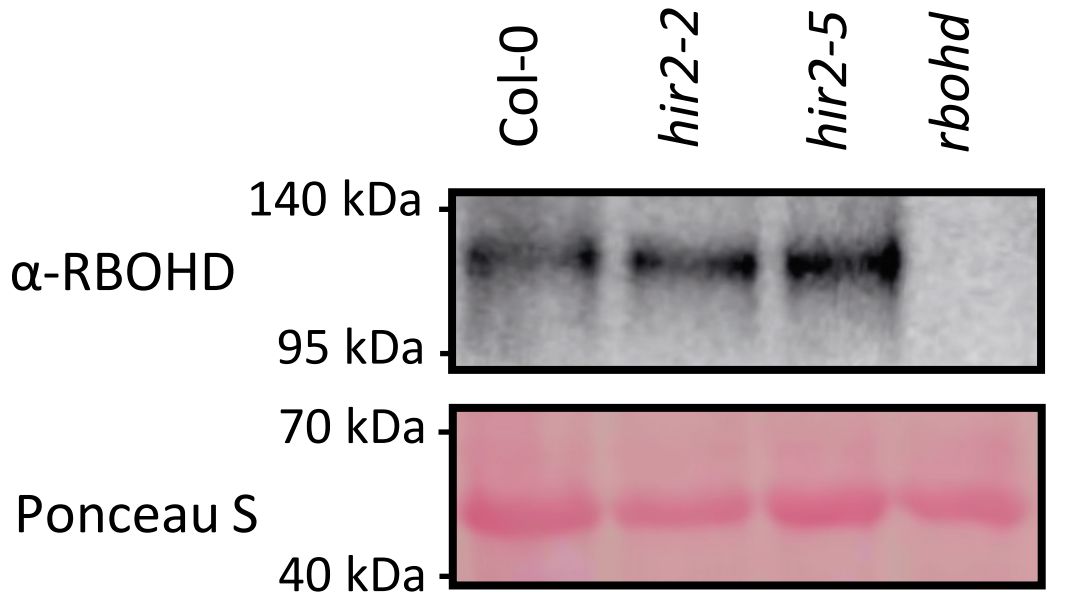

Appendix Figure S5: Expression of RbohD is not impaired in the *hir2* mutants. (related to Figure 3)
Detection of RbohD in 6-week-old Col-0, *hir2-2, hir2-5,* and *rbohd* plants. The proteins were extracted and separated by SDS-PAGE. Expression of RbohD was detected with α-RbohD antibody. Ponceau S staining shows protein loading.

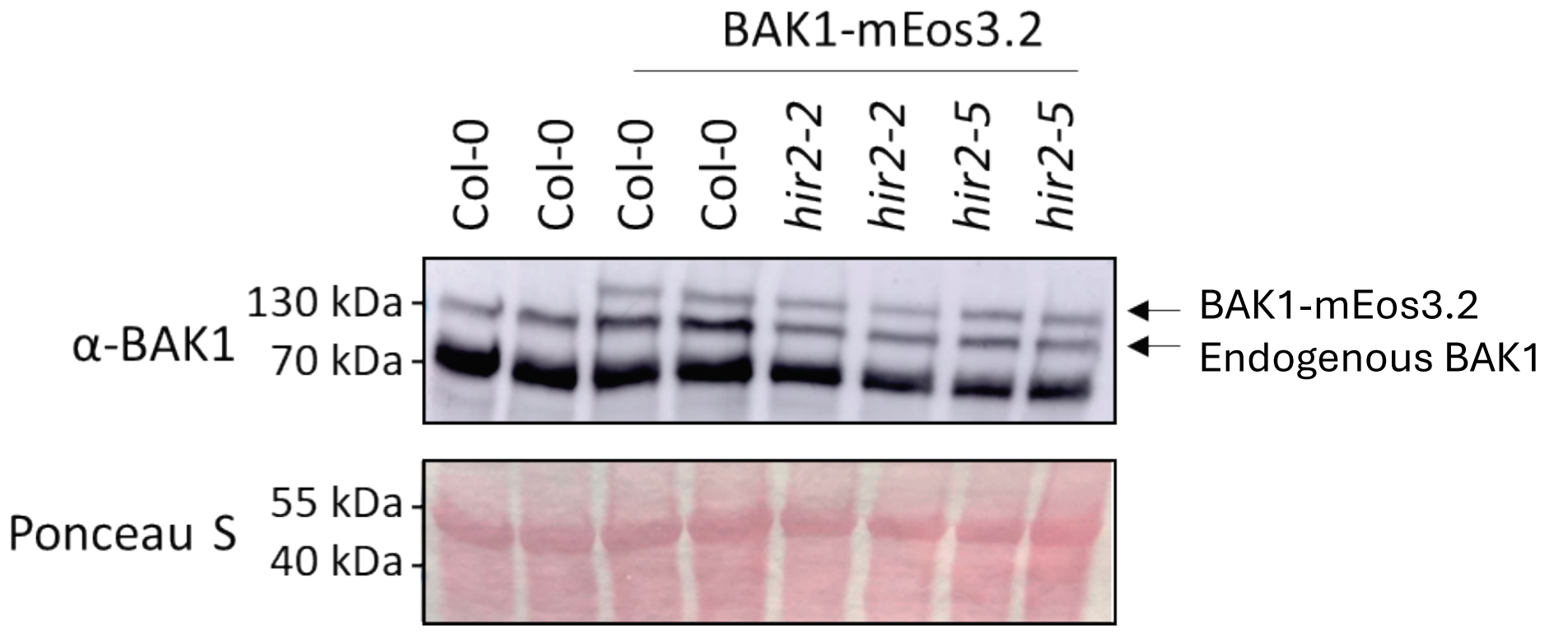

Appendix Figure S6: Expression control of BAK1-mEos3.2 in Col-0, *hir2-2,* and *hir2-5* background. (related to Figure 4) Detection of BAK1 in two independent stable Col-0-BAK1-mEos3.2, *hir2-2*-BAK1-mEos3.2, and *hir2-5*-BAK1-mEos3.2 lines. The proteins were extracted from 6-week-old leaves and separated by SDS-PAGE. Expression of BAK1 was detected with α-BAK1 antibody. Ponceau S staining shows protein loading.

A

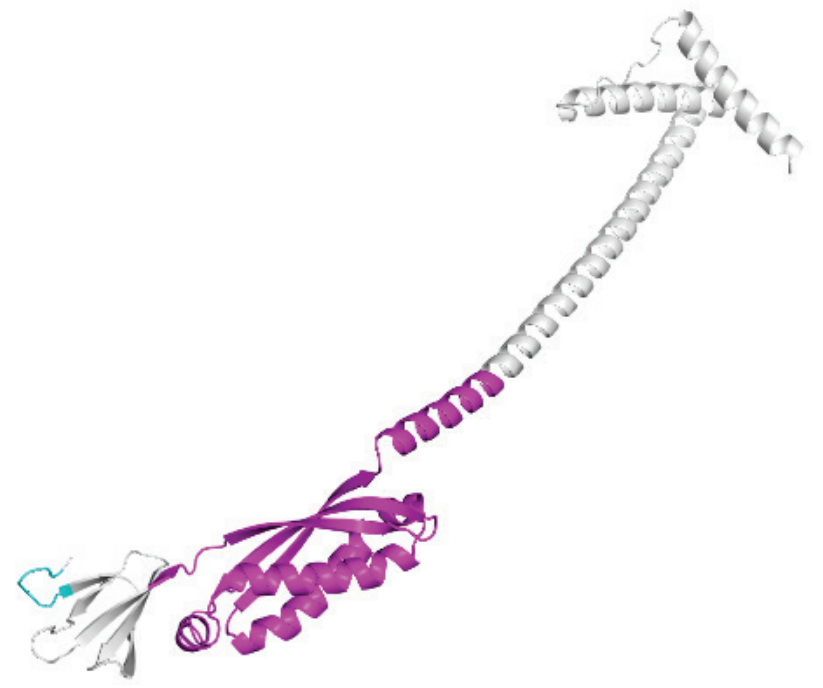

```
  1 MGNLFCCVLV KQSDVAVKER FGKFQKVLNP GLQFVPWVIG DYVAGTLTLR LQQLDVQCET
 61 KTKDNVFVTV VASIQYRVLA DKASDAFYRL SNPTTQIKAY VFDVIRACVP KLNLDDVFEQ
121 KNEIAKSVEE ELDKAMTAYG YEILQTLIID IEPDQQVKRA MNEINAAARM RVAASEKAEA
181 EKIIQIKRAE GEAESKYLSG LGIARQRQAI VDGLRDSVLG FAGNVPGTSA KDVLDMVMMT
241 QYFDTMRDIG ATSKSSAVFI PHGPGAVSDV AAQIRNGLLQ ANNAS
```

B

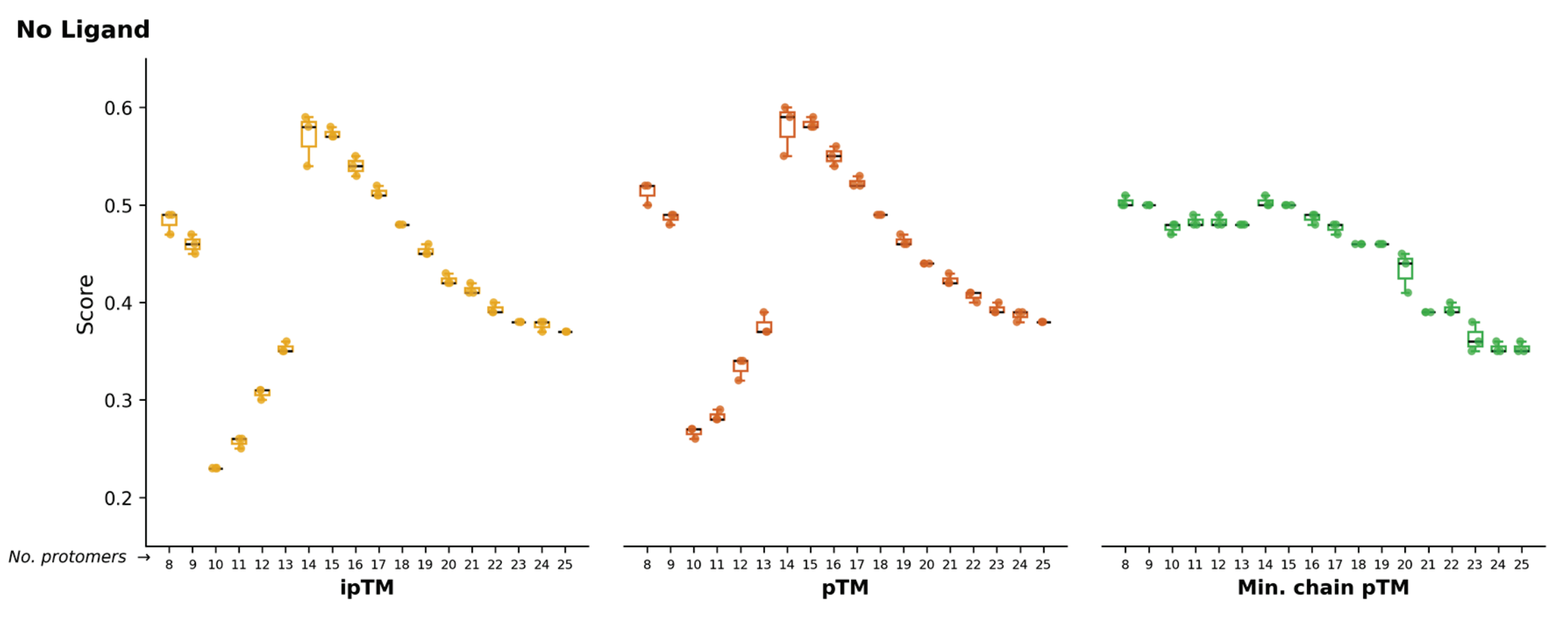

Appendix Figure S7 : SPFH domain prediction in AlphaFold modelled HIR2 and protomer screening of HIR2 oligomers

(A) Based on Interpro prediction the HIR2 protein (grey) contains a BAND7/SPFH domain (Interpro 036013) that is localized between residue 41 and 171 (pink) or a stomatin-like domain (Interpro 050710) that contains residue 10-278 (black), N-terminal membrane attachment site (blue), predicted myristoylation and palmitoylations sites and C58 underlined. **(B)** AlphaFold3 modelling of HIR2 assemblies across different oligomeric states. The x-axis indicates the number of protomers modelled in each assembly, whereas the y-axis shows the corresponding ipTM, pTM, and minimum per-chain pTM scores.

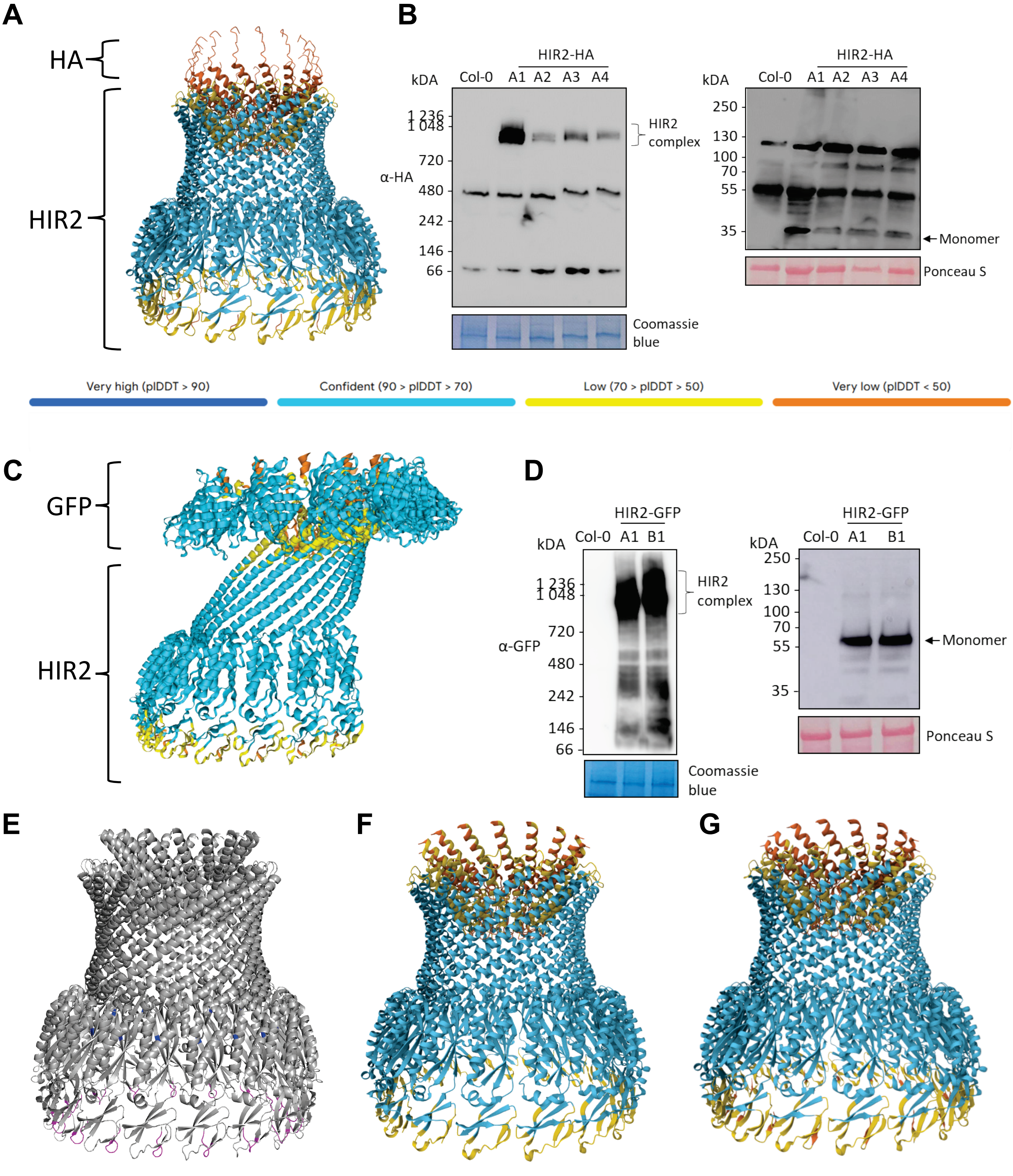

Appendix Figure S8: AlphaFold modeling of tagged and mutated HIR2 versions. (related to Figure 6)

(A) AlphaFold 3 model of a 17mer of the HA-tagged HIR2. **(B)** HIR2-HA expressed in two independent stably transformed Arabidopsis lines and separated by blue native PAGE (left) and SDS PAGE (right). Coomassie and Ponceau S staining, respectively, shows protein loading. **(C)** AlphaFold 2 model of a 8mer of GFP-tagged HIR2. **(D)** HIR2-GFP expressed in two independent stably transformed Arabidopsis lines and separated by blue native PAGE (left) and SDS PAGE (right). Coomassie and Ponceau S staining, respectively, shows protein loading. **(E)** AlphaFold 3 model of a 17mer of HIR2 with C58 marked in blue and N6 marked in magenta. **(F)** AlphaFold3 model of HIR2$^{\Delta N6}$ (17mer). **(G)** AlphaFold 3 model of HIR2^{C58S} (17mer).

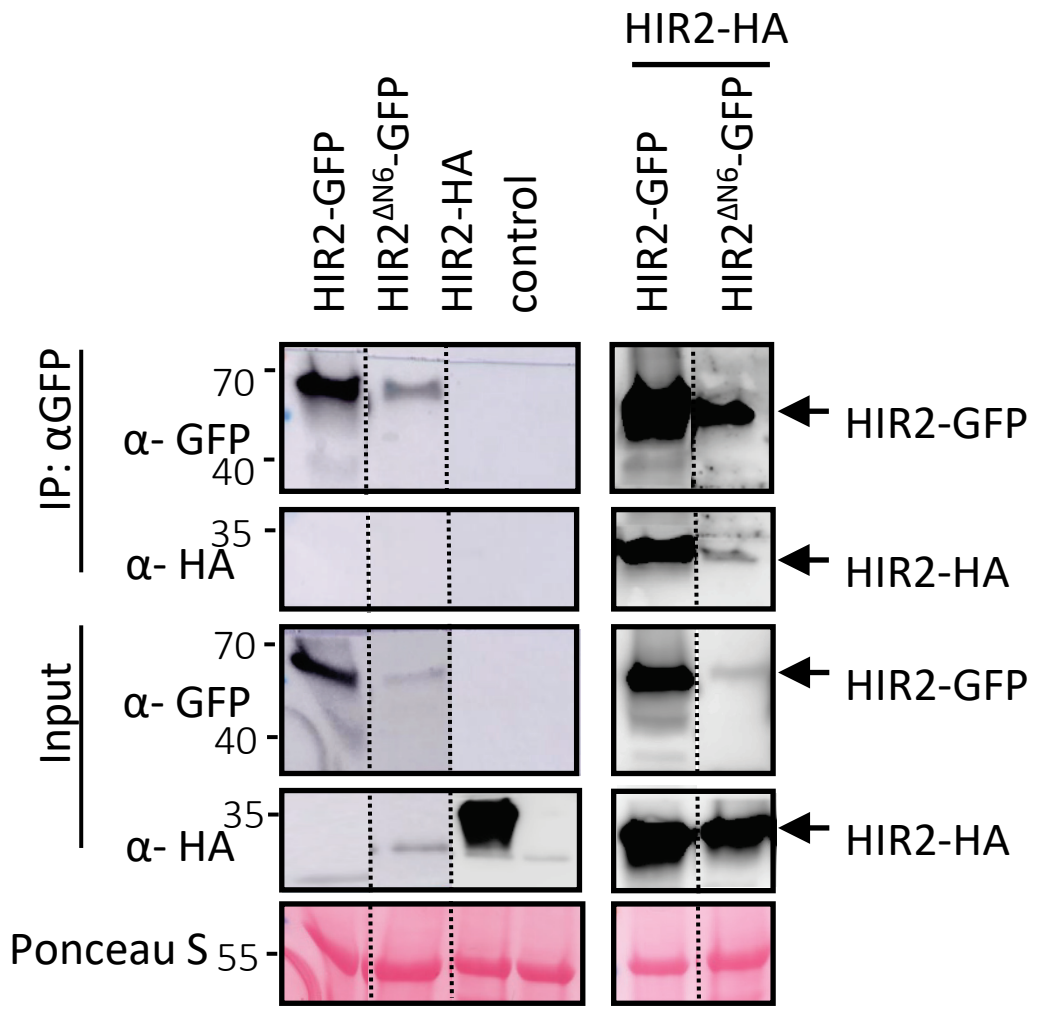

Appendix Figure S9: Self-interaction of HIR2 and HIR2 variants

(A) Co-IP of proteins extracted from *Nicotiana benthamiana* leaves transiently expressing HIR2-GFP, HIR2-HA, and HIR2$^{\Delta N6}$-GFP Proteins were detected using α-GFP or α-HA antibodies. Input shows expression of the individual proteins detected with antibodies against the respective tags. Ponceau S staining was used to verify equal protein loading. Experiments were repeated at least twice with similar results.

Appendix Table S2: Primers used in this study. (related to Methods)

Name	Characteristic	Sequence (5’ → 3’)
pBAK1-1_F	Sequencing	GCGGACAAAGGTGGAGGAA
pBAK1-621_F	Sequencing	GGACCAGATGGATTATTCAGC
pBAK1-1131_F	Sequencing	GGCGATAACTTGGTTTCTCT
BAK1-CDS-1_F	Sequencing	TCTGAACAATGGAACGAAGA
BAK1-CDS-608_F	Sequencing	CCAAGTTGACTCCCCTTC
BAK1-CDS-1294_F	Sequencing	GAAGAGTTTGAAGCCGTGGTT
mEos3.2_F	Sequencing	AAGGGAGGTGGAGGAGGTT
nosT-1_F	Sequencing	AATCGATCGTTCAAACATTTGGC
mEos3.2-218_R	Sequencing	GCGGTAGTGAGAATATCGAAAG
mEos3.2_GG-D-E_F	GoldenGate CloningD-E Modul LI	TTATGGTCTCTAAGGGAATGTCTGCTATCAAGCCT
mEos3.2-STOP_GG-D-E_R	GoldenGate CloningD-E Modul LI	TTAAGGTCTCTGATTTTATCTTCTAGCGTTATCTGGAA
Linker-PA-GFP_F	GoldenGate CloningD-E Modul LI	aggcggtggaagtggtggcggaggtagcATGGTGAGCAAGGGCGAA
PA-GFP_GG-D-E_R (stop)	GoldenGate CloningD-E Modul LI	TTAAGGTCTCTGATTTTATCACTTGTAAAGCTCGTCCA
PAGFP_F	Sequencing	ATGGTGAGCAAGGGCGAAGA
PAGFP_R	Sequencing	TCACTTGTAAAGCTCGTCCATTC
HIR2_GG_B-D_F	GoldenGate Cloning B-D Modul LI	TTATGGTCTCTTCTGAACAATGGGGAATCTTTTCTGTTGC
HIR2_GG_B-D_R	GoldenGate Cloning B-D Modul LI	TTAAGGTCTCTCCTTGGAGGCATTGTTGGCCTG
pFast-275_R	Sequencing	CTATGTGTGTTCTGCATTTGGG
pFast_F	Sequencing	tgagCTTCAAGTGTATGTAGG
pFast-888_F	Sequencing	CCTGCATTATCAAAGCAGTGAC
pFast-1797_F	Sequencing	GCATGTGTTGAGCCAGTAGCT
pFast-2518_F	Sequencing	GTTCATCTACAAGGTGAAGCTG
pHIR2_GG_A-B_F	GoldenGate Cloning A-B Modul LI	TTATGGTCTCTGCGGgcatctcctggacaaaaga
pHIR2_GG_A-B_R	GoldenGate Cloning A-B Modul LI	TTAAGGTCTCTCAGAtattctgaacaagaagcaagaa
LII-BB10_F	Sequencing	CTGGCTGGTGGCAGGATAT
LII-BB10_R	Sequencing	GCGGCCTTTTTACGGTTCCT
HIR2_cds_F	Sequencing	ATGGGGAATCTTTTCTGTTGC
HIR2_cds_R	Sequencing	GGAGGCATTGTTGGCCTGTA
HIR2_C67>S-new_F	Mutagenesis cys 6 and 7 to ser	GAATCTTTTCTCTTCCGTGCTTGTGA
HIR2_C67>S-new_R	Mutagenesis cys 6 and 7 to ser	TCACAAGCACGGAAGAGAAAAGATTC
HIR2-G2A_F	Mutagenesis gly 2 to ala in HIR2	CACCATGGCGAATCTTTTCTGTTGCGTGCT
HIR2-G2A_R	Mutagenesis gly 2 to ala in HIR2	GATTCGCCATGGTGAAGGGGGCGGCCGC
HIR2-G2AC76S_F	Mutagenesis gly 2 to ala in HIR2-C6.7S	CACCATGGCGAATCTTTTCTCTTCCGTGCT
sgRNA1_F	Crispr HIR2	attgTGTGAAGCAATCAGATGTTG
sgRNA1_R	Crispr HIR2	aaacCAACATCTGATTGCTTCACA
sgRNA2_F	Crispr HIR2	attgAGACGGCGCCTGGACCGTGA
sgRNA2_R	Crispr HIR2	aaacTCACGGTCCAGGCGCCGTCT
Sail-LB	T-DNA sail	gcttcctattatatcttcccaaattacc
a_SAIL_1274_A05	Right primer	TCAGCAACTCGATGTTCAGTG
b_SAIL_1274_A05	Left primer	CGATTTTTCTCTCGCAAACAG
gRNA1_HIR2_Fw	Genotyping CRISPR_HIR2	CCATGACTGCTTATGGTTACGA
gRNA1_HIR2_Re	Genotyping CRISPR_HIR2	GAGTCCCGACAGGTACTTTGAC
gRNA2_HIR2_Fw	Genotyping CRISPR_HIR2	ACACTCCTGCGACTTTCTTCTC
gRNA2_HIR2_Re	Genotyping CRISPR_HIR2	CCTTTGTTTTGGTTTCACACTG
HIR2_N6>X_fw	Deletion of 6 Bases at N-Terminus (Site-directed mutagenesis)	GTGCTTGTGAAGCAATC
HIR2_N6>X_rv	Deletion of 6 Bases at N-Terminus (Site-directed mutagenesis)	CATTGTTCAGATATTCTGAAC
pHIR2_288_fw	Sequencing HIR2 promotor	CAAGAAACGGGCAACCAGTG
pHIR2_890_rv	Sequencing HIR2 promotor	AAAAGTCGTTGGTCCTTGCG
pHIR2_44_rv	Sequencing HIR2 promotor	CCAGCCTCGGATCAAGTAACCG
HIR2_C58S:1_FWD	Mutagenesis cysteine 58 to serine in HIR2	CGATGTTCAGagcGAAACCAAAAC
HIR2_C58S:1_REV	Mutagenesis cysteine 58 to serine in HIR2	AGTTGCTGAAGACGAAG
