# Supplementary material for: The Arabidopsis SPFH protein HIR2 modulates receptor signaling and plasma membrane organization
Source: EMBO J. 2026 Jul 29;45(17):6176–96. doi: 10.1038/s44318-026-00877-y (PMC13534476; doi:10.1038/s44318-026-00877-y)
Supplement: Supplementary file 2 [file 44318_2026_877_MOESM2_ESM.pdf]

THE EMBO JOURNAL

The Arabidopsis SPFH protein HIR2 modulates receptor signaling and plasma membrane organization

Hannah Weber, Alexandra Ehinger, Dagmar Kolb, Vahid Fallazadeh-Mamaghani, Thierry Halter, Mauricio Contreras, Mirita Franz-Wachtel, Nora Schäfer, Sven zur Oven-Krockhaus, Julien Gronnier, Claudia Oecking, Cyril Zipfel, Klaus Harter, and Birgit Kemmerling

Corresponding author(s): Birgit Kemmerling (birgit.kemmerling@zmbp.uni-tuebingen.de)

Review Timeline:

Submission Date:	15th May 25
Editorial Decision:	7th Jul 25
Revision Received:	2nd Jun 26
Editorial Decision:	16th Jun 26
Revision Received:	2nd Jul 26
Accepted:	6th Jul 26

Editor: William Teale

Transaction Report:

(Note: With the exception of the correction of typographical or spelling errors that could be a source of ambiguity, letters and reports are not edited. Depending on transfer agreements, referee reports obtained elsewhere may or may not be included in this compilation. Referee reports are anonymous unless the Referee chooses to sign their reports.)

1st Editorial Decision 7th Jul 2025

Dear Dr Kemmerling,

Thank you again for the submission of your manuscript entitled "Arabidopsis HYPERSENSITIVE INDUCED REACTION 2 affects surface receptor pathways and organization" and for your patience during the review process. We have now received the reports from the referees, which I copy below.

As you can see from their comments, while all referees expressed a degree of enthusiasm for the timeliness of your study, Referee #3 raised some important concerns over robustness (stringency of controls) and mechanistic depth (with respect to, for example, ROS production [a point also raised by Reviewer #1] and oligomerisation).

It could be that these experiments will take a considerable time investment to complete. However, I could imagine that they might be feasible if the relevant lines have already been constructed. I therefore suggest we discuss the project in the next week or two over Zoom. Please, once you and your co-authors have had time to digest the reports, contact me with some convenient time-slots (my diary is currently fairly flexible). Based on this discussion, I might then be able to to invite you to address the comments of all referees in a revised version of the manuscript. In the meantime, I will label the manuscript as under revision in our system and supply the relevant revision instructions below my sign off.

I would also like to point out that as a matter of policy, competing manuscripts published during a revision period will not be taken into consideration in our assessment of the novelty presented by your study ("scooping" protection). We may extend this 'scooping protection policy' beyond the usual 3 month revision timeline to cover the period required for a full revision to address the essential experimental issues. Please contact me if you see a paper with related content published elsewhere to discuss the appropriate course of action.

When preparing your letter of response to the referees' comments, please bear in mind that this will form part of the Review Process File, and will therefore be available online to the community. For more details on our Transparent Editorial Process, please visit our website: https://www.embopress.org/page/journal/14602075/authorguide#transparentprocess

Again, please contact me at any time during revision if you need any help or have further questions.

Thank you very much again for the opportunity to consider your work for publication. I look forward to your revision.

Best regards,

William

William Teale, Ph.D.
Editor
The EMBO Journal

When submitting your revised manuscript, please carefully review the instructions below and include the following items:

1) a .docx formatted version of the manuscript text (including legends for main figures, EV figures and tables). Please make sure that the changes are highlighted to be clearly visible.

2) individual production quality figure files as .eps, .tif, .jpg (one file per figure).

3) a .docx formatted letter INCLUDING the reviewers' reports and your detailed point-by-point response to their comments. As part of the EMBO Press transparent editorial process, the point-by-point response is part of the Review Process File (RPF), which will be published alongside your paper.

4) a complete author checklist, which you can download from our author guidelines (https://wol-prod-cdn.literatumonline.com/pb-assets/embo-site/Author Checklist%20-%20EMBO%20J-1561436015657.xlsx). Please insert information in the checklist that is also reflected in the manuscript. The completed author checklist will also be part of the RPF.

5) Please note that all corresponding authors are required to supply an ORCID ID for their name upon submission of a revised manuscript.

6) We require a 'Data Availability' section after the Materials and Methods. Before submitting your revision, primary datasets produced in this study need to be deposited in an appropriate public database, and the accession numbers and database listed under 'Data Availability'. Please remember to provide a reviewer password if the datasets are not yet public (see

https://www.embopress.org/page/journal/14602075/authorguide#datadeposition). If no data deposition in external databases is needed for this paper, please then state in this section: This study includes no data deposited in external repositories. Note that the Data Availability Section is restricted to new primary data that are part of this study.

Note - All links should resolve to a page where the data can be accessed.

7) When assembling figures, please refer to our figure preparation guideline in order to ensure proper formatting and readability in print as well as on screen:
http://bit.ly/EMBOPressFigurePreparationGuideline

Please remember: Digital image enhancement is acceptable practice, as long as it accurately represents the original data and conforms to community standards. If a figure has been subjected to significant electronic manipulation, this must be noted in the figure legend or in the 'Materials and Methods' section. The editors reserve the right to request original versions of figures and the original images that were used to assemble the figure.

8) For data quantification: please specify the name of the statistical test used to generate error bars and P values, the number (n) of independent experiments (specify technical or biological replicates) underlying each data point and the test used to calculate p-values in each figure legend. The figure legends should contain a basic description of n, P and the test applied. Graphs must include a description of the bars and the error bars (s.d., s.e.m.).

9) We would also encourage you to include the source data for figure panels that show essential data. Numerical data can be provided as individual .xls or .csv files (including a tab describing the data). For 'blots' or microscopy, uncropped images should be submitted (using a zip archive or a single pdf per main figure if multiple images need to be supplied for one panel). Additional information on source data and instruction on how to label the files are available at .

10) We replaced Supplementary Information with Expanded View (EV) Figures and Tables that are collapsible/expandable online (see examples in https://www.embopress.org/doi/10.15252/embj.201695874). A maximum of 5 EV Figures can be typeset. EV Figures should be cited as 'Figure EV1, Figure EV2" etc. in the text and their respective legends should be included in the main text after the legends of regular figures.

- For the figures that you do NOT wish to display as Expanded View figures, they should be bundled together with their legends in a single PDF file called *Appendix*, which should start with a short Table of Content. Appendix figures should be referred to in the main text as: "Appendix Figure S1, Appendix Figure S2" etc. See detailed instructions regarding expanded view here: .

- Additional Tables/Datasets should be labelled and referred to as Table EV1, Dataset EV1, etc. Legends have to be provided in a separate tab in case of .xls files. Alternatively, the legend can be supplied as a separate text file (README) and zipped together with the Table/Dataset file.

11) At EMBO Press we ask authors to provide source data for the main manuscript figures. Our source data coordinator will contact you to discuss which figure panels we would need source data for and will also provide you with helpful tips on how to upload and organize the files.

12) Our journal encourages inclusion of *data citations in the reference list* to directly cite datasets that were re-used and obtained from public databases. Data citations in the article text are distinct from normal bibliographical citations and should directly link to the database records from which the data can be accessed. In the main text, data citations are formatted as follows: "Data ref: Smith et al, 2001" or "Data ref: NCBI Sequence Read Archive PRJNA342805, 2017". In the Reference list, data citations must be labeled with "[DATASET]". A data reference must provide the database name, accession number/identifiers and a resolvable link to the landing page from which the data can be accessed at the end of the reference. Further instructions are available at .

13) In order to increase the reproducibility and reach of your work, The EMBO Journal includes a table of reagents that were used in the study. Please provide this along with your revisions.

Further instructions for preparing your revised manuscript:

Please check that the title and abstract of the manuscript are brief, yet explicit, even to non-specialists.

IMPORTANT: When you send the revision we will require
- a point-by-point response to the referees' comments, with a detailed description of the changes made (as a word file).
- a word file of the manuscript text
- individual production quality figure files (one file per figure)
- a complete author checklist, which you can download from our author guidelines (https://www.embopress.org/page/journal/14602075/authorguide).
- Expanded View files (replacing Supplementary Information)

Please see out instructions to authors
https://www.embopress.org/page/journal/14602075/authorguide#expandedview
- We ask authors to provide source data for the main manuscript figures. You will receive a separate email with instructions for providing source data with your revised manuscript, including how to upload and organize the files.

Please remember: Digital image enhancement is acceptable practice, as long as it accurately represents the original data and conforms to community standards. If a figure has been subjected to significant electronic manipulation, this must be noted in the figure legend or in the 'Materials and Methods' section. The editors reserve the right to request original versions of figures and the original images that were used to assemble the figure.

Further information is available in our Guide For Authors: https://www.embopress.org/page/journal/14602075/authorguide

The revision must be submitted online within 90 days; please click on the link below to submit the revision online before 5th Oct 2025.

https://emboj.msubmit.net/cgi-bin/main.plex

Referee #1:

The submitted manuscript by Weber and Ehinger et al. focuses on plant membrane organisation. To this end, the authors investigate plant-specific members of the stomatin/prohibitin/flotillin/HflK/C (SPFH) family, HIR proteins, particularly the HIR2 isoform. They show that HIR2 interacts with several integral membrane proteins, such as BIR2 and 3, BAK1, CERK1 and others, and is anchored to the inner leaflet of the plasma membrane through a hydrophobic domain and lipidation. The authors then connect HIR2 to BAK1-mediated signalling pathways using hir2 mutant plants. Using super-resolution microscopy, they show that HIR2 affects receptor kinase dynamics and clustering. Structural modelling using AlphaFold3, corroborated by blue native PAGE experiments, provides evidence of higher-order assemblies, suggesting the existence of a novel structural framework for spatiotemporal membrane organisation. The manuscript is timely and reads very well. The experimental results mostly appear well-performed with corresponding controls. The manuscript will appeal to researchers interested in membrane organisation, membrane signalling, plant immunity and plant developmental biology. Nevertheless, I still have several suggestions and comments regarding the manuscript.

Major concerns
1)The authors state (page 7, lines 181 to 188), "The hir2 mutant phenotypes resemble those of robhD mutants. However, the accumulation of RbohD is not affected in hir2-2 or hir2-5 mutants, showing that the effect observed in hir2 mutants is not due to reduced RbohD levels." However, the authors show that the same is true for BAK1 (page 8, lines 227 to 228, Figure S7), and they discuss that the loss of HIR2 affects the membrane dynamics and organisation of BAK1. Are RBOHD dynamics and clustering affected in hir2 mutants? Could HIR2 interact with RBOHD similarly to all membrane proteins tested in the manuscript?

2)How do the dynamics and clustering of BAK1 in Col-O and hir2 mutants change upon signalling, e.g., after Flg22 application?

3)The authors observed that the BAK1 diffusion coefficient is significantly lower in the hir2 mutants. If HIR2 restricts BAK1 in the membrane nanodomains, I would expect the diffusion coefficient to increase in the hir2 mutants.

4)The authors identified the HIR proteins using co-immunoprecipitation with BIR2 and BIR3, followed by mass spectrometry analysis. The results are then presented in Table S1. However, only the HIR proteins are shown. Did the authors also identify other proteins? In Table S1, HIR2 (Uniprot ID Q9CAR7) does not contain cysteines at positions 6 and 7, unlike HIR3 (Uniprot ID Q9SRH6).

5)Figure 3b contains two graphs showing the total ROS production. Are there two independent experiments?

Minor comments
1)It might be better to specify the split-ubiquitin yeast two-hybrid assay (page 5, line 138) to avoid confusion with the traditional Y2H assay. On line 139, the reference should be to Figure S1e, not to Figure 1e. Similarly, on line 143, it should be to Figure S1f, not Figure 1f. Also, on page 7, line 181, it should not be to Figure 5a-d, but Figure S5.
2)There is no reference to FirstGlance.
3)Page 11, line 316, a new type of membrane compartment might be an overstatement.
4)On page 18, line 527, the W5 solution is not defined.

Referee #2:

This is a very well-written paper that highlighted the functions of HIR2 protein in plant growth and immune responses. I commend the authors on the discoveries made, as well as multiple-prong approaches taken to demonstrate the functions of HIR2 in both plant growth and immunity. HIR2 belongs to a group of SPFH family that have been shown to influence membrane organization in Arabidopsis. The authors showed solid evidence that HIR2 interacted with multiple plasma membrane proteins, including major common co-receptors such as BAK1 and BIRs. The authors also reported the multimeric state of HIR2 via AlphaFold prediction, suggesting that HIR2 could serve as a spatial platform for membrane organization of membrane-occupying receptors.
There are a number of minor points I think the authors may want to address to improve the quality of this manuscript:

1) Line 145: The authors wrote "Specificity is not determined by interaction interfaces here". The authors may want to clarify this: did they mean the interaction interfaces as in nanodomains vs. non-nanodomains?
2) Line 196-197: The authors should compare the quantitative contribution of HIR2 to disease resistance against P. syringae with other mutants in the HIR family (HIR1, HIR 3, HIR4) to conclude that "HIR2 is required to establish plant immune responses quantitatively"
3) BAK1 dynamics being affected by HIR2: How does BAK1 diffusion coefficient changed after flg22 elicitation? Does the authors have any date to show that HIR2 influence BAK1 dynamics once ligand recognition happens using sptPALM? This is essential to connect BAK1 dynamics to its signaling and downstream responses.

4) Fig 1e: there is an issue with the input of lane # 2 (only CERK1-HA and no HIR2-GFP). The figure showed a band positive for HIR2-GFP and some residual signal in the IP: α-GFP blot when probed with α-GFP.

5) Line 327-330: The authors should elaborate their speculation of how the multimeric structure of HIR2 complexes could facilitate its role as a scaffolding protein for RKs, promoting RK-mediated responses.

6) The authors should also elaborate on the experimental details of the AlphaFold prediction and annotate the domain in the protein structure clearly in the result section (e.g., amino acid position of the N-terminus, SPFH domain, the three helices, etc.).

Referee #3:

Hannah Weber and co-authors described a study of HIR2 function in plant immunity with biochemistry, cell biology, and structural predictions. HIR2 is anchored to the inner leaflet of the plasma membrane in a nanodomain-like structure via a hydrophobic domain and S-acylation. HIR2 was found to interact with BIRs, which are leucine-rich repeat receptor kinases, as negative regulators of BAK1 complex formation and signaling. This research area is highly promising for understanding how surface biomolecular compartmentalization regulates molecular dynamics and function during immune responses. However, the experimental results do not clearly connect the observed imaging patterns of HIR2 and BIR3 to their functional roles in immunity. Additionally, immune defense assays in HIR2 mutant and overexpression lines were not conducted, limiting the integration of this information. The reviewer finds the work interesting but very preliminary, and cannot recommend at this stage.

1. The FRET-FLIM data show HIR2 interacts with all tested proteins, leading the authors to conclude that HIR2 is abundant. This conclusion seems illogical. If this abundance conclusion is meaningful, a control with an unrelated, highly expressed membrane protein (e.g., Signal peptide-Transmembrane domain-GFP) should be included. Also, the citation should refer to Figure S1f, not Figure 1f. Because in most of the case membrane associated protein will have positive FLIM-FRET results due to close distance by overexpression in tobacco.
2. The reviewer cannot discern enhanced growth in HIR2 overexpression lines (HIR2oe) in Figure S3, as claimed by the authors.
3. The authors cite another paper stating that the hir2-2 mutant shows increased bacterial and fungal growth, suggesting HIR2 is a positive regulator of immunity. However, hir2-2 has an additional T-DNA insertion, complicating the background. The reviewer questions why infection assays were not performed with the samples used in this study (e.g., 2-5, cr, and two OE lines).
4. While the hir2 mutants do not affect MAPK or ethylene production, the cause of compromised peptide-triggered ROS remains unclear. The discussion on RbohD and the hir2 mutant is interesting but lacks mechanistic investigation and insights into how PTI-triggered ROS is reduced, whether through RbohD, receptor interactions, or ligand binding in hir2 mutants.
5. Superresolution imaging of HIR2 and BIR3 was conducted, but without comparing PAMP-activated and unactivated samples. The MSD analysis and clustering reveal differences in motion and nanodomain patterns under normal conditions; however, the reviewer wonders why this comparison is not made in immune-activated conditions. Additionally, the reviewer could not understand the mechanisms or even just a functional relationship between this simple MSD and the Hir2 protein function in immunity, such as the compromised ROS or any other observed immune attenuations.
6. The authors should explain why HIR2Δ6 appears cytoplasmic in imaging but remains in the membrane fraction in Western blots in Figure 5.
7. For Fig. 6f, clarify if the protein is from the membrane fraction only. If so, does this indicate that the monomer stays membrane-bound, which is hard to understand, or is HIR2Δ6 oligomer cleared by degradation (e.g., 26S proteasome or autophagy), which needs to be tested? Alphafold prediction will provide a ring structure for many multimer versions with structural similarity to the database. Such data are good for in-house prediction and hypothesis generation, but are not appropriate for publication in a high-impact journal.

Referee #1:

The submitted manuscript by Weber and Ehinger et al. focuses on plant membrane organisation. To this end, the authors investigate plant-specific members of the stomatin/prohibitin/flotillin/HfIK/C (SPFH) family, HIR proteins, particularly the HIR2 isoform. They show that HIR2 interacts with several integral membrane proteins, such as BIR2 and 3, BAK1, CERK1 and others, and is anchored to the inner leaflet of the plasma membrane through a hydrophobic domain and lipidation. The authors then connect HIR2 to BAK1-mediated signalling pathways using hir2 mutant plants. Using super-resolution microscopy, they show that HIR2 affects receptor kinase dynamics and clustering. Structural modelling using AlphaFold3, corroborated by blue native PAGE experiments, provides evidence of higher-order assemblies, suggesting the existence of a novel structural framework for spatiotemporal membrane organisation. The manuscript is timely and reads very well. The experimental results mostly appear well-performed with corresponding controls. The manuscript will appeal to researchers interested in membrane organisation, membrane signalling, plant immunity and plant developmental biology. Nevertheless, I still have several suggestions and comments regarding the manuscript.

Major concerns
1)The authors state (page 7, lines 181 to 188), "The hir2 mutant phenotypes resemble those of robhD mutants. However, the accumulation of RbohD is not affected in hir2-2 or hir2-5 mutants, showing that the effect observed in hir2 mutants is not due to reduced RbohD levels." However, the authors show that the same is true for BAK1 (page 8, lines 227 to 228, Figure S7), and they discuss that the loss of HIR2 affects the membrane dynamics and organisation of BAK1. Are RBOHD dynamics and clustering affected in hir2 mutants?

Response to Referee #1

We thank Referee #1 for the careful evaluation and constructive suggestions.

Major concerns

1-1: We agree that the relationship between HIR2 and RBOHD is of particular interest given the similar phenotypes observed in the respective mutants. In the previous version of the ms, we showed that RBOHD protein abundance is unchanged in *hir2* mutants, suggesting that reduced ROS production in *hir2* mutants is not due to altered accumulation of RBOHD.

To strengthen the mechanistic link, we tested whether HIR2 interacts with RBOHD and could confirm an interaction between HIR2 and this key component of the ROS pathway (New Figure 1E). These findings suggest that HIR2 might influence ROS production through interaction with RBOHD and its role in membrane organization, rather than through effects on RBOHD stability or accumulation.

To support this model, we tested the interaction of HIR2 with BIK1 and confirmed their association (New Figure 1F). Since BIK1 and RBOHD are both key components of PAMP-induced ROS signaling downstream of receptors such as FLS2, these findings support the idea that HIR2 may contribute to the spatial organization of these complexes at the plasma membrane.

As a control for interaction specificity, we tested a membrane-associated NPH3 variant, which did not interact with HIR2 (New Figures 1 E, F; S1E). Thus, while HIR2 can associate with multiple plasma membrane proteins, its interactions are selective rather than non-specific. Together this shows that HIR2 can interact with multiple membrane proteins including many FLS2 complex components

pointing to a scaffold function where specificity is not determined by interaction alone. We added the following sentences for more clarity in line 150ff:

This shows that HIR2 is associated with multiple, but not all, plasma membrane proteins, indicating that HIR2 is a plasma membrane protein that can be found in proximity to various plasma membrane proteins that are not restricted to immune-related RKs. Specificity does not appear to be determined by interaction interfaces alone. Additional specificity may arise from post-translational modifications, ligand-induced complex dynamics, or the formation of hetero-oligomeric complexes.

2)How do the dynamics and clustering of BAK1 in Col-O and hir2 mutants change upon signalling, e.g., after Flg22 application?

1-2: The mobility of BAK1 is already reduced in the *hir2* mutants under basal conditions. As flg22 responses are only moderately affected in these mutants and flg22-induced changes in BAK1 nanoscale mobility are relatively subtle, detecting additional treatment-dependent effects on an already reduced mobility background proved challenging.

Although weak flg22-dependent mobility changes were observed in some experiments, these effects were not sufficiently robust to allow reliable statistical evaluation. Thus, it is, as expected, not easy to prove a statistically significant alteration of flg22-inducible mobility alterations. Therefore, we decided to not include these data in the ms. Instead, we shared the existing knowledge and obstacles with the readers in lines 243ff:

Flg22 treatment reduced the mobility of BAK1-mEos constructs in Col-0. In hir2 mutants, this flg22-induced response was still detectable; however, as basal mobility was already reduced, the additional effect of flg22 was less pronounced and therefore difficult to resolve. The overall flg22 effect on BAK1 mobility in Col-0 is relatively modest, and the impact of the hir2 mutation on flg22-induced responses is likewise subtle. Consequently, no reliable effects could be detected, suggesting that the sensitivity of this assay is insufficient to resolve the contribution of HIR2 to flg22-induced changes in BAk1 receptor kinase mobility (data not shown).

However, we tested whether flg22 treatment alters the nanoscale behaviour of HIR2 to determine whether HIR2 itself responds dynamically to immune activation. Using sptPALM analysis, we quantified HIR2 mobility and clustering properties following flg22 treatment and observed increased HIR2 mobility together with the formation of larger nanoclusters. These results demonstrate that HIR2 undergoes stimulus-dependent nanoscale reorganization in response to flg22 treatment and support the idea that HIR2 may contribute to dynamic adjustments of the plasma membrane environment during PAMP-triggered signalling.

3)The authors observed that the BAK1 diffusion coefficient is significantly lower in the hir2 mutants. If HIR2 restricts BAK1 in the membrane nanodomains, I would expect the diffusion coefficient to increase in the hir2 mutants.

1-3: We initially anticipated an increase in BAK1 mobility upon loss of HIR2 as well. However, our measurements consistently showed that BAK1 nanoscale mobility is reduced in *hir2* mutants. One possible explanation is that, in the absence of HIR2-mediated membrane organization, BAK1 engages more frequently or persistently with less mobile membrane-associated components, such as BIR proteins, thereby constraining its lateral diffusion. Alternatively, the loss of HIR2 may alter the biophysical properties of the plasma membrane, creating an environment that restricts BAK1 mobility compared with wild-type Col-0 plants.

Both scenarios are consistent with a role of HIR2 in regulating membrane organization and domain architecture, including geometry, cluster stability, and diffusion regimes. This interpretation is supported by alteration of membrane order in *hir2* mutants (New Figure 4G)

4)The authors identified the HIR proteins using co-immunoprecipitation with BIR2 and BIR3, followed by mass spectrometry analysis. The results are then presented in Table S1. However, only the HIR proteins are shown. Did the authors also identify other proteins?

1-4: Yes, we identified several additional proteins in the co-immunoprecipitation/mass spectrometry analyses. Parts of the raw dataset were previously published in (Halter 2017; doi.org/10.15496/publikation-15912; BIR2-interacting proteins) and Schulze et al. 2022 (BIR3-interacting proteins) and are publicly available via the PRIDE repository under accession PXD036942. We added this information in the methods section. However, we believe that discussion of additional interactors at this stage would not substantially strengthen the central conclusions of the current study and comprehensive characterization of additional candidate proteins would be beyond the scope of the present manuscript.

In Table S1, HIR2 (Uniprot ID Q9CAR7) does not contain cysteines at positions 6 and 7, unlike HIR3 (Uniprot ID Q9SRH6).

Yes, there is an ongoing nomenclature inconsistency that persists despite our previous communication with UniProt regarding this issue. In the original version of the manuscript, we used the UniProt annotations as provided in the mass spectrometry output files. To ensure unambiguous identification of the corresponding genes, we have now revised the table to include TAIR annotations and AGI identifiers throughout (revised Table S1).

5) Figure 3b contains two graphs showing the total ROS production. Are there two independent experiments?

1-5: Yes, Figure 3B represent two independent experiments. One experiment included a single CRISPR line together with the additional mutant lines, whereas the second experiment included both independent CRISPR lines (only total RLU shown in Figure 3B). The full experiment is visible in Figure S3A,B.

Minor comments

1) It might be better to specify the split-ubiquitin yeast two-hybrid assay (page 5, line 138) to avoid confusion with the traditional Y2H assay.

We revised the terminology to explicitly distinguish split-ubiquitin assays from classical yeast two-hybrid assays in line 143.

On line 139, the reference should be to Figure S1e, not to Figure 1e. Similarly, on line 143, it should be to Figure S1f, not Figure 1f.

Also, on page 7, line 181, it should not be to Figure 5a-d, but Figure S5.

We thank the reviewer for the detailed examination, which helped identify these mistakes. We apologize for the mislabelling, have carefully checked all cross-references, and corrected them accordingly.

2)There is no reference to FirstGlance.

We added the reference to FirstGlance in line 284. Adding the link is the officially requested method of citation by the creator.

3)Page 11, line 316, a new type of membrane compartment might be an overstatement.

We agree with this comment and phrased the sentence more carefully, see line 357ff:

The high molecular weight complexes of HIR2 are hypothesized to form a ring structure, which would represent a new type of membrane sub-compartment in plants that offers a novel perspective on the organization of membrane domains and the formation of signaling platforms in plant membranes.

We believe that
4)On page 18, line 527, the W5 solution is not defined.

We apologize for this missing information and added the following information in line 605f:

(2 mM MES, 154 mM NaCl, 125 mM $CaCl_2$, 5 mM KCl; pH 5.7)

We appreciate the detailed, positive, and constructive comments and sincerely apologize for the mislabeling and missing information.

Referee #2:

This is a very well-written paper that highlighted the functions of HIR2 protein in plant growth and immune responses. I commend the authors on the discoveries made, as well as multiple-prong approaches taken to demonstrate the functions of HIR2 in both plant growth and immunity. HIR2 belongs to a group of SPFH family that have been shown to influence membrane organization in Arabidopsis. The authors showed solid evidence that HIR2 interacted with multiple plasma membrane proteins, including major common co-receptors such as BAK1 and BIRs. The authors also reported the multimeric state of HIR2 via AlphaFold prediction, suggesting that HIR2 could serve as a spatial platform for membrane organization of membrane-occupying receptors.

Response to Referee #2

We thank the reviewer for the positive assessment of our study and for the constructive suggestions, which have helped improve clarity and strengthen the mechanistic interpretation of our findings.

There are a number of minor points I think the authors may want to address to improve the quality of this manuscript:
1) Line 145: The authors wrote "Specificity is not determined by interaction interfaces here". The authors may want to clarify this: did they mean the interaction interfaces as in nanodomains vs. non-nanodomains?

2-1: What we meant here is that interaction of HIR2 alone is not conferring specificity, as it can interact with multiple membrane proteins. We added a little more info here to clarify the sentence in line 153ff:

Specificity does not appear to be determined by interaction interfaces alone. Additional specificity may arise from post-translational modifications, ligand-induced complex dynamics, or the formation of hetero-oligomeric complexes.

2) Line 196-197: The authors should compare the quantitative contribution of HIR2 to disease resistance against P. syringae with other mutants in the HIR family (HIR1, HIR 3, HIR4) to conclude that "HIR2 is required to establish plant immune responses quantitatively"

2-2: We agree that the information we provided on the results section alone is not sufficient to understand the redundancy in HIRs involved in plant defense. We cited the relevant literature in the introduction line 88f :

Multiple studies link HIRs to plant immunity and cell death (Choi et al, 2011; Jung & Hwang, 2007; Jung et al, 2008; Li et al, 2019; Liu et al, 2024; Lv et al, 2017; Qi & Katagiri, 2012; Qi et al., 2011; Qi & Katagiri, 2009; Zhou et al, 2010; Zhou et al, 2009), …

We discussed the role of HIR2 later in the discussion in line 381ff, where we cited literature showing that HIR proteins are involved in plant defense, including the study by Liu et al, (2024). This work demonstrated that *hir2* mutants are weakly affected in immune responses, whereas *hir2 hir4* double mutants show stronger phenotypes, including decreased MAPkinase activation and increased susceptibility to the bacterial pathogen *Pto* DC3000. The study further describes enhanced susceptibility to *Pto* DC3000 in mutants of all 4 Arabidopsis HIR genes.

Line 176ff : *The hir2-2 mutant, in particular, exhibits enhanced bacterial and fungal growth (Liu et al., 2024; Qi et al., 2011), suggesting a role as a positive regulator of plant immunity. The phenotypes of hir4 mutants were enhanced when combined with hir2 CRISPR/Cas9 alleles, supporting that weak hir2 null mutant phenotypes are underestimated by functional redundancy with other family members (Liu et al., 2024).*

Line 196ff: *To analyze the effects of HIR2 on disease resistance, we infected hir2-2, hir2-5, and hir2 cr mutants with Pseudomonas syringae pathovar tomato DC3000 (PtoDC3000). The average bacterial growth in hir2-2 was up to 20-fold higher than in Col-0 (Figure 3E). Bacterial growth was also increased in hir2-5 and hir2 cr33, but not always statistically significant. Considering the partial redundancy within the HIR family, these data indicate that HIR2 contributes quantitatively to the establishment of plant immune responses (Liu et al., 2024).*

Line 382ff: Together with previous reports linking HIR proteins to resistance against bacterial, fungal, and viral pathogens, HIR2 is placed within the plant immune machinery (Choi et al., 2011; Danek et al, 2026; Jung & Hwang, 2007; Jung et al., 2008; Li et al., 2019; Liu et al., 2024; Lv et al., 2017; Mei et al, 2020; Qi & Katagiri, 2012; Qi et al., 2011; Qi & Katagiri, 2009; Zhou et al., 2010; Zhou et al., 2009). The stronger phenotypes observed in double hir2 hir4 mutants in bacterial infection assays further support functional redundancy within the HIR family (Liu et al., 2024).

In this study, we included *hir2-2, hir2-5,* and a *hir2 cr* allel in the bacterial growth assays (Figure 3E), and we have now included the citation Liu et al. (2024) in line 201f.

3) BAK1 dynamics being affected by HIR2: How does BAK1 diffusion coefficient changed after flg22 elicitation? Does the authors have any date to show that HIR2 influence BAK1 dynamics once ligand recognition happens using sptPALM? This is essential to connect BAK1 dynamics to its signaling and downstream responses.

2-3: As already addressed in response to Reviewer 1 (point 2), we analyzed flg22-induced changes in BAK1 mobility using sptPALM. However, because BAK1 mobility is already reduced in *hir2* mutants

under basal conditions, resolving additional flg22-dependent changes proved challenging, In wild-type plants, BAK1 mobility is reduced after flg22 treatment; however, in *hir2* mutants, where mobility is alreadyreduced , it is difficult to determine whether flg22 induces a further decrease or whether mobility has already reached a lower limit.

We observed a trend towards lower BAK1 mobility upon flg22 treatment; however, this effect was not consistently significant across independent experiments. Given the modest alteration in Col-0 and the altered baseline state in *hir2* mutants, the current assay does not provide sufficient resolution to reliably quantify flg22-dependent changes in this background.

We therefore decided not to include these data in the manuscript. Nonetheless, across experiments BAK1 mobility remains consistently reduced in *hir2* mutants compared to wild type. As all reviewers asked this, we added an explanation to the manuscript explaining why this information is unavailable (line 243ff):

Flg22 treatment reduced the mobility of BAK1-mEos constructs in Col-0. In hir2 mutants, this flg22-induced response was still detectable; however, as basal mobility was already reduced, the additional effect of flg22 was less pronounced and therefore difficult to resolve. The overall flg22 effect on BAK1 mobility in Col-0 is relatively modest, and the impact of the hir2 mutation on flg22-induced responses is likewise subtle. Consequently, no reliable effects could be detected, suggesting that the sensitivity of this assay is insufficient to resolve the contribution of HIR2 to flg22-induced changes in receptor mobility (data not shown).

We additionally assessed the effect of flg22 on the nanoscale dynamics of HIR2 using sptPALM. Upon flg22 treatment, HIR2 showed a significant increase in lateral mobility together with an increase in nanocluster size. These results indicate that HIR2 is responsive to PAMP stimulation and undergoes dynamic reorganisation at the plasma membrane, consistent with the formation of larger HIR2-containing assemblies that may facilitate the spatial coordination of receptor kinase complexes (New Figure 4B-D).

4) Fig 1e: there is an issue with the input of lane # 2 (only CERK1-HA and no HIR2-GFP). The figure showed a band positive for HIR2-GFP and some residual signal in the IP: α-GFP blot when probed with α-GFP.

2-4: We fully agree that this was a suboptimal blot. The additional bands are caused by spill-over from the adjacent lanes but did not affect the result. But as we replaced the blot with interaction assays with BIK1 and RBOHD, it will no longer be present in the manuscript.

5) Line 327-330: The authors should elaborate their speculation of how the multimeric structure of HIR2 complexes could facilitate its role as a scaffolding protein for RKs, promoting RK-mediated responses.

2-5: We modified the conclusion to clarify the model of HIR function (line 498ff):

Integrating all results, we propose that HIR2 contributes to the regulation of plasma membrane organization, thereby influencing receptor mobility and signalling output. HIR2 is incorporated into the plasma membrane and dynamically modulates its nanoscale environment. It assembles into higher-order oligomeric complexes that may contribute to the formation of distinct membrane nanodomains, which in turn could provide a structural context supporting the assembly and function of signalling platforms at the cell surface.

6) The authors should also elaborate on the <u>experimental details of the AlphaFold</u> prediction and

annotate the domain in the protein structure clearly in the result section (e.g., amino acid position of the N-terminus, SPFH domain, the three helices, etc.).

2-6: We thank the reviewer for this constructive comment, which helped improve the clarity of the manuscript. We have now added a more detailed description in the Methods section outlining how the AlphaFold modelling was performed, including the input sequences, multimer prediction strategy, confidence metrics, and filtering of models based on consistency across different seeds.

We have further extended the AlphaFold 3 analysis by performing protomer screening to evaluate whether higher-order HIR2 assemblies, as observed by our blue native data, can be reliably modelled. While AlphaFold 3 was able to generate consistent models for lower-order assemblies, we observed that predictions of larger oligomeric states increasingly resulted in structural superposition and reduced confidence scores. This limitation is consistent with previous reports describing decreased accuracy of AlphaFold-based predictions for higher-order complexes and suggests that these models should be interpreted cautiously (Toghani et al. 2026). We added the following information in line 716ff:

Fasta sequences of HIR2 were retrieved from Aramemnon and introduced into the Web-based AlphaFold server. Homo and heterooligomeric complexes with HIR1, 3 and 4 were modelled. Seed variations were tested to test the reliability of the modelling. The best of 5 models was used for further analysis of plDDT confidence scores and pTM and ipTM values were used for reliability evaluation of the intra- and intermolecular interactions, respectively. The predicted aligned error (PAE) was checked for the mono and oligomeric predictions.

We have annotated the predicted structural features in a new Figure S7A to facilitate interpretation of the AlphaFold models. This figure maps the domain architecture, secondary structure elements, and residue positions onto the predicted HIR2 structure, enabling direct comparison between sequence-based domain predictions and the structural models.

We thank the reviewer for the detailed, constructive, and positive feedback.

Referee #3:

Hannah Weber and co-authors described a study of HIR2 function in plant immunity with biochemistry, cell biology, and structural predictions. HIR2 is anchored to the inner leaflet of the plasma membrane in a nanodomain-like structure via a hydrophobic domain and S-acylation. HIR2 was found to interact with BIRs, which are leucine-rich repeat receptor kinases, as negative regulators of BAK1 complex formation and signaling. This research area is highly promising for understanding how surface biomolecular compartmentalization regulates molecular dynamics and function during immune responses. However, the experimental results do not clearly connect the observed imaging patterns of HIR2 and BIR3 to their functional roles in immunity. Additionally, immune defense assays in HIR2 mutant and overexpression lines were not conducted, limiting the integration of this information. The reviewer finds the work interesting but very preliminary, and cannot recommend at this stage.

Response to Referee #3

We thank the reviewer for the positive assessment of the topic's relevance and for the critical comments, which helped us improve and strengthen the manuscript.

We would like to clarify that immune defence assays are already included in the manuscript (Fig. 3e), where *Pseudomonas syringae* infection experiments demonstrate altered bacterial growth in *hir2*

mutants (Figure 3E). In addition, these data are complemented by analyses of ROS production, interaction with the defense-related proteins RBOHD and BIK1 (New Figures 1 E and F), as well as BAK1 and FLS2, together with the observed flg22-induced changes in HIR2 nanoscale dynamics (New Figure 4B-D) (see also response to Reviewer 1 comment 2, and Reviewer 2 comment 3). These findings, together with previously published studies from other groups (see line 381ff), provide multiple layers of functional evidence linking HIR2 to immune signalling outputs.

We agree with the reviewers' comment that integrating molecular mechanisms with immune function is an important aspect. We therefore revised and expanded the Discussion accordingly and hope that the additional data and improved interpretation clarify the role of HIR2 in plant defence and plasma membrane organization. See also comment to reviewer 2 comment 2 and more details in comment 3-3.

1. The FRET-FLIM data show HIR2 interacts with all tested proteins, leading the authors to conclude that HIR2 is abundant. This conclusion seems illogical. If this abundance conclusion is meaningful, a control with an unrelated, highly expressed membrane protein (e.g., Signal peptide-Transmembrane domain-GFP) should be included. Also, the citation should refer to Figure S1f, not Figure 1f. Because in most of the case membrane associated protein will have positive FLIM-FRET results due to close distance by overexpression in tobacco.

3-1: We agree with the reviewer's comment and have removed the term "abundant" to avoid any potential misinterpretation. We added the following sentences to clarify our interpretation of the interactions line 150ff:

This shows that HIR2 is associated with multiple, but not all, plasma membrane proteins, indicating that HIR2 is a plasma membrane protein that can be found in proximity to various plasma membrane proteins that are not restricted to immune-related RKs. Specificity does not appear to be determined by interaction interfaces alone. Additional specificity may arise from post-translational modifications, ligand-induced complex dynamics, or the formation of hetero-oligomeric complexes.

We sincerely apologise for the mislabelling of the figure and have corrected it accordingly.

However, we respectfully disagree with the implication that most membrane proteins yield positive FLIM-FRET interactions under transient expression in *Nicotiana benthamiana*. One example supporting this is the work by Holzwart et al. (2018; https://doi.org/10.1073/pnas.181443411), which demonstrates that FLIM-FRET signals depend on specific interaction contexts (RLP44-PSKR1), rather than being a general consequence of membrane co-localisation or overexpression (e,g, no interaction between FLS2 and RLP44).

Importantly, our conclusions are not based solely on FLIM-FRET measurements. We have additionally validated the interactions using co-immunoprecipitation and split-ubiquitin assays, providing independent biochemical evidence for complex formation. Furthermore, we extended the analysis to homologous systems, including *Arabidopsis* protoplasts, which support the observed interaction patterns under more native conditions.

To further strengthen the specificity of our interaction dataset, we have now included NPH3 as a negative control in the interaction assays. This demonstrates that HIR2 does not promiscuously interact with all membrane-associated proteins but rather shows selectivity towards a subset of membrane proteins.

2. The reviewer cannot discern enhanced growth in HIR2 overexpression lines (HIR2oe) in Figure S3, as claimed by the authors.

3-2: We agree with the reviewer's comment and apologise for the overinterpretation. We had referred to previously generated overexpression lines and have since realised that some of these lines show HIR2 silencing and potentially secondary effects on related genes, which likely confounded the original interpretation.

We have since generated new stable HIR2 overexpression lines driven by the Ubi1-promoter, which do not exhibit growth defects, and we have removed the previous statement accordingly. To provide transparency, we included expression analyses for the HIR2 overexpression lines in new Figure S3E.

Importantly, the ROS burst data shown in Figure S4C, D remain valid, as all lines used in these assays were confirmed for HIR2 expression after experimentation.

3. The authors cite another paper stating that the hir2-2 mutant shows increased bacterial and fungal growth, suggesting HIR2 is a positive regulator of immunity. However, hir2-2 has an additional T-DNA insertion, complicating the background.

3-3: We performed whole-genome sequencing of the *hir2-2* mutant and did not detect an additional T-DNA insertion as previously reported (line 171f). We therefore consider that the observed phenotype is not caused by a second insertion event.

One possible explanation for the relatively strong phenotype of *hir2-2* compared with other single mutants is a dominant-negative effect of residual HIR2 transcript or protein fragments. Consistent with a requirement for combined HIR2/ HIR4 function in plant immunity, Liu et al. (2024) reported that *hir2 hir4* double mutants display markedly enhanced immune-related phenotypes, resembling those observed in *hir2-2*.

The reviewer questions why infection assays were not performed with the samples used in this study (e.g., 2-5, cr, and two OE lines).

We included *hir2-2*, *hir2-5*, and the CRISPR null allele *hir2 cr33* in the *Pto* DC3000 infection assays (Fig. 3e). All lines showed enhanced bacterial growth, although the effect was not consistently statistically significant in *hir2-5* and *hir2 cr33*. We attribute the comparatively weak and variable phenotype to functional redundancy within the HIR protein family. In support of this interpretation, Liu et al. (2024; Fig. 4, S6, S11) demonstrated that *hir2*, *hir4*, and *hir2 hir4* double mutants all display significantly increased susceptibility to *Pto* DC3000, indicating partially overlapping and redundant contributions of HIR family members to immunity.

We agree that this aspect was not sufficiently explained in the Results section and have now added the relevant citation for clarity. We would prefer to address the implications of HIR redundancy in more detail in the Discussion section (line 381ff).

4. While the hir2 mutants do not affect MAPK or ethylene production, the cause of compromised peptide-triggered ROS remains unclear. The discussion on RbohD and the hir2 mutant is interesting but lacks mechanistic investigation and insights into how PTI-triggered ROS is reduced, whether through RbohD, receptor interactions, or ligand binding in hir2 mutants.

3-4: In *hir2 hir4* double mutants, MAPkinase activation is also affected after chitin treatment (Liu et al. 2024). This indicates that signaling defects are not restricted to ROS production, but weak changes in single mutants may be below the detection sensitivity of respective assays.

We agree that the connection to RBOHD is interesting and have therefore extended our analysis to include interaction assays of HIR2 with RBOHD and the upstream kinase BIK1. Both RBOHD and BIK1 interact with HIR2, supporting the idea that HIR2 associates with components of the FLS2 receptor complex and downstream components and thereby influences PAMP-induced ROS production and signaling. We propose that this mechanism is based on the membrane-organizing function of HIR2, supported by an observed alteration of HIR2 mobility and clustering during PAMP induced immunity (New Figure 4B-D).

5. Superresolution imaging of HIR2 and BIR3 was conducted, but without comparing PAMP-activated and unactivated samples. The MSD analysis and clustering reveal differences in motion and nanodomain patterns under normal conditions; however, the reviewer wonders why this comparison is not made in immune-activated conditions.

3-5: We performed these assays; however, the already reduced mobility of BAK1 in *hir2* mutants, together with the weak immune phenotypes of the single mutants, prevented statistically robust and reliable conclusions. See also the response to Reviewer 1 comment 2 and Reviewer 2 comment 3

To clarify this, we added a paragraph explaining this outcome in line 243ff:

Flg22 treatment reduced the mobility of BAK1-mEos constructs in Col-0. In hir2 mutants, this flg22-induced response was still detectable; however, as basal mobility was already reduced, the additional effect of flg22 was less pronounced and therefore difficult to resolve. The overall flg22 effect on BAK1 mobility in Col-0 is relatively modest, and the impact of the hir2 mutation on flg22-induced responses is likewise subtle. Consequently, no reliable effects could be detected, suggesting that the sensitivity of this assay is insufficient to resolve the contribution of HIR2 to flg22-induced changes in receptor mobility (data not shown).

Additionally, the reviewer could not understand the mechanisms or even just a functional relationship between this simple MSD and the Hir2 protein function in immunity, such as the compromised ROS or any other observed immune attenuations.

Across plant and animal receptor systems, signaling output is tightly linked to the nanoscale mobility and spatial organization of plasma membrane receptors, rather than receptor abundance alone. In plants, the RLK FERONIA (FER) regulates immune receptor complexes such as FLS2 and its co-receptor BAK1 by controlling their partitioning into nanodomains and their lateral diffusion. FER-dependent signaling via RALF peptides rapidly alters receptor mobility and clustering, which in turn modulates downstream immune responses, establishing a functional link between membrane dynamics and signaling output (Gronnier et al 2022). Importantly, this work demonstrates that perturbation of FER signaling alters both receptor nanoscale organization and immune signaling strength, supporting a causal role for membrane organization in regulating signaling competence rather than a purely correlative association.

We show that loss of HIR2 alters the lateral mobility of the co-receptor BAK1, and that HIR2 itself is dynamically reorganized upon flg22 perception. Consistently, *hir2* mutants display impaired immune responses, supporting a role for HIR2 in immune signalling, potentially by regulating BAK1 receptor kinase nanodomain dynamics and function

Our data provide strong evidence that HIR2 contributes to the biophysical dynamics of membrane proteins and the organization of plasma membrane environments and dynamically responds to flg22 treatment, thereby enabling effective immune signaling. However, a complete mechanistic characterization of this pathway is currently constrained by the experimental limitations outlined in comment 5 and is, in our view, beyond the scope of the present manuscript.

6. The authors should explain why HIR2Δ6 appears cytoplasmic in imaging but remains in the membrane fraction in Western blots in Figure 5.

3-6: A substantial fraction of HIR2ΔN6 is found in the soluble fraction, indicating reduced membrane association. Because the membrane fraction includes both plasma membrane and endomembranes, fractionation alone does not resolve subcellular membrane localization. However, confocal imaging clearly shows a loss of plasma membrane localization, indicating that HIR2ΔN6 predominantly accumulates in endomembrane compartments rather than at the plasma membrane.

7. For Fig. 6f, clarify if the protein is from the membrane fraction only. If so, does this indicate that the monomer stays membrane-bound, which is hard to understand, or is HIR2Δ6 oligomer cleared by degradation (e.g., 26S proteasome or autophagy), which needs to be tested?

3-7: The samples shown in Figure 6F correspond to total protein extracts, as now clarified in line 322. Based on the subcellular localization analysis presented in Figure 5, HIR2ΔN6 is predominantly detected in the soluble and endomembrane fractions, consistent with altered plasma membrane association.

To assess whether the reduced abundance of HIR2ΔN6 is due to proteasomal degradation, we treated samples with proteasome inhibitors. Under these conditions, we did not observe increased accumulation of HIR2ΔN6 (data available on request), suggesting that the proteasome is unlikely to be the cause of the loss of higher-order oligomeric complexes.

In samples expressing sufficient levels of HIR2ΔN6, monomeric species are clearly detectable (Figure 6F). Together, these observations are consistent with a model in which oligomeric forms of HIR2ΔN6 are selectively not formed or destabilized, while monomeric protein remains stable.

Alphafold prediction will provide a ring structure for many multimer versions with structural similarity to the database. Such data are good for in-house prediction and hypothesis generation, but are not appropriate for publication in a high-impact journal.

We agree that AlphaFold-based modelling alone is insufficient to derive definitive structural conclusions regarding native protein assemblies or higher-order complexes. In this study, we therefore used the modelling primarily for hypothesis generation and experimentally tested these predictions using Blue Native PAGE analysis. We could show that HIR2 form high-molecular-weight oligomeric complexes, consistent with oligomerization observed for other SFFH-domain proteins in bacterial and animal systems (Fu & MacKinnon, 2024; Ma *et al.*, 2022; Qiao *et al.*, 2022; Tan *et al.*, 2024).

Importantly, SPFH-family members have been experimentally shown to form higher-order assemblies, including ring- or cage-like structures, supporting conserved principles of membrane-associated oligomerization (Fu & MacKinnon, 2024; Ma et al., 2022; Qiao et al., 2022; Tan et al., 2024). The HIR2 model is consistent with both (i) experimentally validated architectures of related SPFH proteins and (ii) our biochemical data, we consider the predicted structure as plausible

structural HIR2 organization at the plasma membrane, rather than an definitive molecular model. Definitive validation of the exact supramolecular arrangement will require future high-resolution structural approaches.

To further clarify the limitations of multimer prediction, we included a protomer screening analysis (New Figure S7B). With increasing protomer numbers AlphaFold 3 generated superimpositions that are impossible to occur in nature, thereby illustrating the inherent limitations of the modelling approach.

Although the precise structural organization of HIR2 remains to be resolved, our combined biochemical and modelling data support the presence of large oligomeric assemblies at the plasma membrane, providing a framework stimulating future mechanistic studies of HIR-mediated membrane organization.

We thank the reviewer for the critical evaluation and constructive comments, and we hope that we have been able to convey that this work represents a valuable contribution to advancing our understanding of HIR proteins and plasma membrane organization.

1st Revision - Editorial Decision 16th Jun 2026

Dear Birgit,

We have now received re-review reports from two referees, which I have included below. As you will see, you have addressed their concerns satisfactorily. Before I can finally accept the manuscript, there are some remaining editorial points which need to be addressed. In this regard would you please:

- rename the conflict of interest statement as the 'Disclosure and competing interests statement',
- declare any employment in a biotech company in this statement,
- remove the AC/CrediT section from the manuscript text,
- remove the reference to 'data not shown',
- correct the manuscript callout for Table S3/Supplemental Table S3; no such table has been uploaded,
- upload figures as individual, high-resolution Figure files,
- the title page of the appendix should contain "Appendix for Arabidopsis HYPERSENSITIVE INDUCED REACTION 2 modulates receptor pathways and membrane organization" (the author and affiliation list is not necessary here), and a table of contents with the page numbers for the listed items; nomenclature should be Appendix Figure Sx and Appendix Table Sx throughout the main manuscript and Appendix PDF,
- state use of the same images (between Figure 1 and Figure EV1 [as well as within Figure EV1]) in the legend of Figure EV1 and provide corresponding unprocessed images as Source Data files,
- define box plots in terms of minima, maxima, centre, bounds of box and whiskers, and percentile in the legend of figures 1B,D,
- in the main manuscript text, legends for figure 1 need to be provided sequentially (legend for figure 1C is currently provided before legend of figure 1B),
- provide exact p values in the legends of 1B,D; 2B; 3B,D,E; 4A,B,C,E,F,G,
- remove "Supplementary information" from the main manuscript file,
- provide suggestions for a short 'blurb' text prefacing and summing up the conceptual aspect of the study in two sentences (max. 250 characters), followed by 3-5 one-sentence 'bullet points' with brief factual statements of key results of the paper; they will form the basis of an editor-written 'Synopsis' accompanying the online version of the article, and
- use the following section order: Title page - Abstract - Introduction - Results - Discussion - Methods - Data Availability - Acknowledgements - Disclosure and Competing Interests Statement - References - Figure Legends - Table(s) - Expanded View Figure Legends.

We include a synopsis of the paper (see http://emboj.embopress.org/), this image should be in .jpg or .png format, exactly 550 pixels wide and 300 pixels high. Please ensure that the text is legible at the final size and that the synopsis image has these proportions,

I am looking forward to receiving your revised manuscript.

EMBO Press is an editorially independent publishing platform for the development of EMBO scientific publications.

Best wishes,

William

William Teale, PhD
Editor
The EMBO Journal
w.teale@embojournal.org

Instructions for preparing your revised manuscript:

Read our guidance for manuscript revisions and related editorial policies: https://link.springer.com/journal/44318/submission-guidelines#cms-Revised-submissions

Please also check that the title and abstract of the manuscript are brief, yet explicit, even to non-specialists.

When assembling figures, please refer to our figure preparation guideline in order to ensure proper formatting and readability in print as well as on screen:
https://media.springernature.com/original/springer-cms/rest/v1/content/27825798/data/v1

IMPORTANT: When you send the revision we will require

- a point-by-point response to the referees' comments, with a detailed description of the changes made (as a word file).
- a word file of the manuscript text.
- individual production quality figure files (one file per figure)
- a complete author checklist
- Expanded View files (replacing Supplementary Information)
- a Reagents and Tools Table as part of the Methods section

Please remember: Digital image enhancement is acceptable practice, as long as it accurately represents the original data and conforms to community standards. If a figure has been subjected to significant electronic manipulation, this must be noted in the figure legend or in the 'Methods' section. The editors reserve the right to request original versions of figures and the original images that were used to assemble the figure.

We realize that it is difficult to revise to a specific deadline. In the interest of protecting the conceptual advance provided by the work, we recommend a revision within 3 months (14th Sep 2026). Please discuss the revision progress ahead of this time with the editor if you require more time to complete the revisions. Use the link below to submit your revision:

https://emboj.msubmit.net/cgi-bin/main.plex

Referee #1:

I would like to thank the authors for addressing my comments. I have no further concerns or suggestions.

Referee #3:

The revised manuscript has substantially improved in quality, accuracy, and clarity. The authors have addressed the previous concerns by correcting overstatements and resolving issues related to data interpretation. I also find their responses to the reviewers' comments convincing, with most key arguments now supported by additional experimental evidence.
Overall, the manuscript is of high quality now and provides important insights into the field of nanoscale signaling complexes. I believe it will make a significant contribution to the field and am supportive of its publication in its current form.

Response letter

Dear William,

We appreciate that the reviewers are satisfied with our revisions.

Sincerely,
Birgit Kemmerling
(On behalf of all co-authors)

Referee #1:

I would like to thank the authors for addressing my comments. I have no further concerns or suggestions.

Referee #3:

The revised manuscript has substantially improved in quality, accuracy, and clarity. The authors have addressed the previous concerns by correcting overstatements and resolving issues related to data interpretation. I also find their responses to the reviewers' comments convincing, with most key arguments now supported by additional experimental evidence. Overall, the manuscript is of high quality now and provides important insights into the field of nanoscale signaling complexes. I believe it will make a significant contribution to the field and am supportive of its publication in its current form.

2nd Revision - Editorial Decision 6th Jul 2026

Dear Birgit,

I am pleased to inform you that your manuscript has been accepted for publication in the EMBO Journal.

Congratulations to you and the team!

Your manuscript will be processed for publication by EMBO Press. It will be copy edited and you will receive page proofs prior to publication. Please note that you will be contacted by Springer Nature Author Services to complete licensing and payment information.

You may qualify for financial assistance for your publication charges - either via a Springer Nature fully open access agreement or an EMBO initiative. Check your eligibility: https://link.springer.com/journal/44318/how-to-publish-with-us

Should you be planning a Press Release on your article, please notify the production team via this form in order to coordinate publication and release dates: https://forms.cloud.microsoft/e/eH4A8JVP6g

If you have any questions, please do not hesitate to contact the Editorial Office. Thank you for your contribution to The EMBO Journal.

Best wishes,

William

William Teale, PhD
Editor
The EMBO Journal
w.teale@embojournal.org

*****
Please note that it is The EMBO Journal policy for the transcript of the editorial process (containing referee reports and your response letters) to be published as an online supplement to each paper. If you should prefer removal of any referee-only figures included in the point-by-point response(s), e.g. because they may still be used for future publication or because they have been reproduced from published work by others, please do let us know immediately via response email.
More information is available here: https://link.springer.com/partners/embo-press/editorial-policies#Peer%20review

** Click here to be directed to your login page: https://emboj.msubmit.net

EMBO Press Author Checklist

Corresponding Author Name: Birgit Kemmerling	
Journal Submitted to: The EMBO Journal	
Manuscript Number: EMBOJ-2025-121356	

USEFUL LINKS FOR COMPLETING THIS FORM
The EMBO Journal - Author Guidelines
EMBO Reports - Author Guidelines
Molecular Systems Biology - Author Guidelines
EMBO Molecular Medicine - Author Guidelines

Reporting Checklist for Life Science Articles (updated January
This checklist is adapted from Materials Design Analysis Reporting (MDAR) Checklist for Authors. MDAR establishes a minimum set of requirements in transparent reporting in the life sciences (see Statement of Task: 10.31222/osf.io/9sm4x). Please follow the journal's guidelines in preparing your
Please note that a copy of this checklist will be published alongside your article.

Abridged guidelines for figures
1. Data
The data shown in figures should satisfy the following conditions:

- the data were obtained and processed according to the field's best practice and are presented to reflect the results of the experiments in an accurate and unbiased manner.
- ideally, figure panels should include only measurements that are directly comparable to each other and obtained with the same assay.
- plots include clearly labeled error bars for independent experiments and sample sizes. Unless justified, error bars should not be shown for technical
- if n<5, the individual data points from each experiment should be plotted. Any statistical test employed should be justified.
- Source Data should be included to report the data underlying figures according to the guidelines set out in the authorship guidelines on Data

2. Captions
Each figure caption should contain the following information, for each panel where they are relevant:

- a specification of the experimental system investigated (eg cell line, species name).
- the assay(s) and method(s) used to carry out the reported observations and measurements.
- an explicit mention of the biological and chemical entity(ies) that are being measured.
- an explicit mention of the biological and chemical entity(ies) that are altered/varied/perturbed in a controlled manner.
- the exact sample size (n) for each experimental group/condition, given as a number, not a range;
- a description of the sample collection allowing the reader to understand whether the samples represent technical or biological replicates (including how many animals, litters, cultures, etc.).
- a statement of how many times the experiment shown was independently replicated in the laboratory.
- definitions of statistical methods and measures:
  - common tests, such as t-test (please specify whether paired vs. unpaired), simple χ2 tests, Wilcoxon and Mann-Whitney tests, can be unambiguously identified by name only, but more complex techniques should be described in the methods section;
  - are tests one-sided or two-sided?
  - are there adjustments for multiple comparisons?
  - exact statistical test results, e.g., P values = x but not P values < x;
  - definition of 'center values' as median or average;
  - definition of error bars as s.d. or s.e.m.

Please complete ALL of the questions below.
Select "Not Applicable" only when the requested information is not relevant for your study.

Materials

Newly Created Materials	Information included in the manuscript?	In which section is the information available? (Reagents and Tools Table, Materials and Methods, Figures, Data Availability Section)
New materials and reagents need to be available; do any restrictions apply?	Yes	Data availability section

Antibodies	Information included in the manuscript?	In which section is the information available? (Reagents and Tools Table, Materials and Methods, Figures, Data Availability Section)
For **antibodies** provide the following information:<br>- Commercial antibodies: RRID (if possible) or supplier name, catalogue number and or/clone number<br>- Non-commercial: RRID or citation	Yes	Reagents and Tools Table

DNA and RNA sequences	Information included in the manuscript?	In which section is the information available? (Reagents and Tools Table, Materials and Methods, Figures, Data Availability Section)
**Short novel DNA or RNA including primers, probes**: provide the sequences.	Yes	Appendix Table S2

Cell materials	Information included in the manuscript?	In which section is the information available? (Reagents and Tools Table, Materials and Methods, Figures, Data Availability Section)
**Cell lines:** Provide species information, strain. Provide accession number in repository **OR** supplier name, catalog number, clone number, and/**OR** RRID.	Not Applicable	
**Primary cultures:** Provide species, strain, sex of origin, genetic modification status.	Not Applicable	
Report if the cell lines were recently **authenticated** (e.g., by STR profiling) and tested for mycoplasma contamination.	Not Applicable	

Experimental animals	Information included in the manuscript?	In which section is the information available? (Reagents and Tools Table, Materials and Methods, Figures, Data Availability Section)
**Laboratory animals or Model organisms:** Provide species, strain, sex, age, genetic modification status. Provide accession number in repository **OR** supplier name, catalog number, clone number, **OR** RRID.	Not Applicable	
**Animal observed in or captured from the field:** Provide species, sex, and age where possible.	Not Applicable	
Please detail **housing and husbandry conditions**.	Not Applicable	

Plants and microbes	Information included in the manuscript?	In which section is the information available? (Reagents and Tools Table, Materials and Methods, Figures, Data Availability Section)
**Plants:** provide species and strain, ecotype and cultivar where relevant, unique accession number if available, and source (including location for collected wild specimens).	Yes	Reagents and Tools Table
**Microbes:** provide species and strain, unique accession number if available, and source.	Yes	Reagents and Tools Table

Human research participants	Information included in the manuscript?	In which section is the information available? (Reagents and Tools Table, Materials and Methods, Figures, Data Availability Section)
If collected and within the bounds of privacy constraints report on age, sex and gender or ethnicity for all study participants.	Not Applicable	

Core facilities	Information included in the manuscript?	In which section is the information available? (Reagents and Tools Table, Materials and Methods, Figures, Data Availability Section)
If your work benefited from core facilities, was their service mentioned in the acknowledgments section?	Yes	Acknowledgment

Design

Study protocol	Information included in the manuscript?	In which section is the information available? (Reagents and Tools Table, Materials and Methods, Figures, Data Availability Section)
If study protocol has been **pre-registered, provide DOI in the manuscript**. For clinical trials, provide the trial registration number **OR** cite DOI.	Not Applicable	
Report the **clinical trial registration number** (at ClinicalTrials.gov or equivalent), where applicable.	Not Applicable	

Laboratory protocol	Information included in the manuscript?	In which section is the information available? (Reagents and Tools Table, Materials and Methods, Figures, Data Availability Section)
Provide DOI OR other citation details if **external detailed step-by-step protocols** are available.	Not Applicable	

Experimental study design and statistics	Information included in the manuscript?	In which section is the information available? (Reagents and Tools Table, Materials and Methods, Figures, Data Availability Section)
Include a statement about **sample size** estimate even if no statistical methods were used.	Yes	Captions
Were any steps taken to minimize the effects of subjective bias when allocating animals/samples to treatment (e.g. **randomization procedure**)? If yes, have they been described?	Not Applicable	
Include a statement about **blinding** even if no blinding was done.	Not Applicable	
Describe **inclusion/exclusion criteria** if samples or animals were excluded from the analysis. Were the criteria pre-established?<br><br>If sample or data points were omitted from analysis, report if this was due to attrition or intentional exclusion and provide justification.	Yes	Materials and Methods
For every figure, are **statistical tests** justified as appropriate? Do the data meet the assumptions of the tests (e.g., normal distribution)? Describe any methods used to assess it. Is there an estimate of variation within each group of data? **Is** the variance similar between the groups that are being statistically compared?	Yes	Captions and Materials and Methods

Sample definition and in-laboratory replication	Information included in the manuscript?	In which section is the information available? (Reagents and Tools Table, Materials and Methods, Figures, Data Availability Section)
In the figure legends: state number of times the experiment was **replicated** in laboratory.	Yes	Captions
In the figure legends: define whether data describe **technical or biological replicates**.	Yes	Captions

Ethics

Ethics	Information included in the manuscript?	In which section is the information available? (Reagents and Tools Table, Materials and Methods, Figures, Data Availability Section)
Studies involving **human participants**: State details of **authority granting ethics approval** (IRB or equivalent committee(s), provide reference number for approval.	Not Applicable	
Studies involving **human participants**: Include a statement confirming that **informed consent** was obtained from all subjects and that the experiments conformed to the principles set out in the WMA Declaration of Helsinki and the Department of Health and Human Services Belmont Report.	Not Applicable	
Studies involving **human participants:** For publication of **patient photos**, include a statement confirming that consent to publish was obtained.	Not Applicable	
Studies involving experimental **animals**: State details of **authority granting ethics approval** (IRB or equivalent committee(s), provide reference number for approval. Include a statement of compliance with ethical regulations.	Not Applicable	
Studies involving **specimen and field samples:** State if relevant **permits** obtained, provide details of authority approving study; if none were required, explain why.	Not Applicable	

Dual Use Research of Concern (DURC)	Information included in the manuscript?	In which section is the information available? (Reagents and Tools Table, Materials and Methods, Figures, Data Availability Section)
Could your study fall under dual use research restrictions? Please check biosecurity documents and list of **select agents and toxins** (CDC): https://www.selectagents.gov/sat/list.htm	Not Applicable	
If you used a select agent, is the security level of the lab appropriate and reported in the manuscript?	Not Applicable	
If a study is subject to dual use research of concern regulations, is the name of the **authority granting approval and reference number** for the regulatory approval provided in the manuscript?	Not Applicable	

Reporting

The MDAR framework recommends adoption of discipline-specific guidelines, established and endorsed through community initiatives. Journals have their own policy about requiring specific guidelines and recommendations to complement MDAR.

Adherence to community standards	Information included in the manuscript?	In which section is the information available? (Reagents and Tools Table, Materials and Methods, Figures, Data Availability Section)
State if relevant guidelines or checklists (e.g., **ICMJE, MIBBI, ARRIVE, PRISMA**) have been followed or provided.	Not Applicable	
For **tumor marker prognostic studies,** we recommend that you follow the **REMARK** reporting guidelines (see link list at top right). See author guidelines, under 'Reporting Guidelines'. Please confirm you have followed these guidelines.	Not Applicable	
For **phase II and III randomized controlled trials**, please refer to the **CONSORT** flow diagram (see link list at top right) and submit the CONSORT checklist (see link list at top right) with your submission. See author guidelines, under 'Reporting Guidelines'. Please confirm you have submitted this list.	Not Applicable	

Data Availability

Data availability	Information included in the manuscript?	In which section is the information available? (Reagents and Tools Table, Materials and Methods, Figures, Data Availability Section)
Have **primary datasets** been deposited according to the journal's guidelines (see 'Data Deposition' section) and the respective accession numbers provided in the Data Availability Section?	Yes	data availability section
Were **human clinical and genomic datasets** deposited in a public access-controlled repository in accordance to ethical obligations to the patients and to the applicable consent agreement?	Not Applicable	
Are **computational models** that are central and integral to a study available without restrictions in a machine-readable form? Were the relevant accession numbers or links provided?	Not Applicable	
If publicly available data were reused, provide the respective **data citations in the reference list**.	Yes	Methods
